# De novo-designed ribozyme-controlled riboregulator for cell-free diagnostics

**DOI:** 10.1038/s41467-026-71684-6

**Published:** 2026-04-08

**Authors:** Yu Tang, Jie Luo, Ben Niu, Binbin Xie, Yaling Yuan, Hongzhao Yang, Shuang Zhao, Ping Huang, Zuowei Xie, Jing Sheng, Ruijia Deng, Jingsen Cao, Jiaqi Liu, Meilin Gong, Shuang Xie, Ming Chen, Kai Chang

**Affiliations:** 1 Department of Clinical Laboratory Medicine, Southwest Hospital, Third Military Medical University (Army Medical University), Chongqing, China; 2 Department of Neurology, Chongqing Mental Health Center, Chongqing, China; 3 Department of Clinical Laboratory, the Seventh People’s Hospital of Chongqing, Chongqing, China; 4 State Key Laboratory of Trauma and Chemical Poisoning, Army Medical University, Chongqing, China

**Keywords:** Synthetic biology, Lab-on-a-chip, Riboswitches, Ribozymes

## Abstract

Cell-free systems have great potential for nucleic acid assays, yet universal onsite diagnostics without preamplification are limited. Here, a universal cell-free diagnostic platform termed TRACKer (Target-Responsive non-preAmplification Cell-free diagnostic Kit) has been developed for detecting multiple respiratory viral RNAs in laboratory and field settings. TRACKer integrates three modules: ribozyme allostery module, riboregulator activation module, and output module. The ribozyme allostery module enables universal target recognition through strand displacement-mediated ribozyme conformational switching. The riboregulator activation module achieves preamplification-free detection via cascade expression of reporter proteins. The output module provides swappable reporter templates for luminescent quantification and lateral flow visualization in diverse scenarios. TRACKer is a robust system that enables rapid ( < 70 min) nucleic acid detection without preamplification and demonstrates attomolar sensitivity (1-10 aM) for six respiratory viruses. Overall, TRACKer presents a promising approach to nucleic acid detection, offering a low-cost and scalable solution with potential applications in point-of-care diagnostics and beyond.

## Abstract (web summary)

Cell-free systems hold potential for nucleic acid assays but are limited by the need for pre-amplification. Here the authors develop a cell-free diagnostic platform that detects viral RNA without pre-amplification demonstrating uses on six respiratory viruses in under 70 minutes with attomolar sensitivity.

## Introduction

Cell-free diagnostic platforms have emerged as transformative tools for nucleic acid detection, offering rapid and field-deployable clinical diagnostic solutions. Notably, RNA-based regulators (riboregulators) are a promising frontier to expand the horizon of cell-free diagnostic systems with desired specifications. To date, several riboregulators including toehold switches^1–4^, toehold repressor^5^, synthetic trans-acting riboswitch with triggering RNA (START)^6^, single-nucleotide-specific programmable riboregulators (SNIPRs)^7^, and riboswitches^8^, have been developed based on base-pairing mechanisms and inherent programmability (Supplementary Fig. 1). Among these designs, toehold switches are exemplary for their rapid kinetic properties and minimal metabolic burden in detecting a broad spectrum of nucleic acids, ranging from highly pathogenic agents, such as Zika virus^4,9^, Dengue virus^10^, and Ebola virus^11^, to complex microbial communities within the human gut microbiome^12^. Toehold switches-based diagnostics have garnered considerable interest and are poised to become the next-generation nucleic acid diagnostic tools. However, the design of toehold switches for different pathogens depends heavily on in silico modeling and laborious experimental fine-tuning, inherently limiting the universal applicability and scalability of these approaches.

Ribozymes are catalytic RNA molecules that perform sequence-specific cleavage reactions through their intricate tertiary structures, which integrate base-paired stems with conserved catalytic cores^13,14^. Owing to their compact size, structural simplicity, and modular versatility^13,15,16^, ribozymes exhibit remarkable sequence recognition and programmable cleavage activity, enabling groundbreaking applications in gene expression regulation^17–21^, intracellular molecular monitoring^15,22–24^, logic circuit design^25–27^, and cancer therapeutics^28,29^. Allosteric ribozymes represent a dynamic evolution of ribozymes, wherein their catalytic activity can be modulated by the presence of specific molecular signals (e.g. small molecules, ions, or proteins)^15,30,31^, through structural rearrangements of a catalytic core triggered by the allosteric domains. Despite their programmability, allosteric ribozymes are often constrained by a lack of orthogonality between the recognition and catalytic domains, leading to signal leakage and unintended off-target activation. This challenge arises from incomplete decoupling of the recognition and catalytic modules, limiting the scalability of these systems for complex applications. To achieve precise regulation of ribozyme activity, we proposed a strategy based on an “inhibition-recognition strand” (IRS). By stabilizing the catalytic core in an inactive conformation through programmable IRS sequences, the system effectively reduces background leakage. Furthermore, target-specific activation driven by sequence complementarity enables strict switch-like control with optimized orthogonality. Drawing inspiration from the above, this design strategy significantly boosts the combinatorial potential of the ribozyme system. It pioneers the way for the development of a universal platform for responsive RNA circuits, offering a solution to the generalizability bottleneck of conventional riboregulators and enhancing their utility in synthetic biology and precision diagnostics.

Herein, we present the Target-Responsive non-preAmplification Cell-free diagnostic Kit (TRACKer), an approach for nucleic acid detection employing the ribozyme-regulated cell-free protein synthesis (CFPS) system. TRACKer consists of three functional modules termed the ribozyme allostery module, riboregulator activation module, and output module. The ribozyme allostery module facilitates universal target recognition by leveraging strand displacement-mediated ribozyme conformational switching. The riboregulator activation module orchestrates preamplification-free detection by coupling target RNA binding with cascade reporter proteins expression. The output module incorporates modular reporter templates supporting both luminescent signal quantification for laboratory environments and colorimetric lateral flow readouts for onsite testing. Moreover, we integrated devices enabling direct sample processing validated across resource-limited settings. TRACKer enabled aM detection (1–10 aM) for six respiratory viruses in just 70 min during clinical assessment. TRACKer showed excellent concordance (88.9–100%) with RT-qPCR in detecting influenza A virus (FluA), respiratory syncytial virus (RSV), and human rhinovirus (HrV) across 97 clinical pharyngeal swab samples. Taken together, TRACKer paves the way for scalable cell-free diagnostics, delivering modular and cost-efficient solutions that hold potential for addressing nucleic acid detection challenges in multifaceted application scenarios.

## Results

### Working principle of TRACKer

**Fig. 1:**
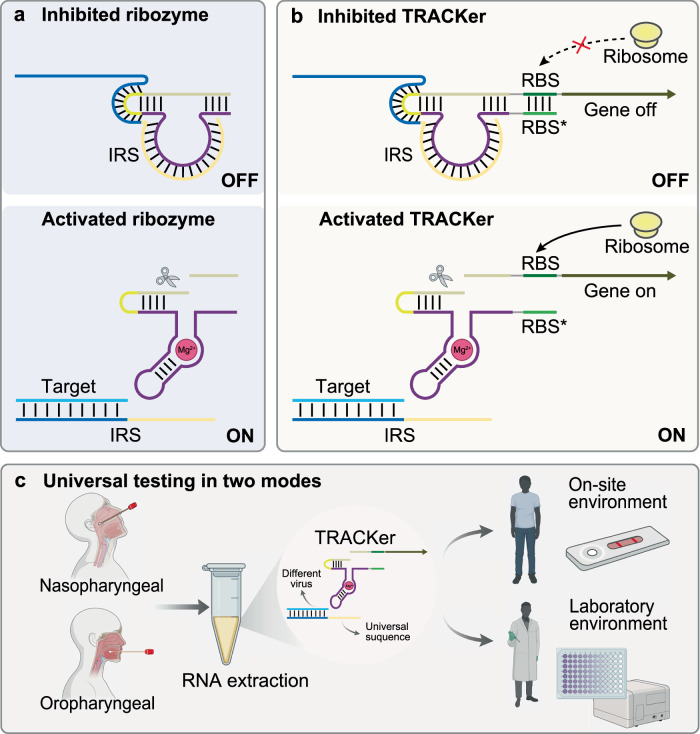
Overview of TRACKer. **a** Ribozyme OFF-to-ON transition: The ribozyme is inhibited (OFF) in the absence of target RNA and activated (ON) when target RNA binds, initiating RNA cleavage. **b** TRACKer activation via competitive strand displacement: target RNA binds IRS, outcompeting the switch strand and displacing IRS. This releases the ribozyme to cleave its substrate, exposing RBS and initiating reporter translation. **c** Versatile detection applications: TRACKer accommodates diverse targets through simple replacement of the IRS strand and the linker sequence connecting the ribozyme to its substrate, and supports both laboratory and onsite settings. RBS, RBS*, IRS represent ribosome-binding site, complementary sequences of ribosome-binding site, and inhibition-recognition strand, respectively. The illustrations of Fig. 1c were created with BioRender.com.

The TRACKer strategy combines ribozyme switching with complementary strand displacement within a single assay (Fig. 1a) to regulate cell-free reporter expression for nucleic acid detection. The assay is initiated by combining samples with a pre-assembled TRACKer mixture containing two RNA strands: an IRS strand and a switch strand. The IRS strand is composed of two parts: a ribozyme inhibition strand and a target recognition strand. The switch strand contains several essential elements: (1) a complementary sequence to the ribosome-binding site (RBS*), (2) a sequence identical to the first 12 bases of the target to link the ribozyme and its substrate, (3) a ribosome-binding site (RBS), and (4) a template sequence for reporter proteins.

The critical steps of the TRACKer mechanism are as follows (Fig. 1b and Supplementary Fig. 2): In the pre-assembled OFF state, the IRS hybridizes with the switch strand, locking the ribozyme in an inactive conformation. (i) The target RNA binds to the exposed recognition domain of the IRS via complementary base pairing. (ii) This binding trigger target-induced strand displacement: the sequence-specific complementarity between the target and IRS outcompetes the interaction between IRS and the switch strand, sequestering the IRS and driving its dissociation from the switch strand. This conformational rearrangement relieves ribozyme inhibition and restores its catalytic activity. (iii) The reactivated ribozyme cleaves its substrate, destabilizing the complementary RBS*-RBS duplex and exposing the RBS (Supplementary Fig. 3). (iv) Ribosomes then access the liberated RBS and initiate the translation of reporter proteins, generating measurable signals.

We established a straightforward workflow for detecting respiratory viruses from clinical pharyngeal swab samples (Fig. 1c). The entire TRACKer assay is completed within just 70 min after RNA extraction. Notably, the system supports versatile detection of various respiratory targets simply by substituting the target recognition strand of the IRS and the linker sequence connecting the ribozyme to its substrate. Moreover, its dual-mode output capability provides both luminescent and lateral flow readouts, supporting broad potential applicability across both laboratory and point-of-care scenarios.

### Molecular mechanism of TRACKer

**Fig. 2:**
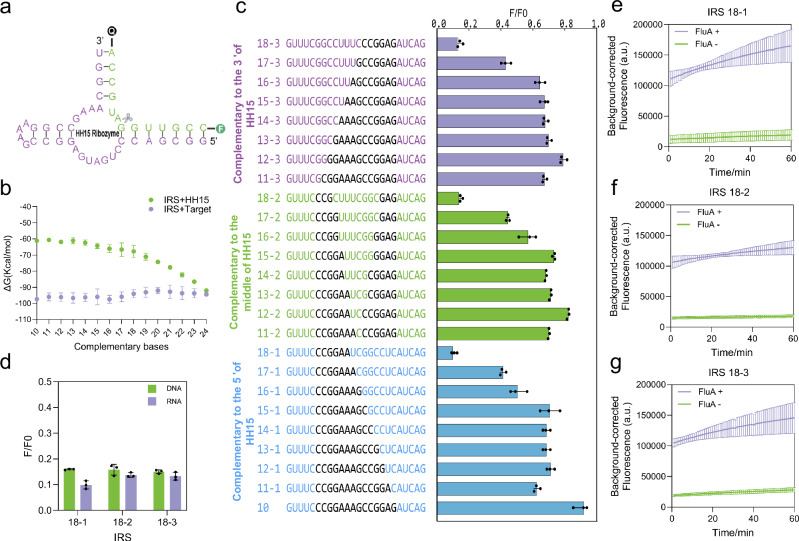
Inhibition-recognition strand (IRS) screening. **a** RNA motifs of the HH15 ribozyme. **b** Gibbs free energy changes of IRS (different complementary base numbers) binding to HH15 and the target. Each point is the average of energy changes for IRS (5’-end/ middle / 3’-end complementary) binding to HH15 and target, respectively. **c** Evaluation of the influence of the quantity and arrangement of complementary bases in the IRS on ribozyme inhibition. FAM and BHQ1 were placed at the terminal of the ribozyme substrate (Fig. 2a). The fluorescence signal without IRS served as the baseline (F0), while the signal following IRS addition was captured as F. The inhibition efficiency of each IRS was quantified by calculating the F/F0 ratio. Data are displayed as mean from technical triplicates. **d** Evaluation of inhibitory efficacy between RNA- and DNA-based IRS strands. Data are shown as mean ± standard deviation (s.d.) from technical triplicates. Fluorescence measurements for IRS18-1 (**e**), IRS18-2 (**f**), and IRS18-3 (**g**) targeting FluA. Fluorescence was not observed without IRS-mediated ribozyme inhibition (target-) but was observed following IRS recognition of the target (target + ). Shaded regions represent mean ± s.d. from technical triplicates. Source data are provided as a Source Data file.

TRACKer utilized the HH15 ribozyme with sequence-specific cleavage activity to construct a ribozyme-regulated riboregulator (Fig. 2a)^16,32^. Based on principles established by Haseloff and Gerlach^13^, we designed the IRS complementary to conserved catalytic core regions of HH15 (CUGAU and GAAAC; Supplementary Fig. 4). Enhancing complementarity between the IRS and HH15 improved ribozyme inhibition, but at the cost of reduced strand displacement efficiency. NUPACK simulations indicated that IRS constructs with 10-18 complementary nucleotides permitted efficient target-induced dissociation, whereas longer complements (19-24 nucleotides) promoted the formation of stable ternary complexes that impeded activation (Supplementary Fig. 5). Free energy calculations further rationalized this behavior (Fig. 2b, Supplementary Figs. 6-7): although the IRS-target binding energy remained largely unchanged, the IRS-HH15 interaction strengthened considerably as complementarity increased, rendering strand displacement kinetically impeded beyond 18 nucleotides. Accordingly, we propose that an IRS complementarity of 10-18 nucleotides to HH15 provides a functional guideline for the design and validation of TRACKer systems.

Guided by this design principle, we developed a fluorescence-based screening assay using a dual-labeled (FAM/BHQ1) ribozyme substrate to identify optimal IRS designs (Fig. 2a, Supplementary Data 1). Our screening revealed that inhibition efficiency was primarily determined by the length of complementary region between the IRS and the ribozyme, with the length of complementary region showing a positive correlation with inhibition efficiency. In contrast, the specific position of complementarity along the ribozyme sequence had relatively minor effects on inhibitory capacity (Fig. 2c). Three IRS with 18 complementary bases (IRS18-1/18-2/18-3), showed the highest inhibition efficiency. RNA-based IRS exhibited slightly stronger inhibition than DNA equivalents (Fig. 2d). Restoration of ribozyme activity and fluorescence signal upon target addition confirmed that the selected IRS sequences provide both effective inhibition in the absence of target and specific activation in its presence (Fig. 2e–g).

The competitive strand-displacement mechanism of TRACKer has been previously established by *Zhang* and *Winfree* (Supplementary Fig. 8). The linker between the ribozyme and the substrate (yellow region) serves as the initiation site for branch migration during competitive strand-displacement reactions. NUPACK-predicted equilibrium complex concentrations demonstrate the occurrence of competitive strand displacement upon target addition (Supplementary Fig. 9). Native polyacrylamide gel electrophoresis also confirms that target binding induces competitive strand displacement, leading to the formation of the target and IRS 18-1 duplex and release of the HiBiT switch (Supplementary Fig. 10).

To elucidate IRS-mediated ribozyme inhibition and target recognition, we performed molecular dynamics simulations using Gromacs2021. In system 1, the ribozyme and substrate formed a stable secondary structure essential for ribozyme cleavage activity (Supplementary Fig. 11a). Meanwhile, in system 2, IRS disrupted this structure, thereby preventing ribozyme cleavage activity (Supplementary Fig. 11b). Upon the addition of target RNA in system 3, the IRS dissociated from the ribozyme, allowing the ribozyme-substrate complex to reestablish the secondary structure observed in system 1 and restore the cleavage activity of the ribozyme (Supplementary Fig. 11c). Free energy calculations (gmx_MMPBSA)^33^ revealed the order of binding strength: system 1 > system 3 > system 2, indicating that IRS weakens ribozyme-substrate interactions, partially reversed by target binding (Supplementary Table 1). Time-dependent distance changes and probability density analyses confirmed IRS dissociation upon target introduction (Supplementary Fig. 11d-e).

In addition, we explored the active mechanism of TRACKer through molecular dynamics simulations. In the absence of target, the switch stand and IRS maintained a stable duplex. The target RNA hybridized with the IRS, triggering the dissociation of the IRS from the switch strand and restoring the activity of the ribozyme (Supplementary Fig. 12a). Target binding increased structural flexibility, particularly in the ribozyme domain (Bases 9-42), as indicated by elevated Root mean square fluctuation values (Supplementary Fig. 12b). Consistent with this, higher Root mean square deviation and radius of gyration values confirmed global conformational rearrangement and decreased compactness (Supplementary Fig. 12c-d). Increased solvent-accessible surface area further suggested enhanced exposure of functional regions in the activated state (Supplementary Fig. 12e). These simulations demonstrate that target-induced IRS displacement initiates structural unlocking of the ribozyme module, providing a mechanistic basis for TRACKer activation.

### De novo design and laboratory validation of TRACKer

**Fig. 3:**
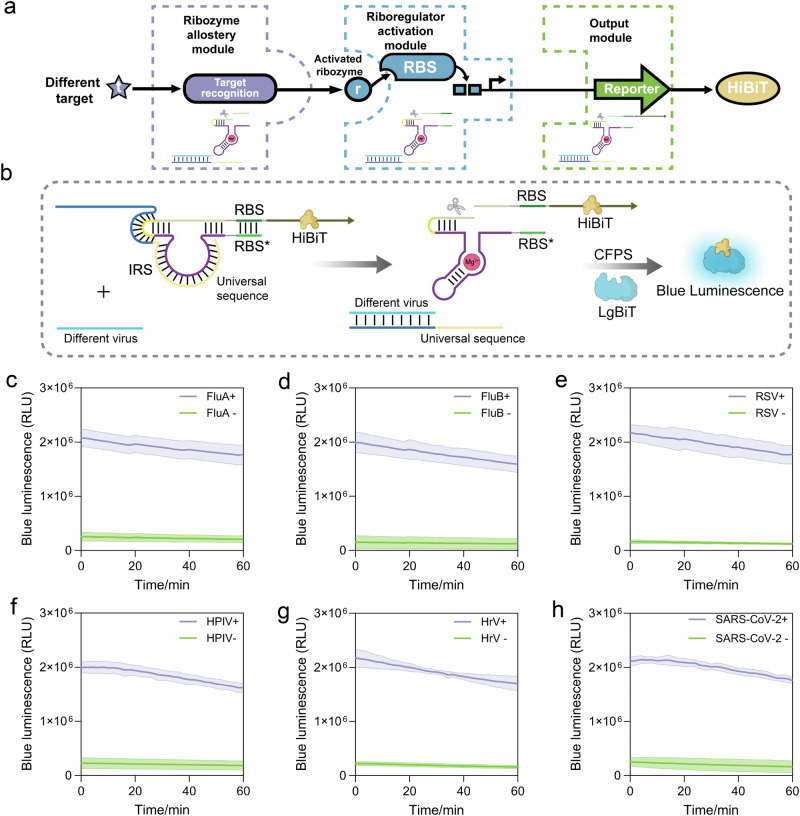
De novo design and validation of TRACKer. **a** Modular architecture of the TRACKer system. The three modules are composed of the ribozyme allostery module, riboregulator activation module, and output module. **b** Schematic of TRACKer activation via competitive strand displacement: target RNA binds IRS, outcompeting the switch strand and displacing IRS. This frees the ribozyme to cleave its substrate, exposing the RBS and enabling HiBiT translation. HiBiT complements with LgBiT to form active nanoluciferase, which converts furimazine into blue luminescence. Time-course luminescence measurements of TRACKer for detection of six different respiratory viruses: FluA (**c**), FluB (**d**), RSV (**e**), HPIV (**f**), HrV (**g**), and SARS-CoV-2 (**h**). Blue luminescence was measured continuously for 60 min following the completion of the cell-free protein synthesis reaction and the addition of furimazine. Shaded regions represent mean ± s.d. from technical triplicates. Source data are provided as a Source Data file.

Building on the molecular mechanism established above, we developed a de novo design framework for engineering target-specific TRACKer systems. TRACKer employs a modular architecture comprising three functionally distinct units: a ribozyme allostery module responsible for target recognition, a riboregulator activation module that translates ribozyme activity into cascade reporter proteins expression, and an output module that generates detectable signals (Fig. 3a, Supplementary Fig. 13). This division allows independent optimization and customization of each unit while maintaining system interoperability.

The small nanoluciferase subunit HiBiT was selected as the reporter protein due to its favorable biochemical properties: minimal structural interference, rapid folding kinetics, and efficient complementation-based luminescence. Functional luciferase activity is reconstituted upon HiBiT’s binding to its complementary partner LgBiT, enabling high-sensitivity detection at attomolar levels^34^. Figure 3b illustrates the versatile detection workflow using HiBiT as the reporter protein for diverse viruses in laboratory settings.

We optimized reaction conditions using FluA as a model target, determining that a 50:1 IRS-to-switch ratio, 31 °C incubation temperature, 40 mM Mg²⁺ concentration, and 40-min cell-free expression duration yielded optimal performance (Supplementary Fig. 14).

To verify the performance of the TRACKer system, we selected six common respiratory viruses reported in adults^35^: FluA, RSV, HrV, SARS-CoV-2, influenza B virus (Flu B), and human parainfluenza virus (HPIV). We retrieved the sequence structures of these viruses, identified their conserved regions (Supplementary Fig. 15, Supplementary Data 2), and simulated secondary structures using NUPACK^36^ (Supplementary Fig. 16). Regions that did not form complex secondary structures were selected as targets. The complementary sequences of these target regions and IRS18-1 were used to construct TRACKer for each virus. As illustrated in Fig.3c-h, each viral target sequence exhibited strong relative light unit (RLU) signals upon binding to the corresponding TRACKer, remaining stable for up to 1 h. Furthermore, the TRACKer constructed from IRS18-1 also exhibits a higher signal-to-noise ratio in the detection of FluA compared to other IRS (Supplementary Fig. 17).

To evaluate the broader applicability of TRACKer across diverse target types, we tested its performance in detecting structured RNA hairpins (C-23/29), microRNAs (miR-155 and miR-21), and bacterial antibiotic resistance genes (*KPC* and *NDM*). Target-specific IRS sequences were designed accordingly and integrated with the HiBiT switch to construct customized TRACKer systems. All customized TRACKers exhibited strong activation in response to their cognate targets, confirming the platform’s modularity and broad applicability (Supplementary Fig. 18).

### Detection capability of TRACKer

**Fig. 4:**
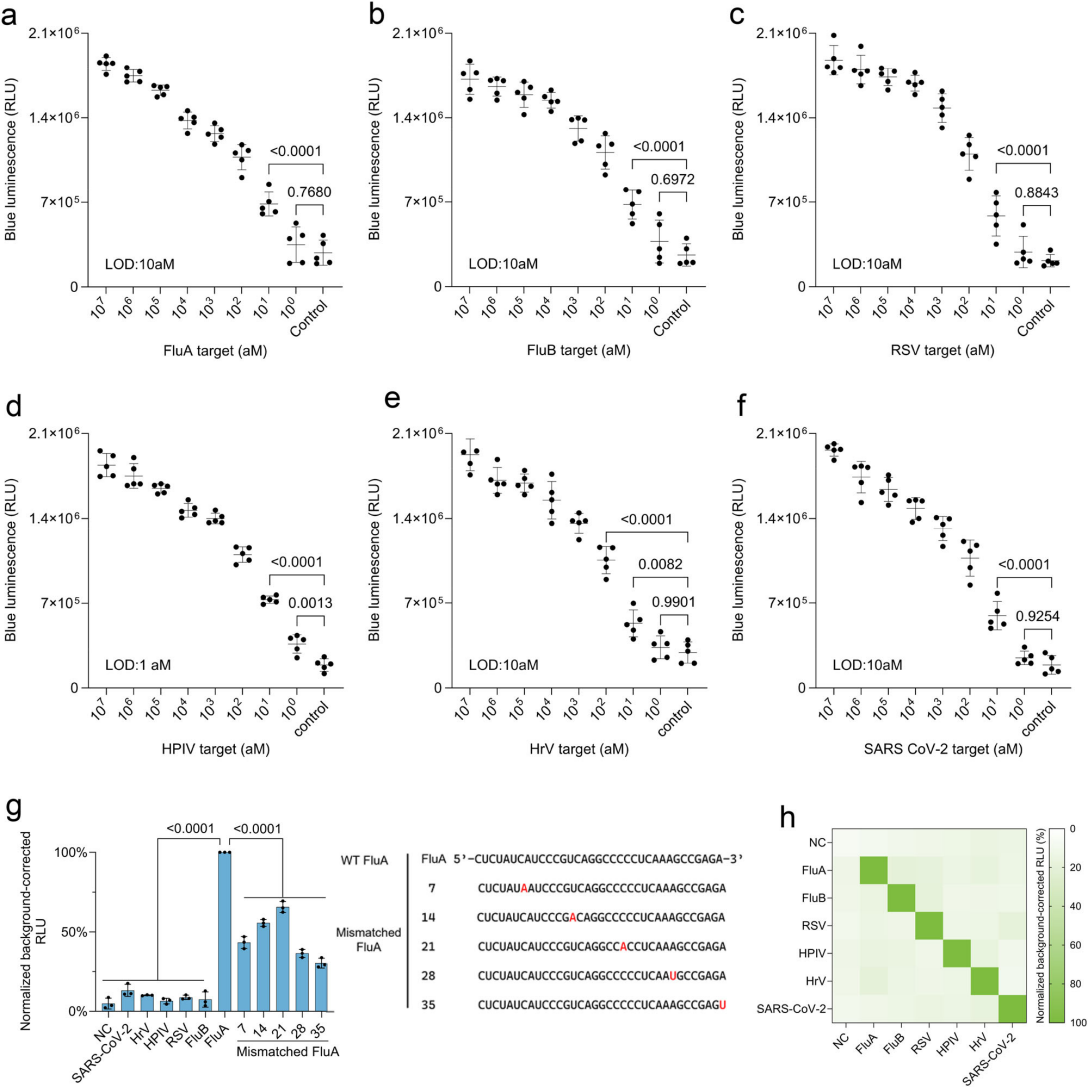
Detection capability of TRACKer for respiratory virus targets. Blue luminescence generated by TRACKer after incubation with different concentrations of FluA (**a**), FluB (**b**), RSV (**c**), HPIV (**d**), HrV (**e**) and SARS-CoV-2 (**f**). The LOD was defined as the lowest concentration showing statistically significant signal increase over the negative control (0 aM) by one-way ANOVA (p < 0.05). Data are shown as mean ± s.d. from five technical replicates. **g** TRACKer’s specificity for FluA. With luminescence normalized to FluA (100%), Mismatched FluA and other respiratory viruses (FluB, RSV, HPIV, HRV, and SARS-CoV-2) were examined. NC represents negative control. P values were calculated by one-way ANOVA with p < 0.0001. Data are shown as mean ± s.d. from technical triplicates. **h** The cross-activity of TRACKer was tested among six viral RNAs: FluA, FluB, RSV, HPIV, HrV, and SARS-CoV-2. Normalized background-corrected RLU values are displayed (R/R0, %), where R stands for RLU of non-cognate targets and R0 of fully orthogonal conditions. Data are shown as mean from technical triplicates. Source data are provided as a Source Data file.

TRACKer achieved high sensitivity by coupling the cascade amplification of cell-free protein synthesis with the HiBiT output mode. We evaluated the performance of TRACKer among six respiratory viruses: FluA, FluB, RSV, HPIV, HrV, and SARS-CoV-2. Titration experiments using synthetic viral RNA demonstrated that TRACKer reliably detected concentrations ranging from 1 aM to 10 fM, with luminescence intensity showing a strong concentration-dependent response across the entire range. The calculated limits of detection (LOD) for each virus were defined as the lowest concentration that yielded a statistically significant increase in luminescence signal compared to the negative control. For all six viruses, this threshold was 1–10 aM (one-way ANOVA, p < 0.0001; Fig. 4a–f). Furthermore, TRACKer robustly detected pseudovirus-encapsulated FluA *M1* gene fragments (Supplementary Fig. 19).

TRACKer exhibited high specificity, with signals from non-target viral RNAs remaining at baseline levels comparable to negative controls (Fig. 4g). To further characterize its specificity, we introduced different base mismatch into the FluA target, which elicited significantly reduced luminescence signals relative to the wild-type. Notably, the reduction in signal was more pronounced for mismatch located at the 5’ and 3’ termini compared to those in central regions (Fig. 4g). This positional effect likely arises from terminal mismatch inducing greater destabilization of IRS-target RNA hybridization, preventing the strand displacement necessary for ribozyme activation. Furthermore, TRACKer demonstrated excellent orthogonality, as evidenced by six independent systems targeting FluA, FluB, RSV, HPIV, HrV, and SARS-CoV-2. Each system exhibited strong activation only in response to its cognate viral RNA, with negligible cross-talk observed between non-cognate targets (Fig. 4h).

### Clinical detection of respiratory viruses using TRACKer

**Fig. 5:**
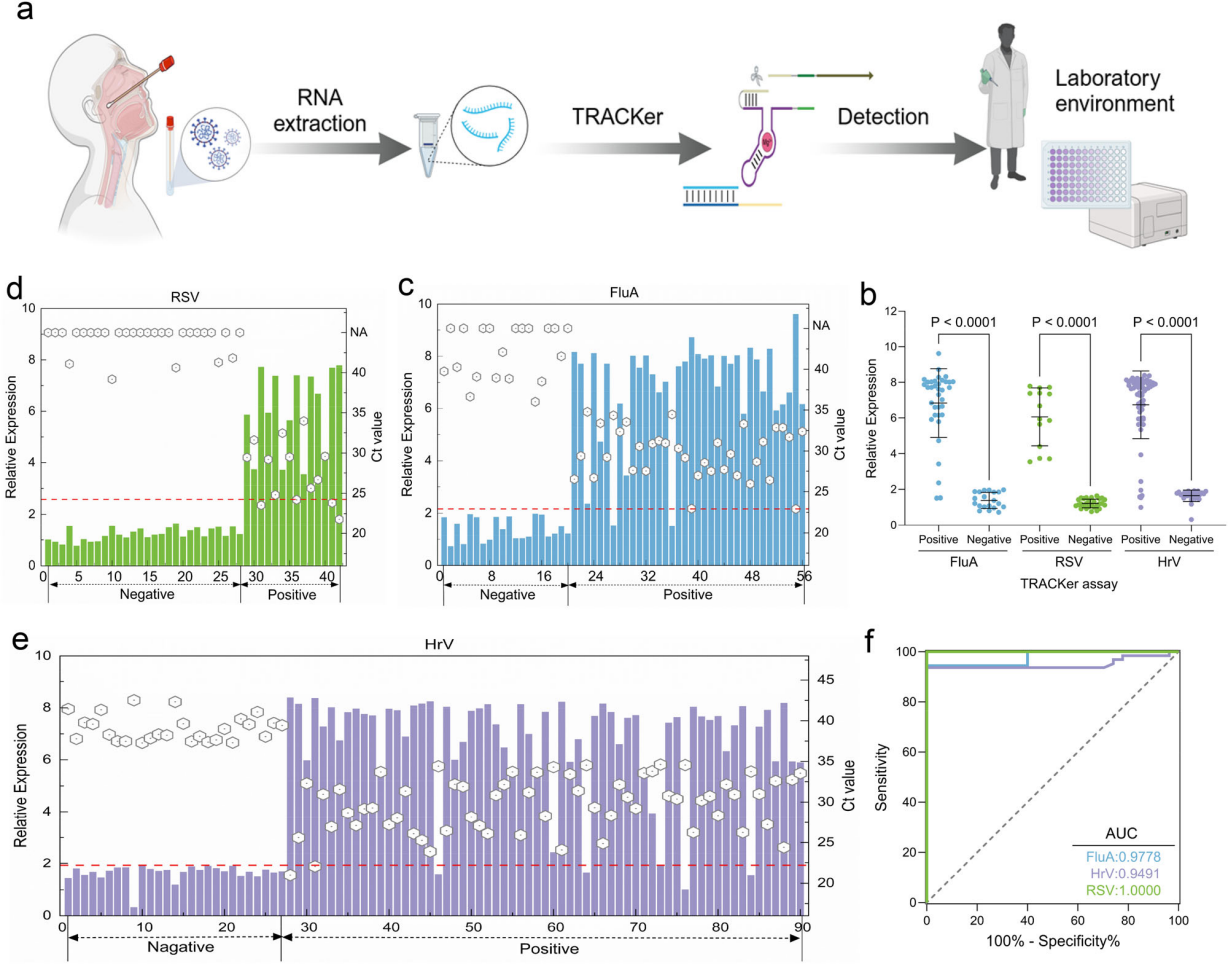
Clinical validation of the TRACKer assay in laboratory settings. **a** TRACKer workflow for detecting respiratory viral infections in clinical pharynx swab samples. **b** The relative expression levels of respiratory viruses detected by TRACKer in 188 clinical samples. P values were calculated by one-way ANOVA with p < 0.0001. The mean and standard deviation were denoted by the center line and error bars, respectively. **c–e** A comparative analysis of FluA(c), RSV(d), and HrV(e) was performed between RT-qPCR and TRACKer results. The relative expression levels (histograms), defined as the ratio of RLUgene to RLUnegative (where RLUgene and RLUnegative represent the RLU values for target viral genes and negative controls, respectively), along with RT-qPCR cycle threshold (Ct) values (shown as gray hexagons), are presented. The relative expression thresholds (red dashed lines) were determined using ROC curve analysis. **f** ROC curves that illustrated the diagnostic efficacy of TRACKer for 56 FluA, 48 RSV, and 90 HRV samples. NA in (**c**, **d**) indicates no ct value. Source data are provided as a Source Data file. The Illustrations of Fig. 5a was created with BioRender.com.

We evaluated the performance of TRACKer in detecting respiratory viruses using clinical pharyngeal swab samples (Fig. 5a). Samples were obtained from patients presenting with symptoms of upper respiratory tract infections, with relevant clinical characteristics summarized in supplementary Table 2. Based on reference RT-qPCR results, the sample cohort included 36 FluA-positive and 20 FluA-negative cases, 63 HrV-positive and 27 HrV-negative cases, and 14 RSV-positive and 28 RSV-negative cases (Supplementary Figs. 20–22).

All samples were analyzed using TRACKer targeting the *M1* gene (FluA), the 5’-UTR region (HrV), and the *N* gene (RSV). To account for background signal variation, TRACKer readings were normalized as the ratio of relative luminescence units for the target gene to those of negative controls (RLUgene/RLUnegative), which revealed a clear separation between positive and negative groups (p < 0.0001; Fig. 5b). Optimal diagnostic thresholds for FluA, HrV, and RSV detection were determined via receiver operating characteristic (ROC) curve analysis (Fig. 5c–f, Supplementary Table 3). An additional blinded validation was performed using 25 FluA, 35 HrV, and 37 RSV specimens (Supplementary Fig. 23a–c). Compared with RT-qPCR results (Supplementary Fig. 23d–f), TRACKer demonstrated a sensitivity of 100% and specificity of 94.8% for FluA; 90% sensitivity and 92% specificity for HrV; and 88.9% sensitivity and 96.4% specificity for RSV. These results underscore the strong clinical potential of the TRACKer platform (Supplementary Fig. 23g–i).

### Field-deployable TRACKer with lateral flow readout

To enable instrument-free nucleic acid detection in resource-limited environments, we developed a visual TRACKer system that operates at ambient temperature without specialized instrument. Inspired by the simplicity of rapid antigen tests, this approach employs short tag peptides to facilitate rapid cell-free expression while minimizing secondary structure interference. Building on previous reports that dual-epitope peptides reduce background signals^34^, we designed a reporter incorporating 3×Flag and 6×His epitopes separated by a 12-amino acid linker to minimize steric hindrance and enhance detection sensitivity.

**Fig. 6:**
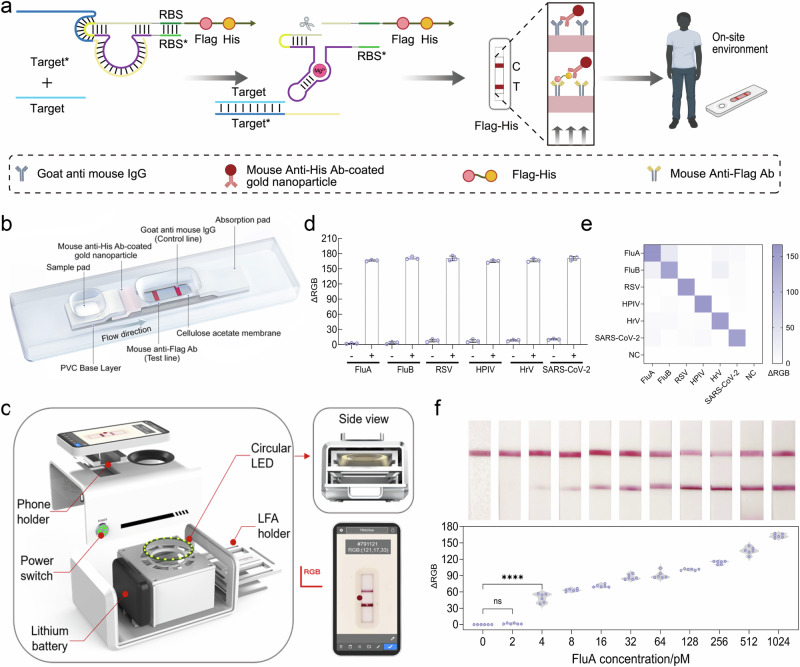
Practicality of TRACKer-LFA in field applications. **a** Workflow of TRACKer-LFA detection for the target. The LFA strip comprised a test line coated with mouse anti-Flag monoclonal antibody and a control line coated with goat anti-mouse IgG polyclonal antibody. In the presence of the target, TRACKer synthesized Flag-His-tagged peptides bound to a mouse anti-His monoclonal antibody conjugated with gold nanoparticles. The test line encapsulated the complex, producing a discernible red band. The control line verified the assay’s efficacy. **b** Schematic diagram of the LFA. **c** Integrated device for TRACKer-LFA tests. A lithium battery-powered circular LED, controlled via a switch, provides stable illumination for imaging. An adjustable LFA holder enables vertical positioning of the test strip. A smartphone mounted on a fixed holder captures images of the LFA. RGB analysis was performed through a mobile application. **d** TRACKer-LFA identified six respiratory viruses, namely FluA, FluB, RSV, HPIV, HrV, and SARS-CoV-2. Data are shown as mean ± s.d. from technical triplicates. **e** TRACKer-LFA demonstrated orthogonality in respiratory virus detection for different viruses. Data are shown as mean from technical triplicates. **f** A bar chart of the ΔRGB values and representative pictures for the TRACKer-LFA detection of FluA at different concentrations (****P  <  0.0001, One-way ANOVA). Data are shown as mean ± s.d. from six technical replicates. Source data are provided as a Source Data file. The Illustrations of Fig. 6a was created with BioRender.com.

As shown in Fig. 6a, the detection mechanism employs dual-epitope reporter proteins compatible with diverse RNA targets, enabling visual readouts through lateral flow assays (LFAs). The LFA strips integrate three critical components: a test line coated with mouse anti-Flag monoclonal antibodies, a control line with goat anti-mouse IgG polyclonal antibodies, and a conjugate pad containing colloidal gold-labeled mouse anti-His monoclonal antibodies. When target RNA triggers TRACKer-mediated synthesis of the dual-epitope peptide, the peptide binds to gold-conjugated anti-His antibodies during lateral flow migration. These complexes are captured at the test line by anti-Flag antibodies, generating a visible red band. Unbound gold antibodies subsequently bind to anti-IgG antibodies at the control line, producing a validation band. This room-temperature process delivers naked-eye results within minutes, making it particularly suitable for field diagnostics. The LFA strip architecture incorporates a sample pad, antibody-coated nitrocellulose membrane, absorbent pad, and gold conjugate pad laminated onto a PVC backing and enclosed within a protective plastic casing for enhanced stability and portability (Fig. 6b).

We further developed an integrated field detection device featuring a battery-powered circular LED for uniform illumination, an adjustable strip holder for precise alignment, and a smartphone-mounted camera for image capture (Fig. 6c, Supplementary Fig. 24). A custom mobile application quantified the results through ΔRGB analysis, defined as *ΔRGB* = *√[(R* − *R₀) ²* + *(G* − *G₀) ²* + *(B* − *B₀) ²]*, where R, G, B and R0, G0, B0 represent RGB values of test samples and negative controls, respectively. Using IRS18-1 designed for six respiratory targets, TRACKer-LFA specifically detected FluA, FluB, RSV, HPIV, HrV, and SARS-CoV-2 (Fig. 6d). Orthogonality testing confirmed target-specific activation with negligible cross-reactivity observed across viral RNAs (Fig. 6e). Repeatability testing demonstrated consistent detection of FluA down to 4 pM (Fig. 6).

Furthermore, we validated the performance of TRACKer-LFA using simplified sample preparation. A total of 26 swab specimens were concurrently tested by RT-qPCR and TRACKer-LFA. Based on normalized RGB signals, TRACKer-LFA demonstrated 92.3% (12/13) positive percent agreement and 100% (13/13) negative percent agreement with RT-qPCR for FluA detection (Supplementary Fig. 25).

## Discussion

TRACKer is a modular and scalable cell-free diagnostic platform that enables rapid, preamplification-free target detection with high sensitivity and dual-mode outputs, with designed potential for both laboratory and field applications. Unlike conventional riboregulators that rely on target-induced unfolding of stem-loop structures and require extensive redesign for each new target, TRACKer employs an intermediary IRS strand. Target binding displaces the IRS, activating the ribozyme which in turn cleaves its substrate to release the RBS and initiate translation. This mechanism allows for rapid reprogramming and minimizes experimental optimization (Supplementary Fig. 1).

The specific-site cleaving ribozyme mechanism holds broader implications for engineering RNA-based synthetic systems. By integrating IRS-mediated target recognition with ribozyme activation, this strategy establishes a programmable framework adaptable to diverse RNA architectures, including dynamically regulated riboregulators, RNA-activated CRISPR guide RNAs^37–40^, and ligand-responsive aptamer systems^41,42^. Such modular RNA designs could be further enhanced via engineered catalytic cascades to amplify detection signals, addressing scenarios demanding ultra-high sensitivity. For example, one promising approach involves engineering ribozyme substrates so their cleavage products act as secondary triggers to activate additional ribozyme switches, forming cyclic amplification loops that exponentially boost signal output. Another strategy entails designing reporter genes with small RNA motifs that, once expressed, displace IRS from ribozymes through positive feedback, creating self-amplifying cycles that sustain and amplify responses to low-abundance targets.

The output module of HiBiT-based luminescence and Flag-His LFA supports adaptable detection with potential application across both laboratory and field scenarios. The luminescent readout enabled attomolar sensitivity (1–10 aM) suitable for quantitative laboratory analysis, whereas the visual LFA was suitable for field use without instrumentation. Because target recognition and cascade reporter proteins expression are mediated by the upstream ribozyme allostery module and riboregulator activation module, the output module of these two detection methods do not compromise the system’s orthogonality. In addition, compared with other cell-free diagnostic platforms^1,4,12,34,43–45^, TRACKer exhibits superior simplicity, speed, and sensitivity (Supplementary Table 4), highlighting its potential as a scalable and adaptable cell-free diagnostic platform.

Despite these promising results, several limitations of our approach should be noted. Discrepancies between computational predictions and experimental outcomes persist as a challenge, particularly since NUPACK simulations do not account for non-canonical tertiary interactions^46^, which may alter energy balance and CFPS efficiency. Furthermore, the ionic complexity of cell-free reaction environments greatly exceeds the simplified 1 M NaCl conditions used in simulations, potentially leading to unpredictable effects on RNA folding kinetics and thermodynamics^47^. Although our structure-based targeting strategy is effective for all viral targets tested, the hybridization efficiency may be reduced by steric hindrance or partial occlusion in computationally accessible regions. The potential trouble makes experimental validation a necessary step to verify robust signal generation from in silico selected sites in TRACKer. Furthermore, our clinical validation of TRACKer-LFA was limited to viruses with moderate-to-high loads (Ct <30). Future efforts will focus on increasing the assay’s sensitivity to reliably detect low-load infections and meet the broader demands of field-deployable diagnostics.

TRACKer aims to contribute to molecular diagnostics in decentralized settings, where cold-chain infrastructure and specialized instrument are often limited. The CFPS reagent is lyophilization-compatible and retains full transcriptional and translational activity for up to one year at room temperature^11^. The implementation of a closed-tube reaction protocol and RNase-free consumables addresses the major challenge of RNase degradation for RNA-based field applications. The entire assay operates across ambient temperatures ranging from 22 to 37 °C, facilitating use in environments without precise thermal control. The integrated portable LED device standardizes result interpretation, thereby reducing subjectivity. The system delivers results within 70 min, which is significantly faster than conventional RT-qPCR. The estimated cost of TRACKer-LFA is $2.07 per test (Supplementary Table 5), making it competitive against other field-deployable methods (Supplementary Table 6). Collectively, TRACKer establishes a ribozyme-switched biosensing platform that enables preamplification-free, programmable nucleic acid detection with low cost, offering a versatile potential solution for both laboratory and point-of-care diagnostic needs.

## Methods

### Ethical statement

All procedures were performed in accordance with relevant ethical guidelines and regulations. This study was approved by the Ethics Committee of the First Affiliated Hospital of Army Medical University (approval number: (A)KY2025066). All studies followed the Declaration of Helsinki (2013) and the “Ethical Review Measures for Biomedical Research Involving Humans” (2016) issued by the National Health and Family Planning Commission of China. Discarded throat swabs were obtained under a waiver of informed consent, and the collection was confirmed not to constitute human subject research. No approval from the Ministry of Science and Technology of China (MOST) is required for the export of genetic information or materials in this study. All experiments and data analysis were conducted in compliance with relevant Chinese regulations.

### Materials

All DNA and RNA synthesis sequences were purchased from Sangon Biotech and purified via high-performance liquid chromatography. CFPS assay kits were obtained from Suzhou Perotin Biotechnology. RNA extraction (cat. no. 52904) was conducted using Qiagen reagents. The LgBiT subunit and substrate furimazine were sourced from Promega (cat. no. N3030). Flag-His tag protein detection test strips (cat. no. BF06219) were purchased from Biodragon. The T7 High Yield RNA Synthesis Kit (cat. no. B639253), TE buffer (pH 7.4) (cat. no. B548106), RNase-free ddH₂O (cat. no. B541018), Acrylamide (cat. no. A601032), 10,000 × 4S Red Plus Nucleic Acid Stain (cat. no. A606695), 50 × Tris-acetate-EDTA (TAE) buffer (cat. no. B548101), and 5 × Tris-boric acid-EDTA (TBE) buffer (cat. no. B548102) were all purchased from Sangon Biotech. The PrimeScript™ FAST RT Kit (cat. no. RR092A) and TB Green® Premix Ex Taq™ II (Tli RNaseH Plus) Kit (cat. no. RR820A) were obtained from TaKaRa.

### Inhibition-recognition strand screening

The ribozyme reaction system was prepared as follows: Control group (F0): 5 μL HH15 ribozyme (2 μM), 5 μL ribozyme substrate chain (2 μM), 5 μL Tris-HCl (500 mM), and 5 μL ultrapure water. Experimental group (F): 5 μL IRS (2 μM) replaced the 5 μL water in the control group. All systems were heated at 90 °C for 3 min (denaturation and annealing) and incubated at 37 °C for 1 h in the presence of 10 μL MgCl₂ (100 mM). Fluorescence values were measured at the endpoint, and inhibition efficiency was calculated as (F/F0 × 100%). After screening the IRS with high inhibition efficiency (18-1/18-2/18-3), further competitive binding experiments were conducted by adding 5 μL target RNA and measuring fluorescence in the presence of the target.

### TRACKer and TRACKer-LFA construction

TRACKer is composed of two single-stranded RNAs: (1) the HiBiT switch, which contains the RBS*, the sequence identical to the first 12 bases of the target to link the ribozyme and its substrate, the RBS, and the HiBiT protein-coding sequence; (2) the IRS, which contains the inhibition strand (default IRS 18-1) and target recognition strand. In the TRACKer-LFA, the HiBiT switch is replaced by the Flag-His switch, including the Flag-His tag-coding sequence. The remaining components are identical. The HiBiT/Flag-His switch (400 nM) and IRS (20 μM) were mixed and slowly annealed from 95 °C to 25 °C for 1 h and then stored at 4 °C.

### MD simulation

Four RNA structures (ribozyme-substrate, IRS, target, hibit switch) were constructed using oxDNA from their respective sequences (Supplementary Data 1), optimized with Gromacs 2021, and saved as PDB files. Five simulation systems were built in Gromacs 2021: System 1 (ribozyme-substrate), System 2 (ribozyme-substrate+IRS), System 3 (ribozyme-substrate+IRS+target), System 4 (hibit switch+IRS), and System 5 (hibit switch+IRS+target). Each system was placed in an appropriately sized box, solvated with water, and neutralized with NaCl. All systems underwent energy minimization to remove steric clashes, followed by 100 ps NVT (298.15 K) and 100 ps NPT (1 atm) equilibrium simulations with position restraints using standard coupling methods. Production molecular dynamics simulations were run with a 2 fs time step, generating trajectories for 80 ns (Systems 1–3) and 100 ns (Systems 4–5).

### TRACKer target RNA detection

To detect target RNA, 2.4 μL of target RNA, 4.8 μL of probe (containing 400 nM HiBiT switch and 20 μM IRS), and 4.8 μL of MgCl₂ (40 mM) were incubated at 31 °C for 20 min. Subsequently, 37.2 μL of cell-free expression premix (20.5 μL A solution + 16.7 μL B solution) was added, and the reaction was carried out at 25 °C for 40 min to express HiBiT protein. Afterward, 50 μL of detection buffer (containing 0.5% LgBiT subunit and 1% furimazine) was added, and luminescence was measured (RLU, Varioskan LUX microplate reader).

### TRACKer-LFA target RNA detection

To detect target RNA, 2.4 μL of target RNA, 4.8 μL of probe (containing 400 nM Flag-His switch and 20 μM IRS), and 4.8 μL of MgCl₂ (40 mM) were incubated at 31 °C for 20 min. Subsequently, 37.2 μL of cell-free expression premix (20.5 μL A solution + 16.7 μL B solution) was added, and the reaction was carried out at 25 °C for 40 min to express Flag-His protein. Afterward, the reaction mixture was incubated with 50 μL of buffer on an LFA strip for 10 min. The LFA strip was loaded into an integrated device, and imaging was performed using a Huawei P60 smartphone. The RGB values were analyzed using Color Grab (Loomatix Technology, version 3.9.2), a freely available image-processing application, and signal intensity was quantified according to the following formula

$$\Delta {RGB}=\surd [(R-{R}_{0})^{2}+{(G-{G}_{0})}^{2}+{(B-{B}_{0})}^{2}]$$

Here, R, G, and B represent the test values, and R₀, G₀, and B₀ correspond to the negative control values.

### Viral RNA extraction

Viral RNA was extracted using the QIAamp® Viral RNA Mini Kit (QIAGEN, 52904) from 140 μL of clinical samples. Briefly, 560 μL of Buffer AVL containing carrier RNA was added to a microcentrifuge tube, followed by the sample. The mixture was incubated at room temperature for 10 min to lyse viral particles. After adding 560 μL of ethanol, the mixture was transferred to a QIAamp Mini column, centrifuged at 6000 × *g* for 1 min, and the filtrate was discarded. The lysate was loaded into the column, followed by two washes with 500 μL of Buffer AW1 and AW2. After a 3-min full-speed centrifugation, RNA was eluted with 60 μL of Buffer AVE, incubated for 1 min at room temperature, and centrifuged to collect the RNA. The eluted RNA was heated at 95 °C for 5 min before further analysis.

### RT-qPCR detection

cDNA was synthesized from 5 μL of clinical RNA samples using the PrimeScript™ FAST RT Kit. The reaction system for genome DNA removal consisted of 16 μL containing 2 μL of 8X gDNA Eraser Premix, RNA, and ultrapure water and was incubated at 42 °C for 2 min. Briefly, 4 µL of 5X RT Premix was added, and the reaction was performed at 37 °C for 10 min and at 85 °C for 5 s. qPCR was performed using the TB Green Premix Ex Taq II (Tli RNaseH Plus) Kit: 25 μL reaction volume containing 12.5 μL of 2X premix, 1 μL of forward/reverse primers (0.4 μM), 2.5 μL of cDNA, and 9 μL of ultrapure water. The reaction conditions were as follows: initial denaturation at 95 °C for 30 s, followed by 45 cycles of 95 °C for 5 s and 60 °C for 30 s.

### Clinical samples

Clinical pharynx swab samples were obtained from residual clinical samples after routine testing. After RNA extraction, the samples were aliquoted into 5 μL portions and stored at –80 °C. Samples were thawed on ice before use to avoid repeated freeze-thaw cycles.

### Statistics & reproducibility

Statistical analyses, including one-way ANOVA, two-tailed Student’s t test, and receiver operating characteristic (ROC) curve generation for evaluating TRACKer diagnostic accuracy, were conducted using GraphPad Prism (GraphPad Software, version 10.1.2). For the TRACKer and RT-qPCR assays used in clinical sample validation, one technical replicate was performed due to the limited availability of clinical samples. Sensitivity assays and pseudovirus validation assays were performed with five technical replicates, all other experiments were performed with three technical replicates. Detailed information on data presentation, sample size, and specific statistical methods is included in the corresponding figure legends. No statistical method was used to predetermine sample size. No data were excluded from the analyses. The experiments were not randomized. Investigators were blinded during the clinical sample validation phase of the TRACKer assay. Fluorescence kinetic curves and chemiluminescence kinetic curves are presented as the mean ± standard deviation (s.d) of three technical replicates.

### Reporting summary

Further information on research design is available in the Nature Portfolio Reporting Summary linked to this article.

## Supplementary information

Supplementary information

Peer Review File

Description of Additional Supplementary Files

Supplementary Data 1

Supplementary Data 2

Reporting Summary

## Source data

Source Data

## Data Availability

All data generated in this study are provided in this published article and its supplementary information files. The relevant DNA/RNA-seq data generated in this study have been deposited in the Supplementary Data 1 and 2. Source data are provided with this paper.

## Code availability

The proposed algorithm and scripts to screen for IRS is available on GitHub (https://github.com/tanglab-coder/TRACKer-IRS-code).The specific version of the code associated with this publication is archived in Zenodo and is accessible via (10.5281/zenodo.19019005)^48^.
