# Supplementary material for: De novo-designed ribozyme-controlled riboregulator for cell-free diagnostics
Source: Nat Commun. 2026 Apr 8;17:6189. doi: 10.1038/s41467-026-71684-6 (PMC13369928; doi:10.1038/s41467-026-71684-6)
Supplement: Supplementary file 1 — Supplementary information [file 41467_2026_71684_MOESM1_ESM.pdf]

Supplementary Information

De novo-designed ribozyme-controlled riboregulator for cell-free diagnostics

Yu Tang[1,#], Jie Luo[1,#], Ben Niu[1,#], Binbin Xie[2], Yaling Yuan[3], Hongzhao Yang[1], Shuang Zhao[1], Ping Huang[1], Zuowei Xie[1], Jing Sheng[1], Ruijia Deng[1], Jingsen Cao[1], Jiaqi Liu[1], Meilin Gong[1], Shuang Xie[1], Ming Chen[1*], Kai Chang[1,4*]

[1]Department of Clinical Laboratory Medicine, Southwest Hospital, Third Military Medical University (Army Medical University), Chongqing, 400038, China

[2]Department of Neurology, Chongqing Mental Health Center, Chongqing, 401147, China

[3]Department of Clinical Laboratory, the Seventh People's Hospital of Chongqing, Chongqing, 400054, China

[4]State Key Laboratory of Trauma and Chemical Poisoning, Army Medical University, Chongqing, 400038, China

[#]These authors contributed equally: Yu Tang, Jie Luo, Ben Niu

*Corresponding Authors:

Kai Chang, changkai0203@tmmu.edu.cn,

Ming Chen, chming1971@126.com.

Contents

Supplementary Fig. 1 | Comparison of TRACKer with conventional riboregulators........................ S-4

Supplementary Fig. 2 | Molecular unlocking mechanism of TRACKer... S-5

Supplementary Fig. 3 | FRET-based assessment of TRACKer conformational changes upon target binding. S-6

Supplementary Fig. 4 | Design principles for the inhibition-recognition strand (IRS)...................... S-7

Supplementary Fig. 5 | NUPACK-predicted equilibrium complex concentrations among target, HH15 ribozyme and IRS of different complementary lengths S-8

Supplementary Fig. 6 | NUPACK-predicted secondary structure and Gibbs free energy between HH15 ribozyme and IRS of different complementary lengths S-10

Supplementary Fig. 7 | NUPACK-predicted secondary structure and Gibbs free energy between target and IRS of different complementary lengths S-13

Supplementary Fig. 8 | Strand displacement mechanism of TRACKer based on the principle of toehold exchange S-16

Supplementary Fig. 9 | NUPACK-predicted equilibrium complex concentrations of (a, HiBiT switch + IRS 18-1) and (b, HiBiT switch + IRS 18-1 + FluA). S-17

Supplementary Fig. 10 | Native Polyacrylamide gel electrophoresis analysis for the strand displacement manchanism of TRACKer S-18

Supplementary Fig. 11 | Mechanistic insights into IRS-mediated ribozyme inhibition and target recognition. S-19

Supplementary Fig. 12 | Mechanistic insights into TRACKer activation....................................... S-20

Supplementary Fig. 13 | Schematic diagrams of TRACKer activation. S-21

Supplementary Fig. 14 | Optimization of reaction conditions for TRACKer. S-22

Supplementary Fig. 15 | Viron images of six respiratory virus targets... S-23

Supplementary Fig. 16 | Secondary structures of viral RNA predicted by NUPACK S-25

Supplementary Fig. 17 | Endpoint luminescence and Signal-to-noise ratio of TRACKer for FluA detection with IRS16-1, 17-1, 18-1, 19-1, and 20-1 .... S-26

Supplementary Fig.18| Time-course luminescence measurements of TRACKer for detection of six different target: C-23(a), C-29(b), miR-155(C), miR-21(d), KPC(e), and NDM(f)....................... S-27

Supplementary Fig.19 | Detection capability of TRACKer for pseudovirus harboring the FluA M1 gene in a laboratory setting S-28

Supplementary Fig.20 | RT-qPCR detection of influenza A (FluA) virus in 56 clinical pharynx swab samples S-29

Supplementary Fig.21 | RT-qPCR detection of Human rhinovirus (HrV) in 90 clinical pharynx swab samples S-30

Supplementary Fig. 22| RT-qPCR detection of respiratory syncytial virus (RSV) in 42 clinical pharynx swab samples S-31

Supplementary Fig. 23| Clinical validation of TRACKer's diagnostic performance S-32

Supplementary Fig. 24 | Attachment device S-33

Supplementary Fig. 25 | Diagnostic performance of TRACKer-LFA with simplified sample preparation S-34

Supplementary Table. 1 | Free Energy Analysis for systems 1, 2, and 3 .... S-35

Supplementary Table. 2 | Clinical Information of Cohorts Used for Detecting Influenza A (FluA), Respiratory Syncytial Virus (RSV), and Human Rhinovirus (HrV) S-35

Supplementary Table. 3 | Predictive agreement between TRACKer and RT-qPCR for clinical samples. S-36

Supplementary Table. 4 | Comparison of TRACKer with other cell-free detection methods S-36

Supplementary Table. 5 | Estimated cost per TRACKer reaction S-37

Supplementary Table. 6 | Comparison of TRACKer with other field-deployable methods S-37

Supplementary References S-38

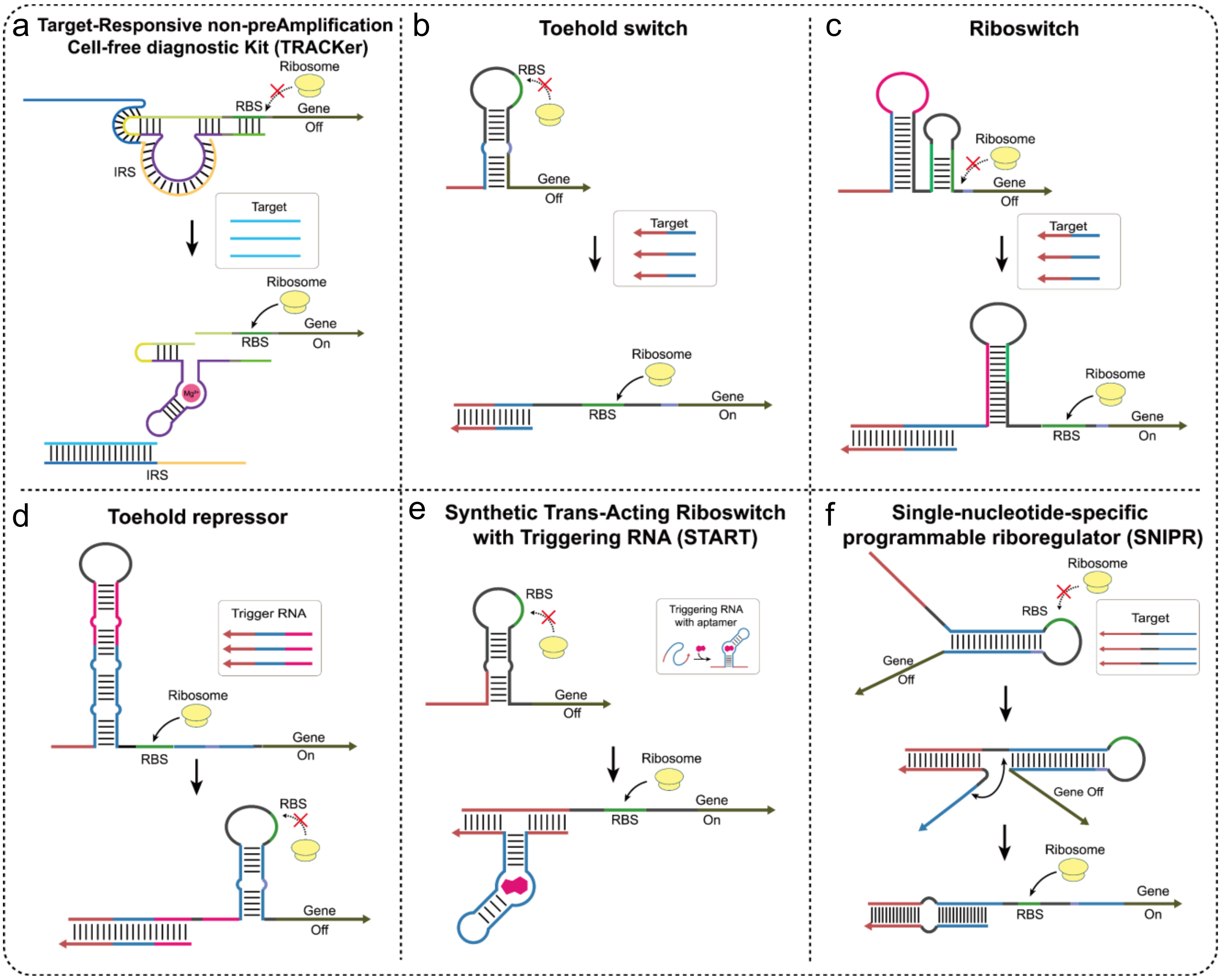

Supplementary Fig. 1 | Comparison of TRACKer with conventional riboregulators.

a, TRACKer: the target displaces the IRS, which in turn activates ribozyme. This activated ribozyme cleaves the substrate and creates nicks, enabling the release of the RBS and the downstream reporter gene sequence. **b**, Toehold switch[1]: the target binds to the toehold region, which opens the stem-loop and exposes the RBS to initiate gene expression. **c**, Riboswitch[2]: the target interacts with the toehold region, leading to the opening of the secondary structure of the RBS and thus triggering gene expression. **d**, Toehold repressor[3]: the target binds to the toehold region, resulting in the formation of a stem-loop at the RBS and the inhibition of gene expression. **e**, Synthetic trans-acting riboswitch with triggering RNA (START)[4]: the target binds to the aptamer, causing the stem-loop to open and the RBS to be exposed for the initiation of gene expression. **f**, Single-nucleotide-specific programmable riboregulator (SNIPR)[5]: the target RNA binds to the forward toehold region, promoting the branch migration reaction and disrupting the base pairing in the reverse toehold region. This process opens the stem-loop and exposes the RBS to activate gene expression.

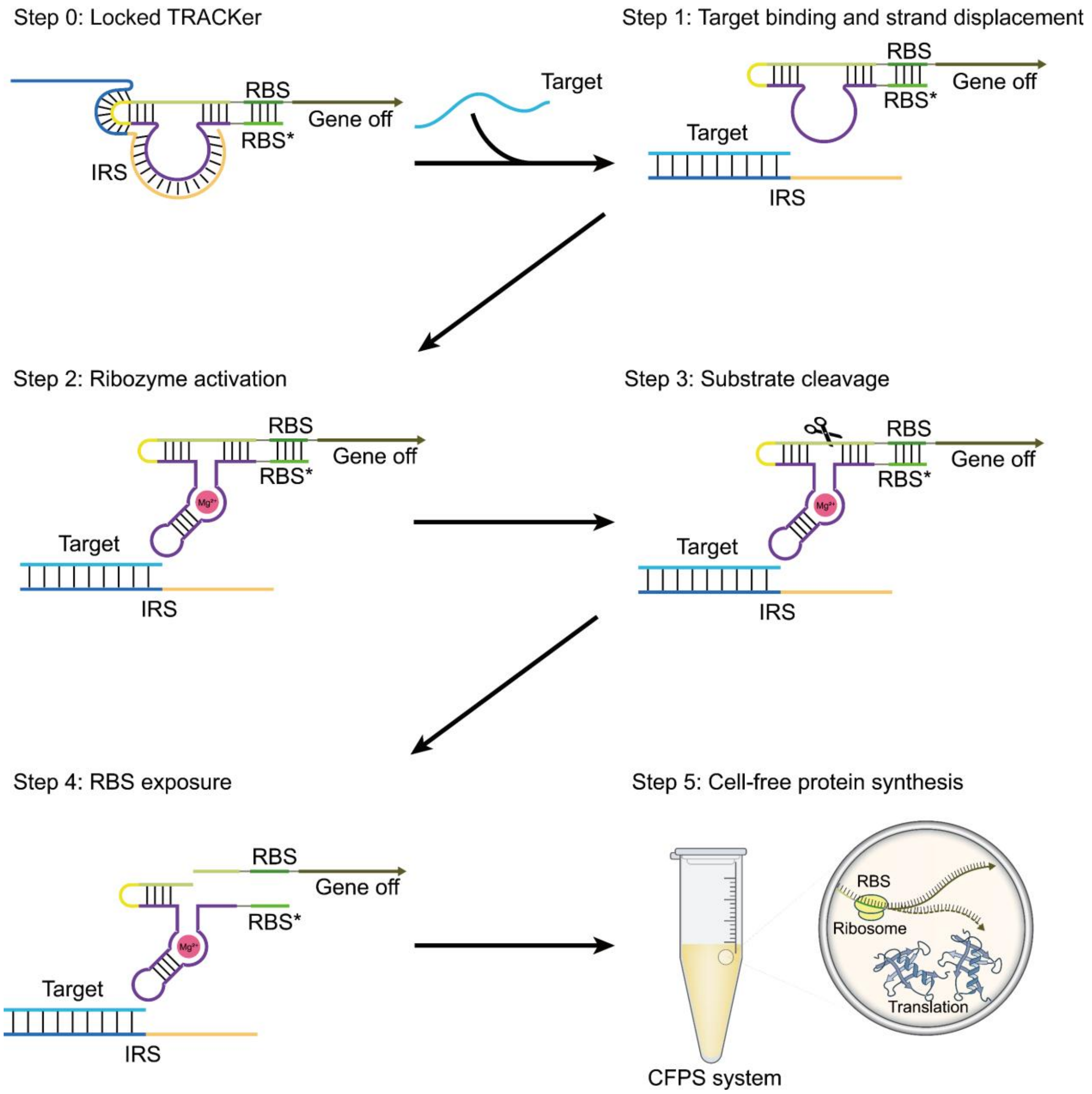

Supplementary Fig. 2 | Molecular unlocking mechanism of TRACKer.
Hybridization of the IRS sequence with the target triggers a strand displacement reaction, resulting in ribozyme activation. The activated ribozyme cleaves the substrate strand, thereby unmasking the RBS and initiating reporter protein expression.

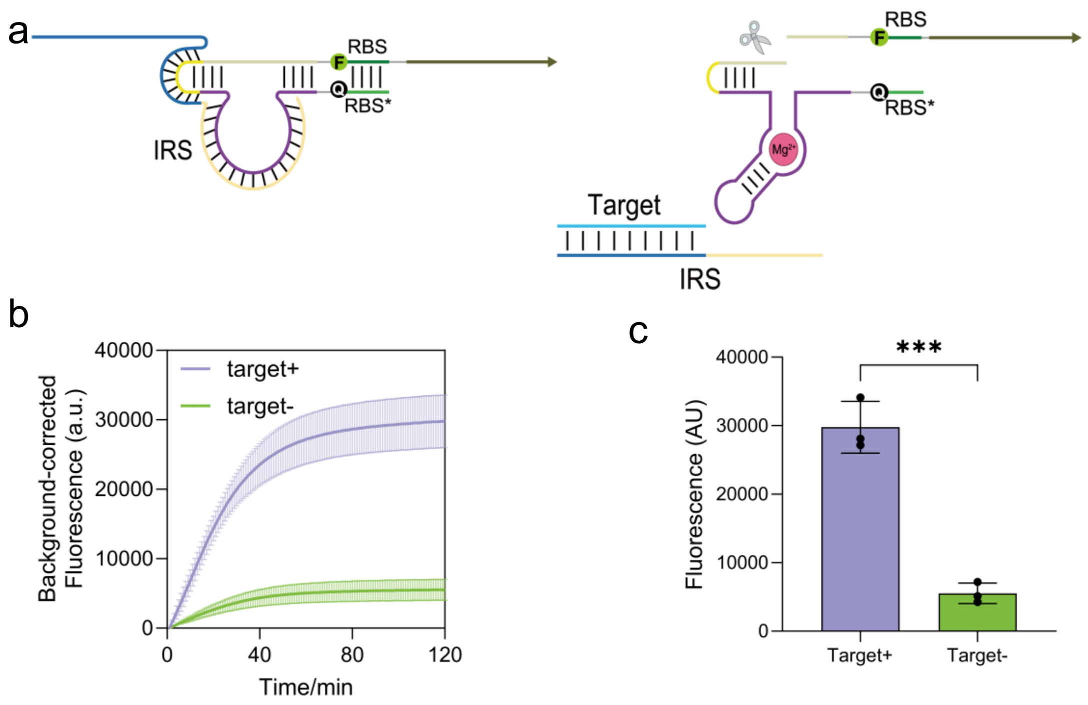

Supplementary Fig. 3 | FRET-based assessment of TRACKer conformational changes upon target binding.

a, Schematic illustration of the TRACKer design used to study molecular conformational changes following target addition, with fluorescein amidite (FAM) and black hole quencher 1 (BHQ1) labeled at the ribosome binding site (RBS) and RBS*, respectively. **b**, Time-dependent fluorescence curves in the presence or absence of the target. **c**, Endpoint fluorescence intensities with or without the target. Shaded regions represent the mean ± standard deviation (SD) from n = 3 technical replicates (***P = 0.0005; unpaired, two-tailed Student's t-test). Source data for this figure is available in the Source Data file.

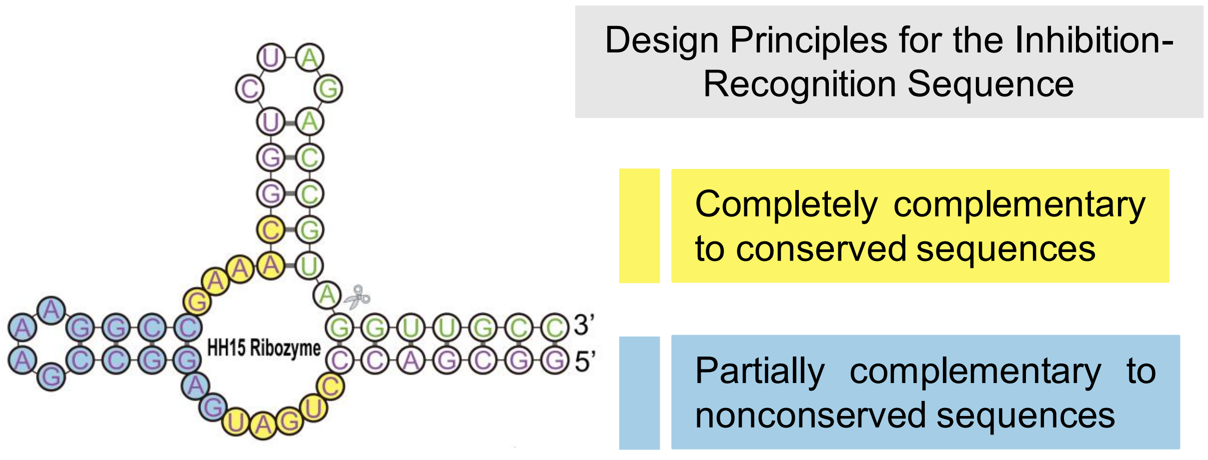

Supplementary Fig. 4 | Design principles of the inhibition-recognition strand (IRS).

The inhibition-recognition strand (IRS) consists of two functional elements: a strand that inhibits ribozyme activity and a strand that recognizes the target. The conserved sequence of the HH15 ribozyme is indicated by the yellow region, while the non-conserved sequence is represented by the blue region. For IRS construction, two core capabilities must be ensured: the ability to mediate a strand displacement reaction with the target, and the simultaneous ability to inhibit ribozyme activity. To achieve this, the first element (inhibition strand) is designed based on base complementarity principles, adopting a strategy of full complementarity to the ribozyme's conserved sequences and partial complementarity to its non-conserved sequences. The second element (recognition strand) is developed based on base complementarity to the target sequence.

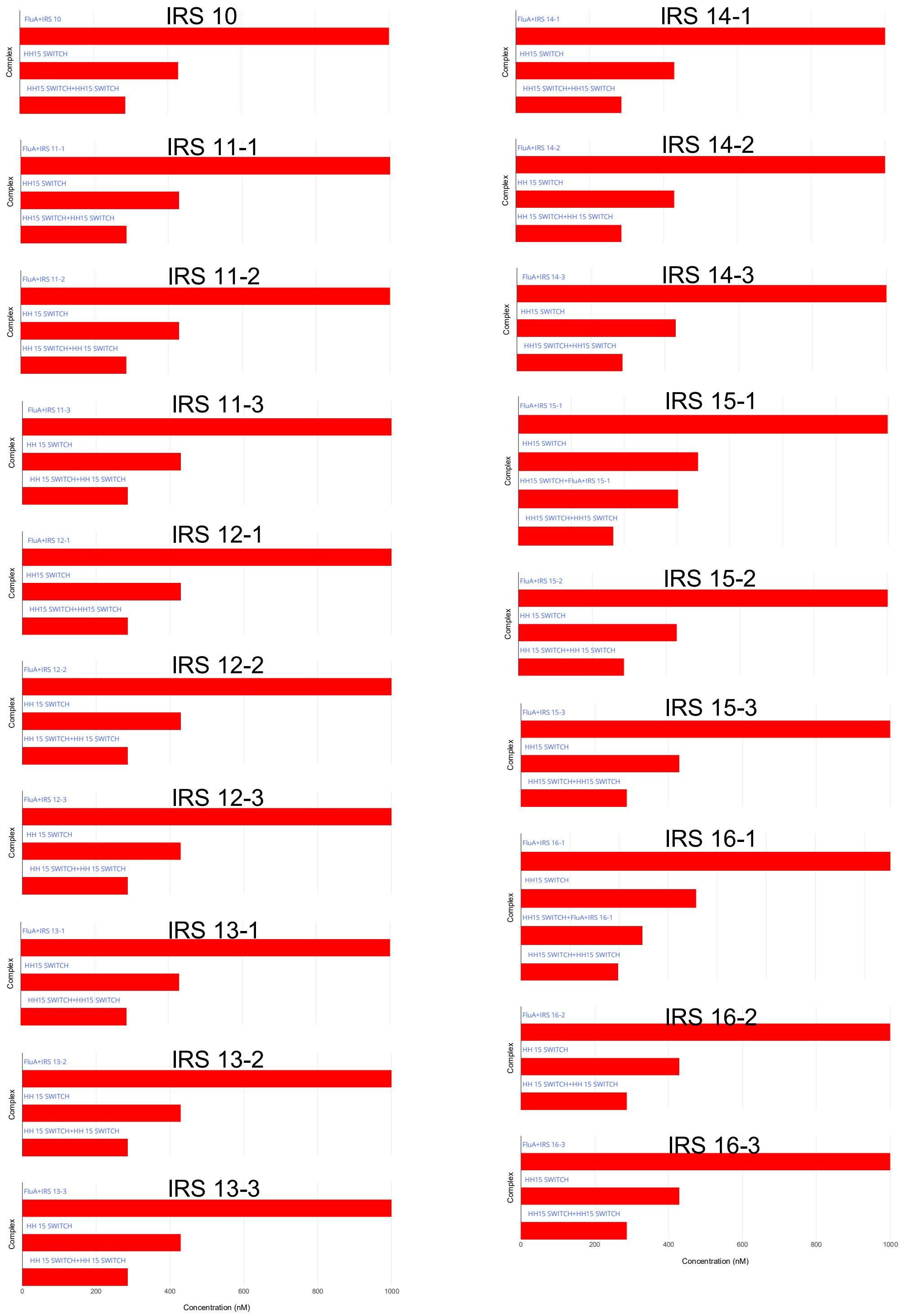
IRS 10
FluA+IRS 10
HH15 SWITCH
HH15 SWITCH+HH15 SWITCH
IRS 11-1
FluA+IRS 11-1
HH15 SWITCH
HH15 SWITCH+HH15 SWITCH
IRS 11-2
FluA+IRS 11-2
HH 15 SWITCH
HH 15 SWITCH+HH 15 SWITCH
IRS 11-3
FluA+IRS 11-3
HH 15 SWITCH
HH 15 SWITCH+HH 15 SWITCH
IRS 12-1
FluA+IRS 12-1
HH15 SWITCH
HH15 SWITCH+HH15 SWITCH
IRS 12-2
FluA+IRS 12-2
HH 15 SWITCH
HH 15 SWITCH+HH 15 SWITCH
IRS 12-3
FluA+IRS 12-3
HH 15 SWITCH
HH 15 SWITCH+HH 15 SWITCH
IRS 13-1
FluA+IRS 13-1
HH15 SWITCH
HH15 SWITCH+HH15 SWITCH
IRS 13-2
FluA+IRS 13-2
HH 15 SWITCH
HH 15 SWITCH+HH 15 SWITCH
IRS 13-3
FluA+IRS 13-3
HH 15 SWITCH
HH 15 SWITCH+HH 15 SWITCH
Complex
0
200
400
600
800
1000
Concentration (nM)
IRS 14-1
FluA+IRS 14-1
HH15 SWITCH
HH15 SWITCH+HH15 SWITCH
IRS 14-2
FluA+IRS 14-2
HH 15 SWITCH
HH 15 SWITCH+HH 15 SWITCH
IRS 14-3
FluA+IRS 14-3
HH15 SWITCH
HH15 SWITCH+HH15 SWITCH
IRS 15-1
FluA+IRS 15-1
HH15 SWITCH
HH15 SWITCH+FluA+IRS 15-1
HH15 SWITCH+HH15 SWITCH
IRS 15-2
FluA+IRS 15-2
HH 15 SWITCH
HH 15 SWITCH+HH 15 SWITCH
IRS 15-3
FluA+IRS 15-3
HH15 SWITCH
HH15 SWITCH+HH15 SWITCH
IRS 16-1
FluA+IRS 16-1
HH15 SWITCH
HH15 SWITCH+FluA+IRS 16-1
HH15 SWITCH+HH15 SWITCH
IRS 16-2
FluA+IRS 16-2
HH 15 SWITCH
HH 15 SWITCH+HH 15 SWITCH
IRS 16-3
FluA+IRS 16-3
HH15 SWITCH
HH15 SWITCH+HH15 SWITCH
Complex
0
200
400
600
800
1000
Concentration (nM)

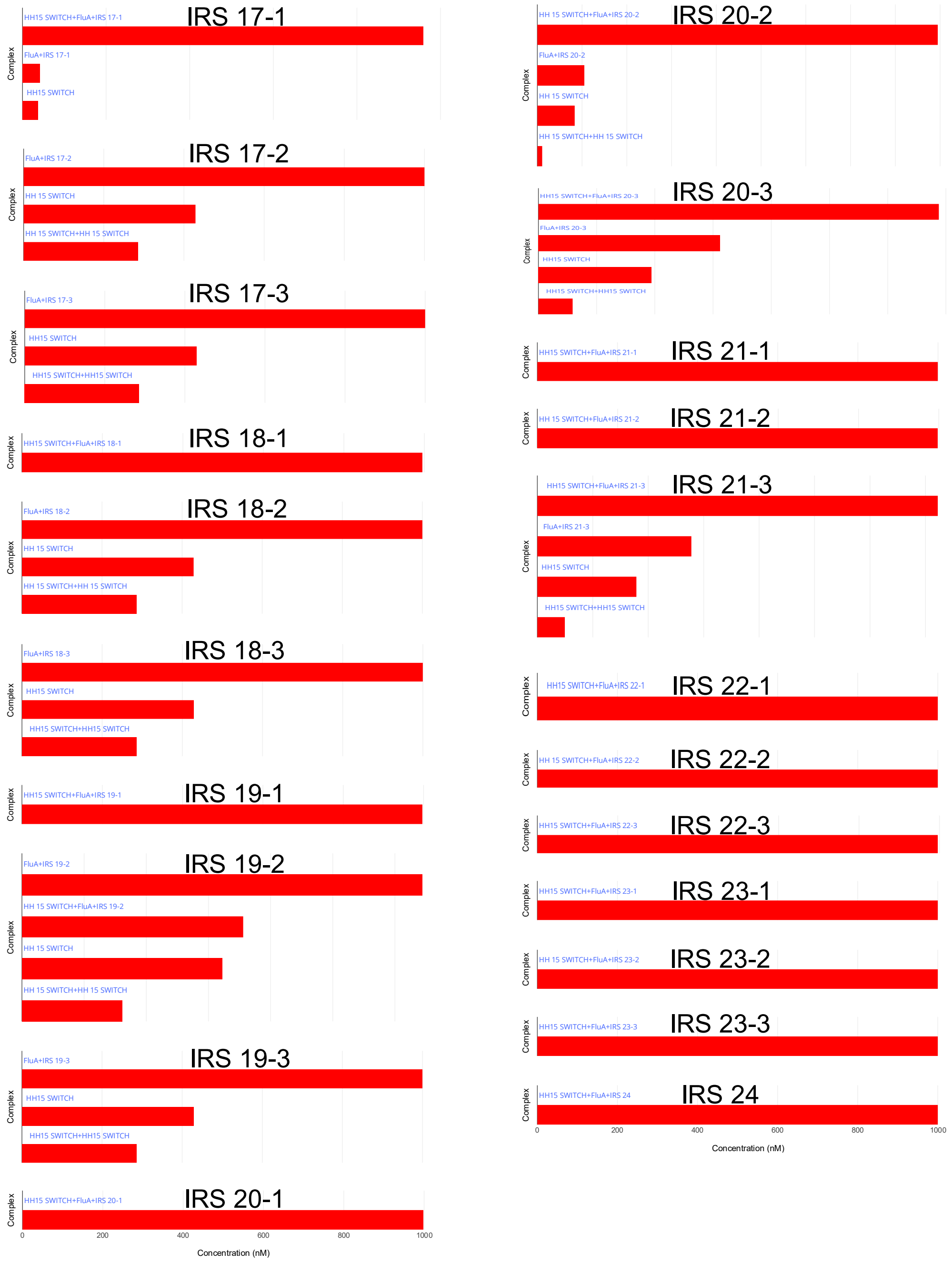

Supplementary Fig.5 | NUPACK-predicted equilibrium complex concentrations among target, HH15 ribozyme and IRS of different complementary lengths.

The structure was predicted using NUPACK based on the conditions as follows: The nucleic acid type was chosen to be ‘RNA’; the ‘dangles’ parameter was set to ‘all’; the temperature was set to 31 °C (the temperature at which TRACKer assay was performed).

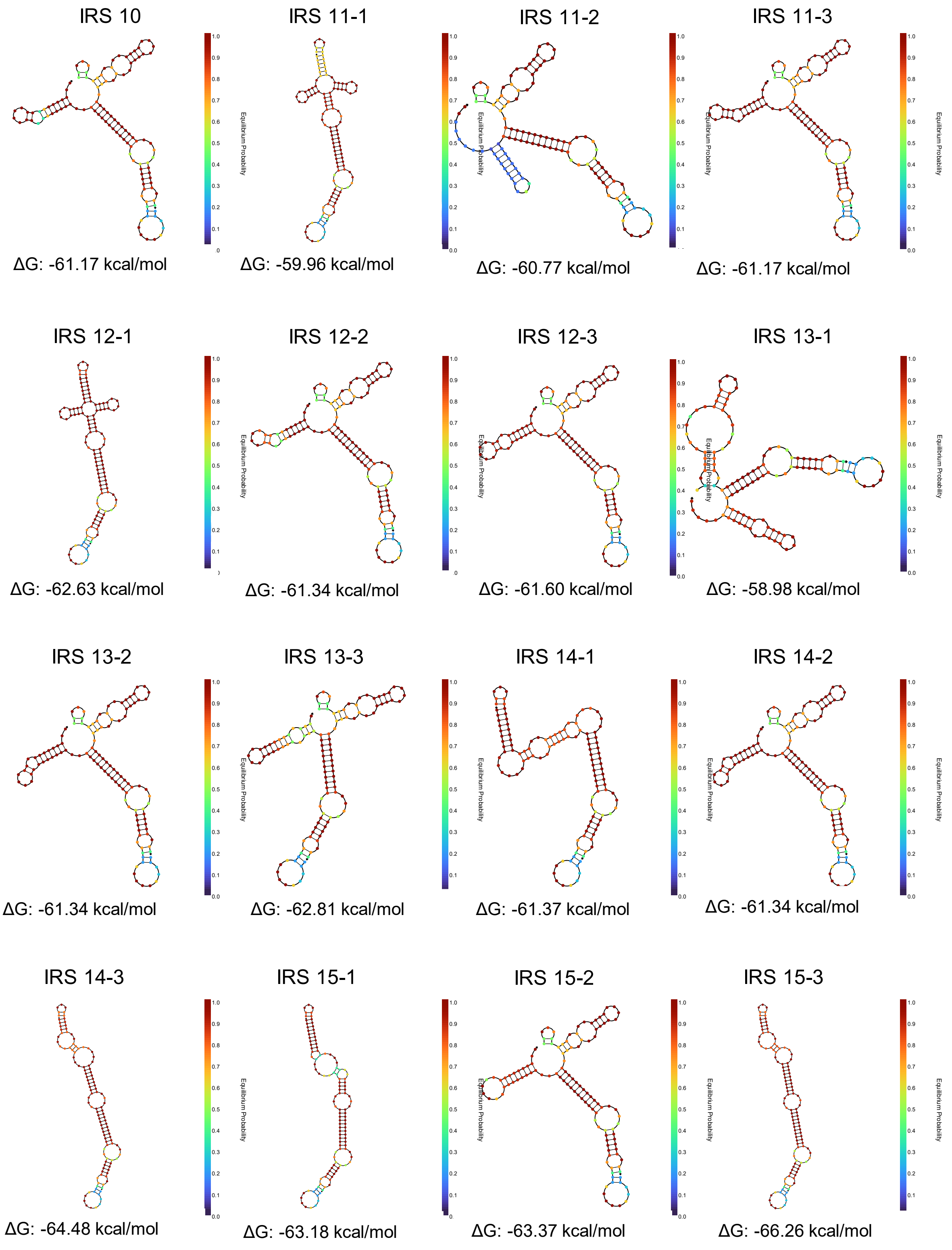
IRS 10
ΔG: -61.17 kcal/mol
IRS 11-1
ΔG: -59.96 kcal/mol
IRS 11-2
ΔG: -60.77 kcal/mol
IRS 11-3
ΔG: -61.17 kcal/mol
IRS 12-1
ΔG: -62.63 kcal/mol
IRS 12-2
ΔG: -61.34 kcal/mol
IRS 12-3
ΔG: -61.60 kcal/mol
IRS 13-1
ΔG: -58.98 kcal/mol
IRS 13-2
ΔG: -61.34 kcal/mol
IRS 13-3
ΔG: -62.81 kcal/mol
IRS 14-1
ΔG: -61.37 kcal/mol
IRS 14-2
ΔG: -61.34 kcal/mol
IRS 14-3
ΔG: -64.48 kcal/mol
IRS 15-1
ΔG: -63.18 kcal/mol
IRS 15-2
ΔG: -63.37 kcal/mol
IRS 15-3
ΔG: -66.26 kcal/mol
Equilibrium Probability

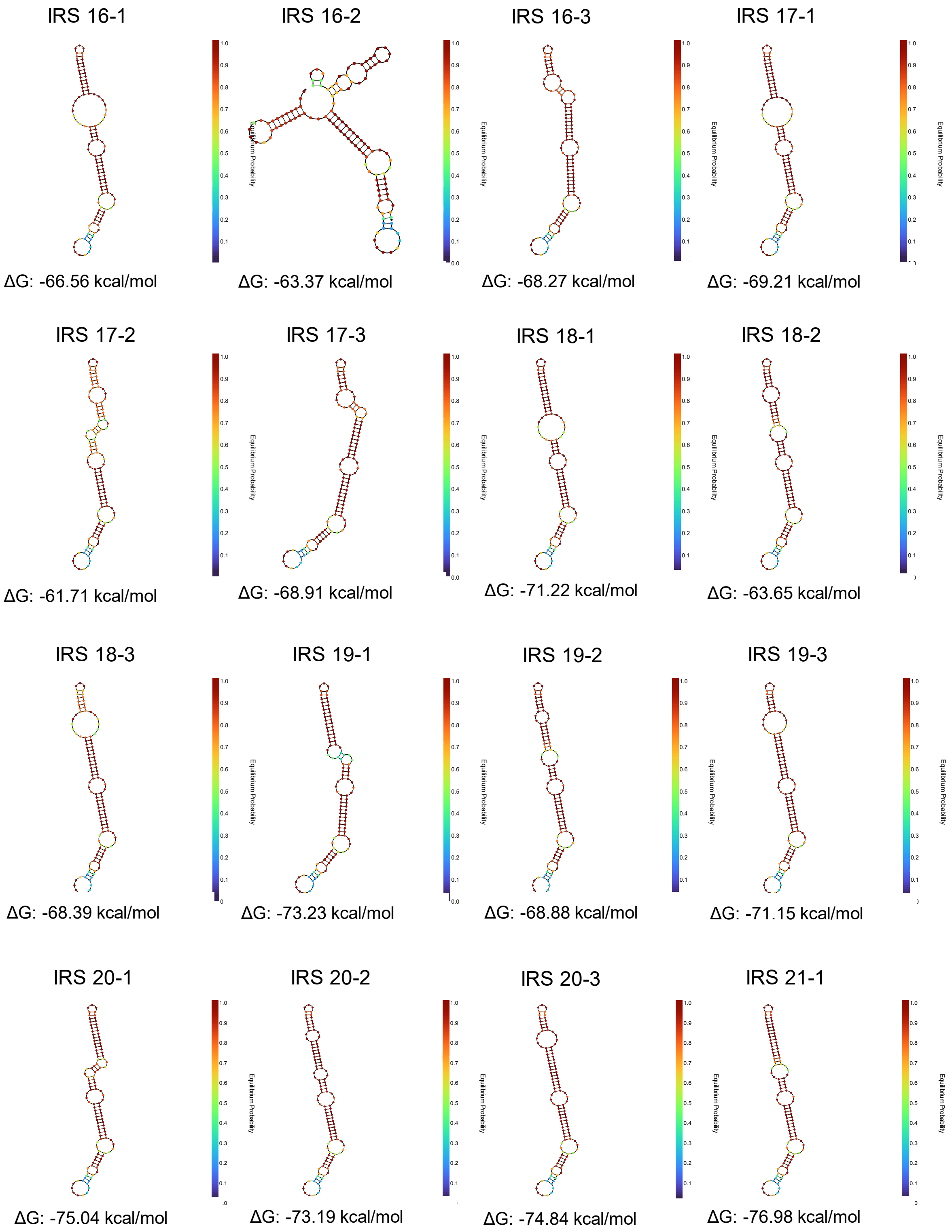
IRS 16-1
ΔG: -66.56 kcal/mol
IRS 16-2
ΔG: -63.37 kcal/mol
IRS 16-3
ΔG: -68.27 kcal/mol
IRS 17-1
ΔG: -69.21 kcal/mol
IRS 17-2
ΔG: -61.71 kcal/mol
IRS 17-3
ΔG: -68.91 kcal/mol
IRS 18-1
ΔG: -71.22 kcal/mol
IRS 18-2
ΔG: -63.65 kcal/mol
IRS 18-3
ΔG: -68.39 kcal/mol
IRS 19-1
ΔG: -73.23 kcal/mol
IRS 19-2
ΔG: -68.88 kcal/mol
IRS 19-3
ΔG: -71.15 kcal/mol
IRS 20-1
ΔG: -75.04 kcal/mol
IRS 20-2
ΔG: -73.19 kcal/mol
IRS 20-3
ΔG: -74.84 kcal/mol
IRS 21-1
ΔG: -76.98 kcal/mol
Equilibrium Probability

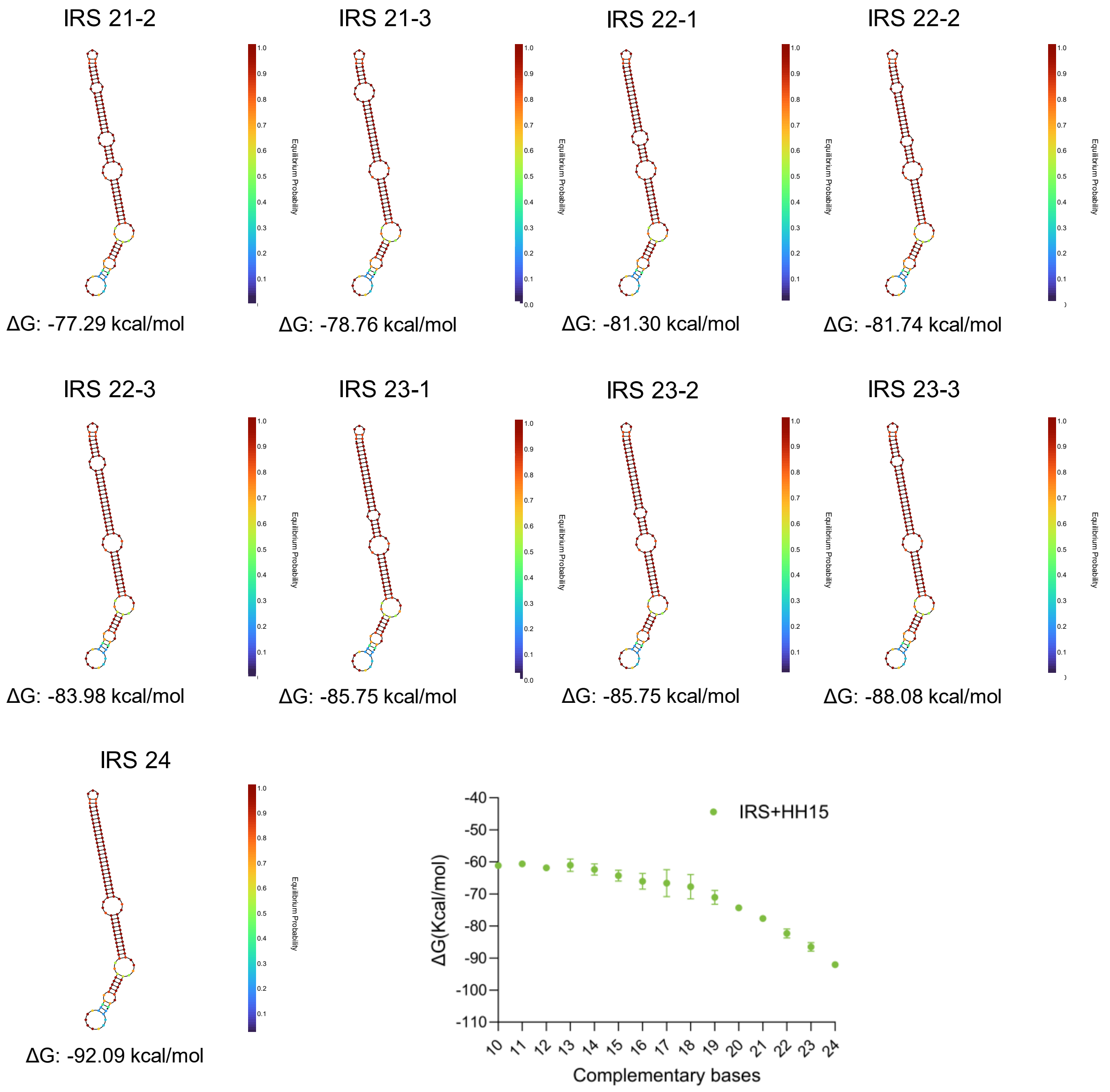

Supplementary Fig.6 | NUPACK-predicted secondary structure and Gibbs free energy between HH15 ribozyme and IRS of different complementary lengths.

The structure was predicted using NUPACK based on the conditions as follows: The nucleic acid type was chosen to be 'RNA'; the salt condition was Na^{+}1.0M and Mg^{2+}0M (the only supported conditions for RNA); the 'dangles' parameter was set to 'all'; the temperature was set to 31 °C (the temperature at which TRACKer assay was performed).

IRS 10

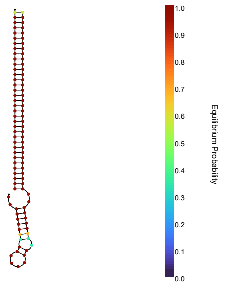

ΔG: -97.35 kcal/mol

IRS 11-1

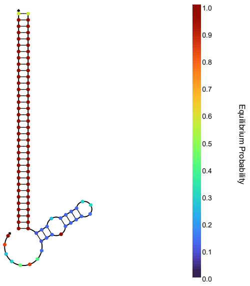

ΔG: -93.15 kcal/mol

IRS 11-2

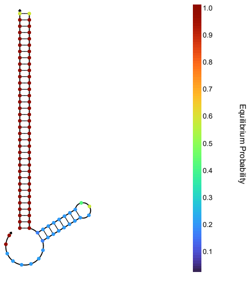

ΔG: -97.39 kcal/mol

IRS 11-3

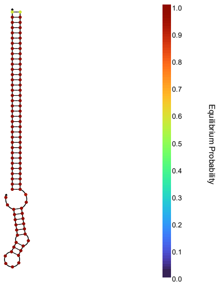

ΔG: -97.35 kcal/mol

IRS 12-1

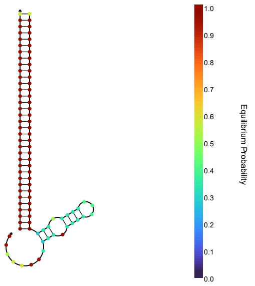

ΔG: -93.15 kcal/mol

IRS 12-2

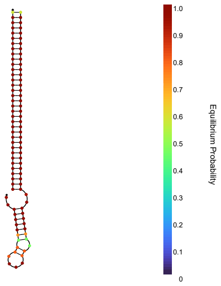

ΔG: -97.52 kcal/mol

IRS 12-3

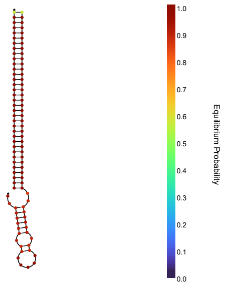

ΔG: -97.78 kcal/mol

IRS 13-1

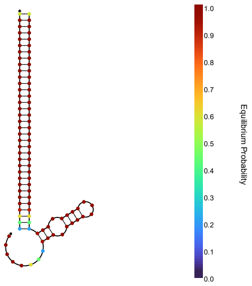

ΔG: -93.13 kcal/mol

IRS 13-2

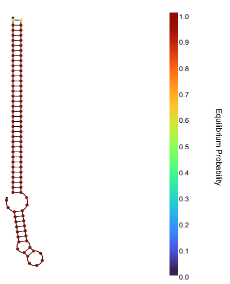

ΔG: -97.52 kcal/mol

IRS 13-3

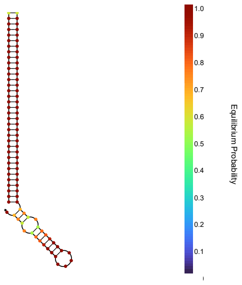

ΔG: -99.40 kcal/mol

IRS 14-1

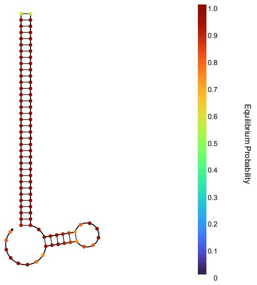

ΔG: -93.34 kcal/mol

IRS 14-2

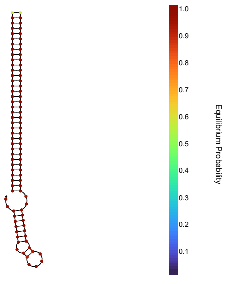

ΔG: -97.52 kcal/mol

IRS 14-3

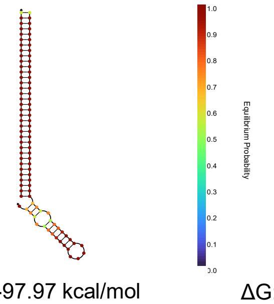

ΔG: -97.97 kcal/mol

IRS 15-1

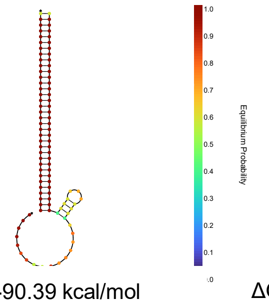

ΔG: -90.39 kcal/mol

IRS 15-2

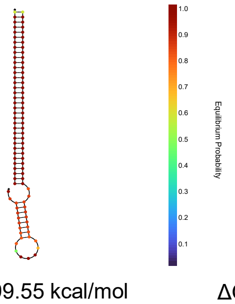

ΔG: -99.55 kcal/mol

IRS 15-3

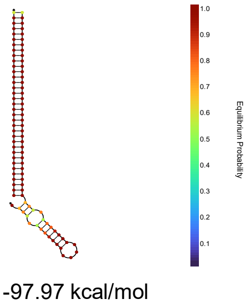

ΔG: -97.97 kcal/mol

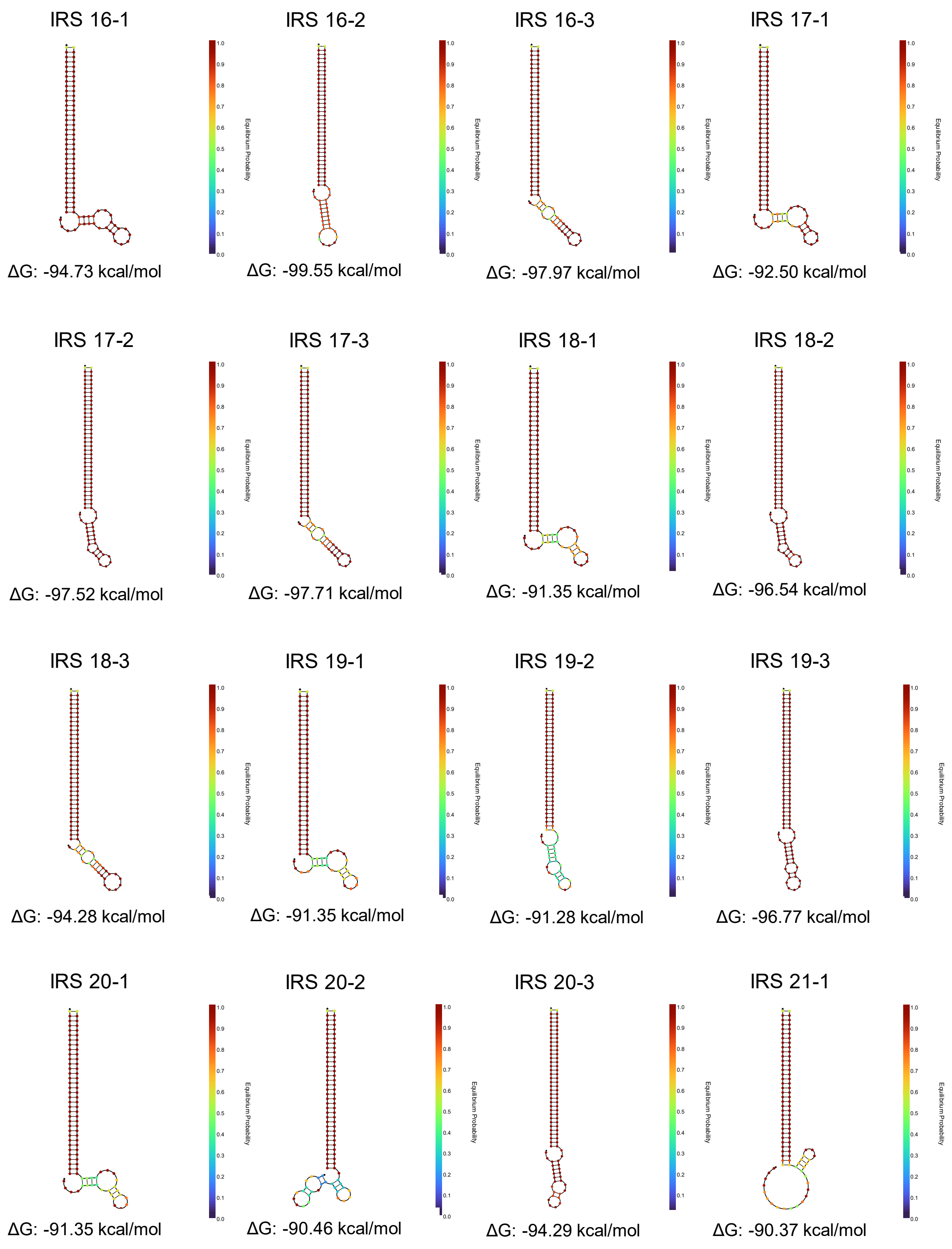
IRS 16-1
ΔG: -94.73 kcal/mol
IRS 16-2
ΔG: -99.55 kcal/mol
IRS 16-3
ΔG: -97.97 kcal/mol
IRS 17-1
ΔG: -92.50 kcal/mol
IRS 17-2
ΔG: -97.52 kcal/mol
IRS 17-3
ΔG: -97.71 kcal/mol
IRS 18-1
ΔG: -91.35 kcal/mol
IRS 18-2
ΔG: -96.54 kcal/mol
IRS 18-3
ΔG: -94.28 kcal/mol
IRS 19-1
ΔG: -91.35 kcal/mol
IRS 19-2
ΔG: -91.28 kcal/mol
IRS 19-3
ΔG: -96.77 kcal/mol
IRS 20-1
ΔG: -91.35 kcal/mol
IRS 20-2
ΔG: -90.46 kcal/mol
IRS 20-3
ΔG: -94.29 kcal/mol
IRS 21-1
ΔG: -90.37 kcal/mol

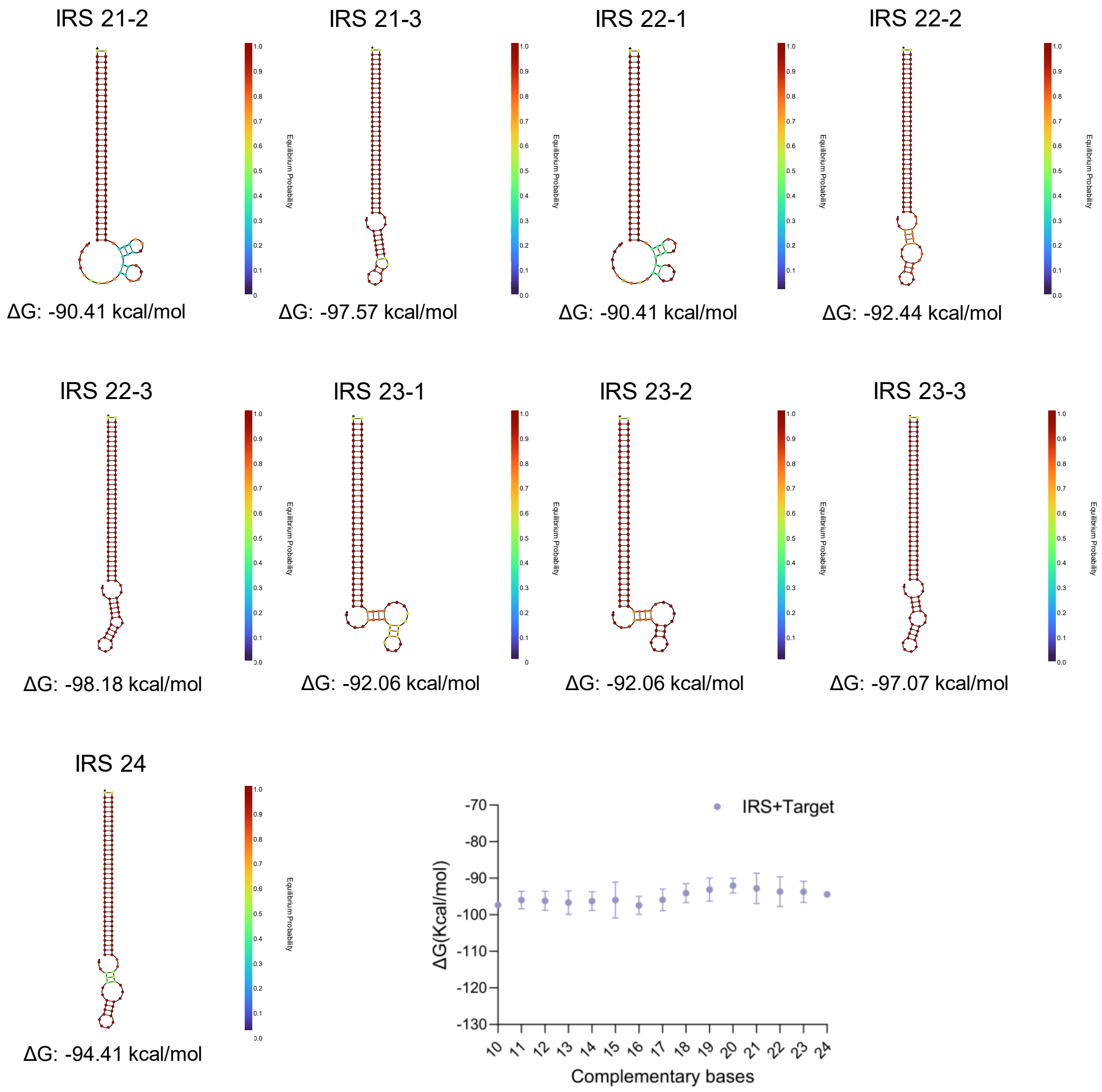

Supplementary Fig.7 | NUPACK-predicted secondary structure and Gibbs free energy between target and IRS of different complementary lengths.

The structure was predicted using NUPACK based on the conditions as follows: The nucleic acid type was chosen to be ‘RNA’; the salt condition was Na^{+}1.0M and Mg^{2+}0M (the only supported conditions for RNA); the ‘dangles’ parameter was set to ‘all’; the temperature was set to 31 °C (the temperature at which TRACKer assay was performed).

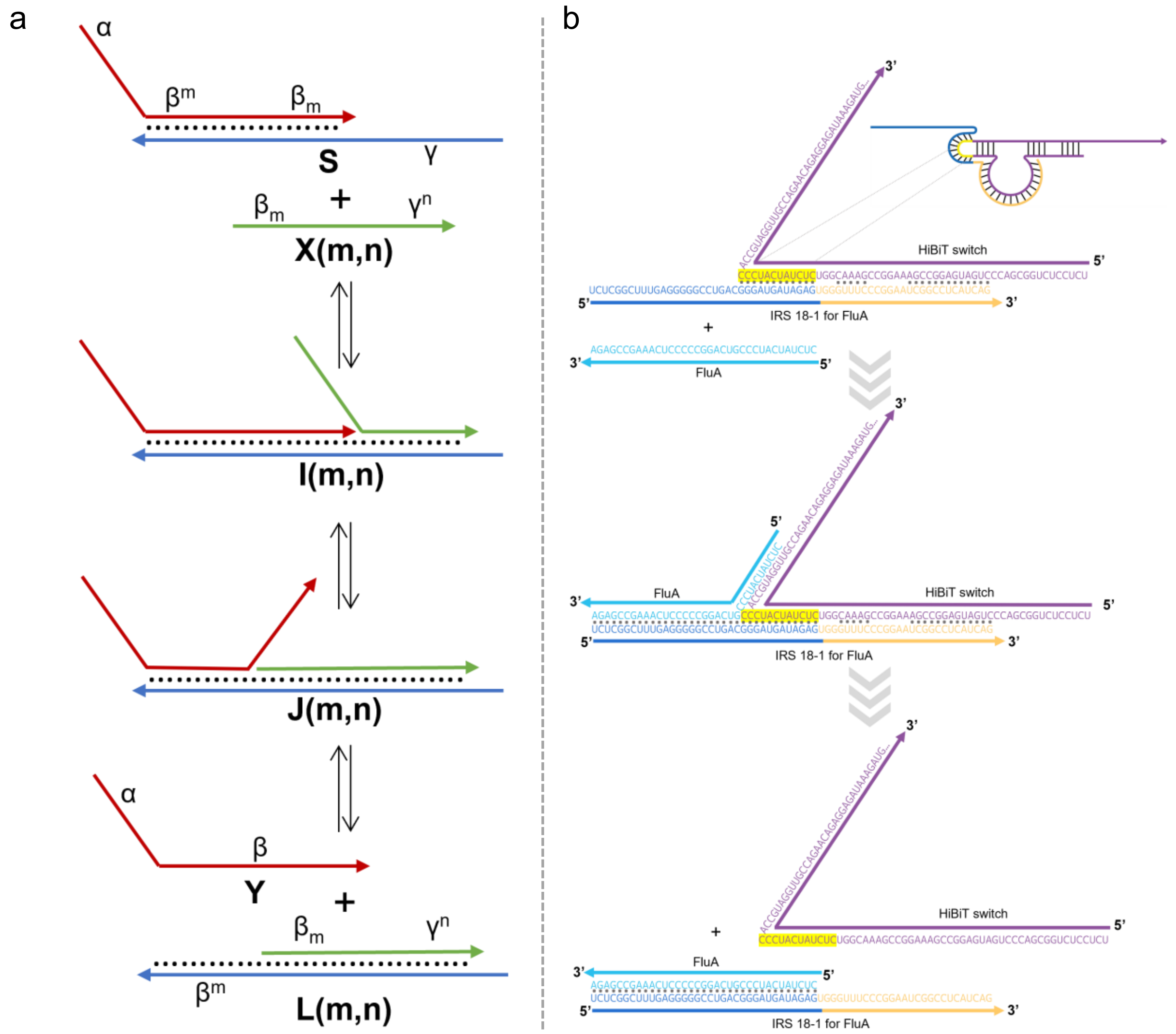

Supplementary Fig. 8 | Strand displacement mechanism of TRACKer based on the principle of toehold exchange. a, Toehold exchange reaction established by *Zhang* and *Winfree*[6]. Invading strand X(m, n) binds to substrate complex S by toehold γ^n (known as the invading toehold) to form intermediate I(m, n). Intermediate I(m, n) represents all branch migration states in which Y is bound to more bases of β_m than X(m, n). Intermediate I(m, n) rearranges to form intermediate J(m, n), which analogously represents all states in which X(m, n) binds more bases of β_m than Y. Domain β^m (known as the incumbent toehold) spontaneously dissociates, releasing products Y and L(m, n). The reaction favors product formation when the length of invading toehold n exceeds that of incumbent toehold m.. **b**, Schematic of the TRACKer strand displacement mechanism. Following the toehold-exchange principle, TRACKer is designed with a 23-nt invading toehold (n = 23) and a 14-nt incumbent toehold (m = 14). The linker between the ribozyme and substrate (yellow region) acts as the branch migration initiation site during competitive strand displacement. With n > m, the reaction favors the forward pathway, yielding the Y strand (HiBiT switch) and L complex (FluA + IRS 18-1).

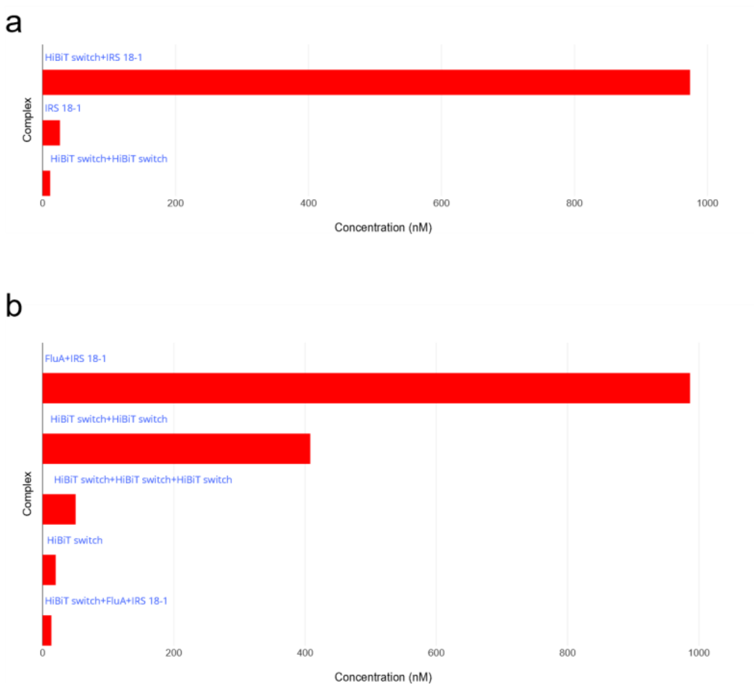

Supplementary Fig. 9 | NUPACK-predicted equilibrium complex concentrations of (a, HiBiT switch + IRS 18-1) and (b, HiBiT switch + IRS 18-1 + FluA). The structure was predicted using NUPACK based on the conditions as follows: The nucleic acid type was chosen to be 'RNA'; the 'dangles' parameter was set to 'all'; the temperature was set to 31 °C (the temperature at which TRACKer assay was performed).

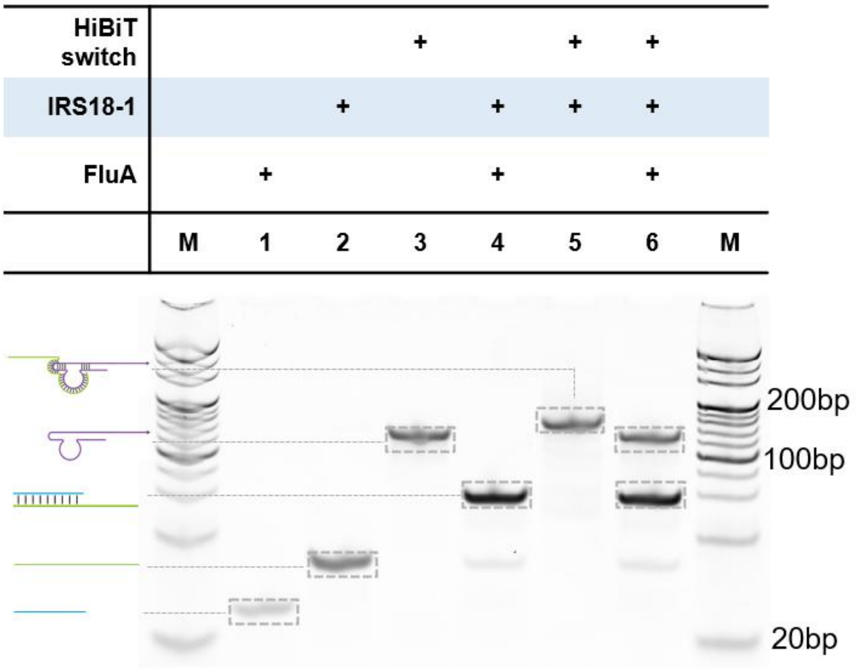

Supplementary Fig. 10 | Native polyacrylamide gel electrophoresis analysis for the strand displacement manchanism.

(Lane 1) FluA

(Lane 2) IRS18-1 that can recognize FluA

(Lane 3) A long strand containing complementary sequences of ribosome- binding sites(RBS*), ribozymes and their substrates, ribosome binding site(RBS), and HiBiT templates, abbreviated as HiBiT switch

(Lane 4) Hybridization of IRS18-1 with FluA

(Lane 5) Hybridization of IRS18-1 with HiBiT switch

(Lane 6) Hybridization of FluA, IRS18-1 and HiBiT switch

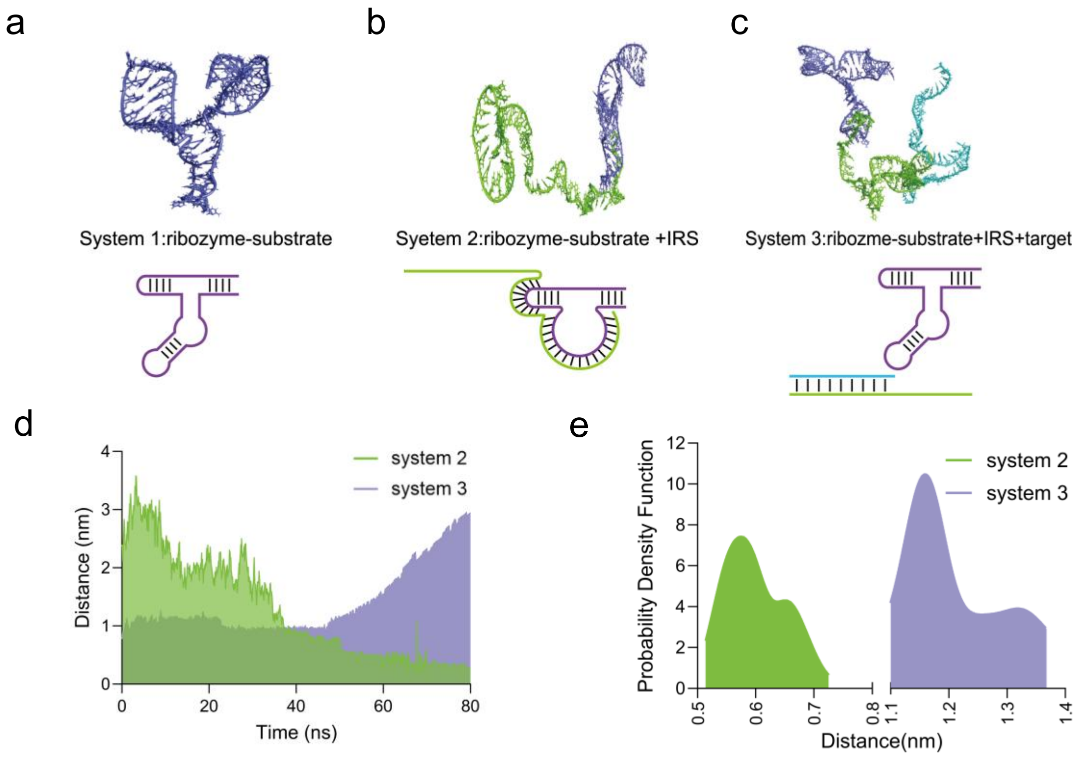

Supplementary Fig. 11 | Mechanistic insights into IRS-mediated ribozyme inhibition and target recognition.

a, Ribozyme-substrate complex (System 1), which adopts the stable secondary structure required for substrate cleavage activity. **b,** Ribozyme-substrate complex in the presence of IRS (System 2): The IRS blocks the ribozyme-substrate interaction, thereby inhibiting cleavage activity. **c,** Ribozyme-substrate complex with both IRS and target RNA (System 3): Target RNA displaces IRS, allowing the ribozyme to undergo structural reformation and regain catalytic activity (catalytic reactivation). **d,**Time-dependent base-pair distance between ribozyme-substrate and IRS in Systems 2 and 3 over the course of 80-nanosecond (ns) molecular dynamics simulations. **e**, Probability density distributions of base-pair distances for Systems 2 and 3. Source data for this figure is available in the Source Data file.

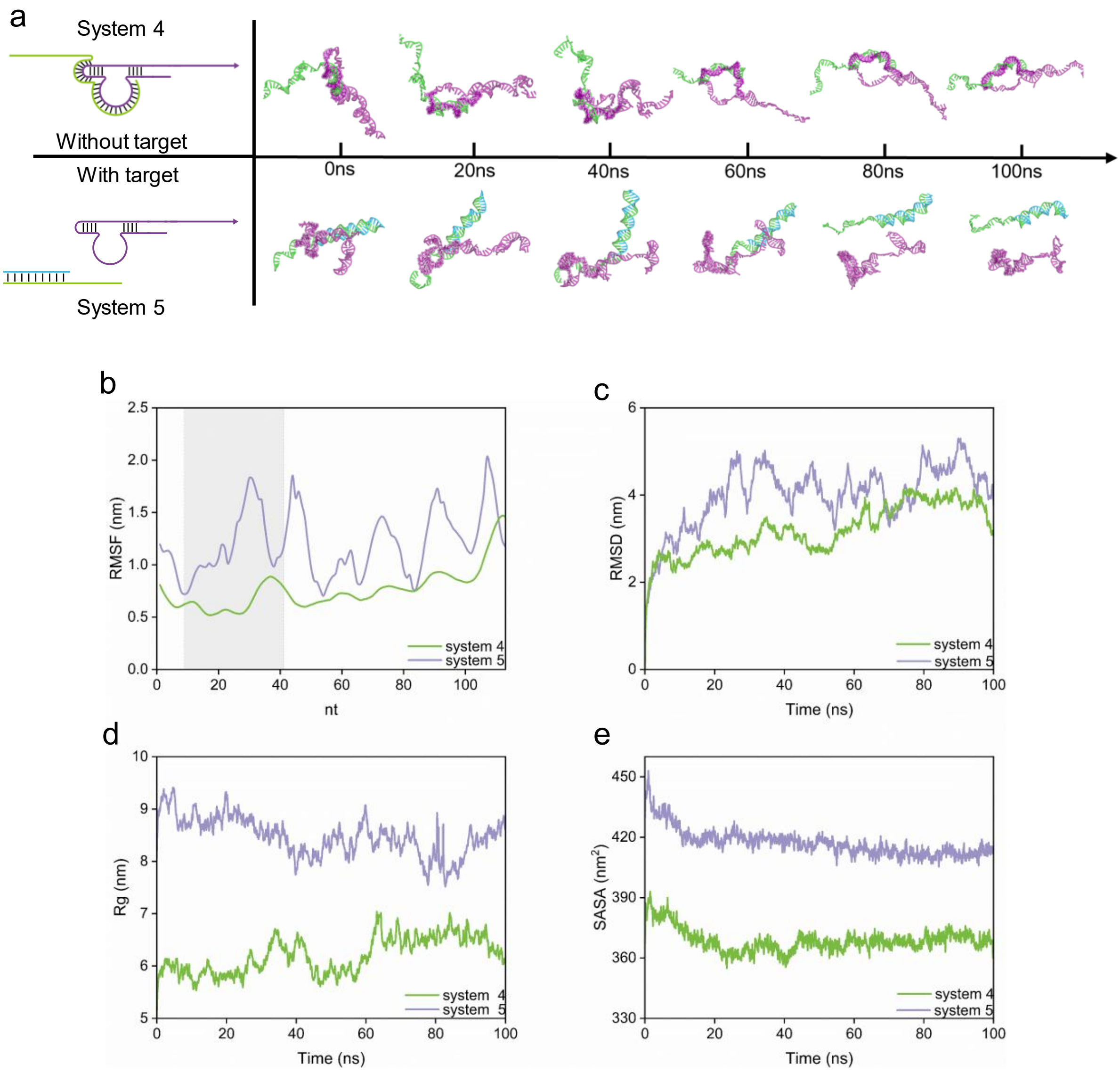

Supplementary Fig. 12 | Mechanistic insights into TRACKer activation.

a, Molecular simulation snapshots of TRACKer at different time points in the absence (System 4) and presence (System 5) of the target. In the absence of the target, the IRS sequence forms a stable conformation with the switch strand; in the presence of the target, the IRS sequence gradually binds to the target and dissociates from the switch strand as the simulation time progresses. **b**, Root mean square fluctuation (RMSF) of the switch strand in Systems 4 and 5. The grey shaded area indicates the region containing the ribozyme sequence. **c-e**, Root mean square deviation (RMSD), radius of gyration (Rg), and solvent-accessible surface area (SASA) of Systems 4 and 5 over the course of the simulation, respectively. Source data for this figure is available in the Source Data file.

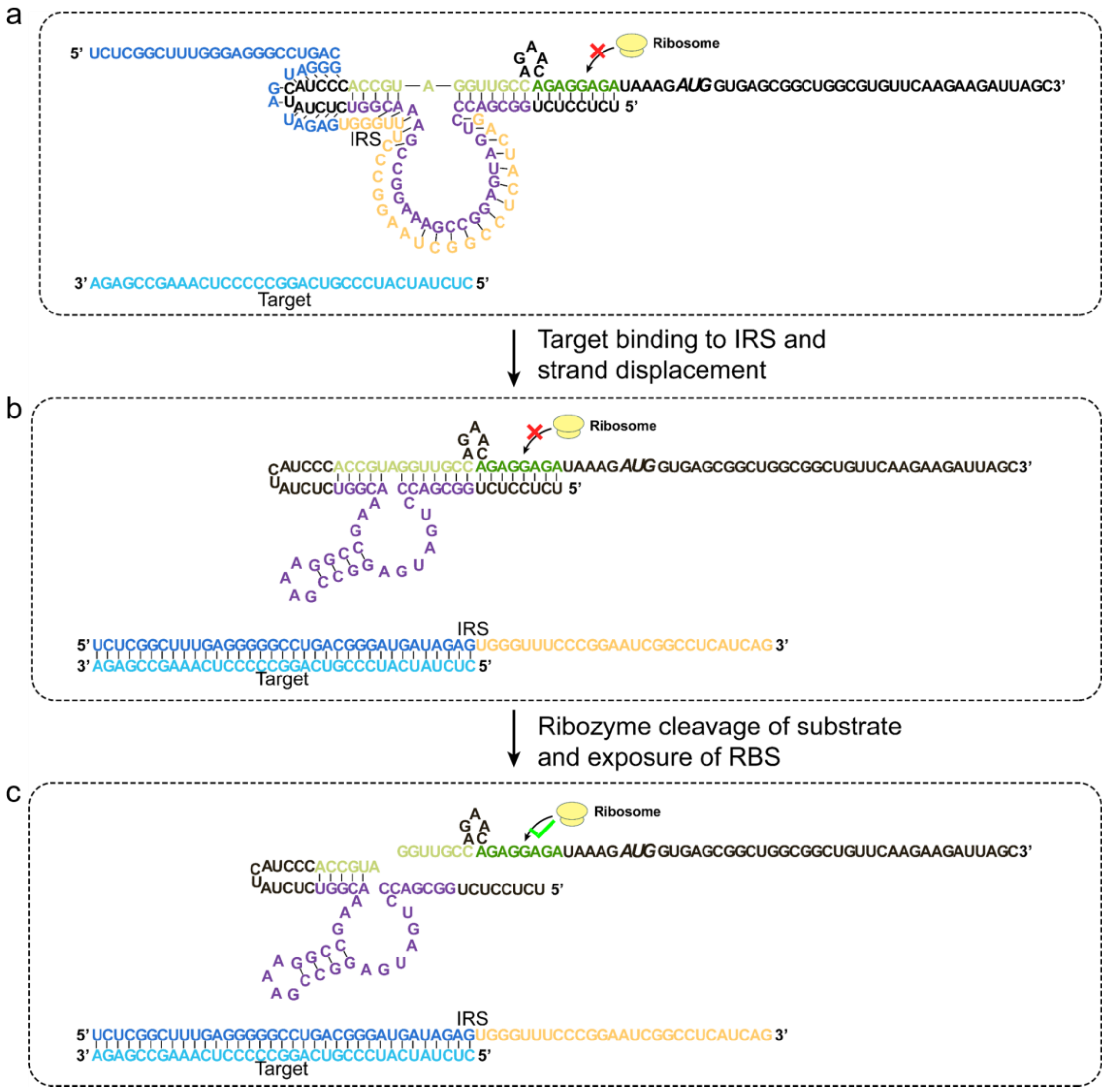

Supplementary Fig. 13 | Schematic diagrams of TRACKer activation.

a-c, Diagrams with sequences illustrating the process of target FluA binding to the IRS 18-1, ribozyme cleavage of substrate, and exposure of RBS, respectively.

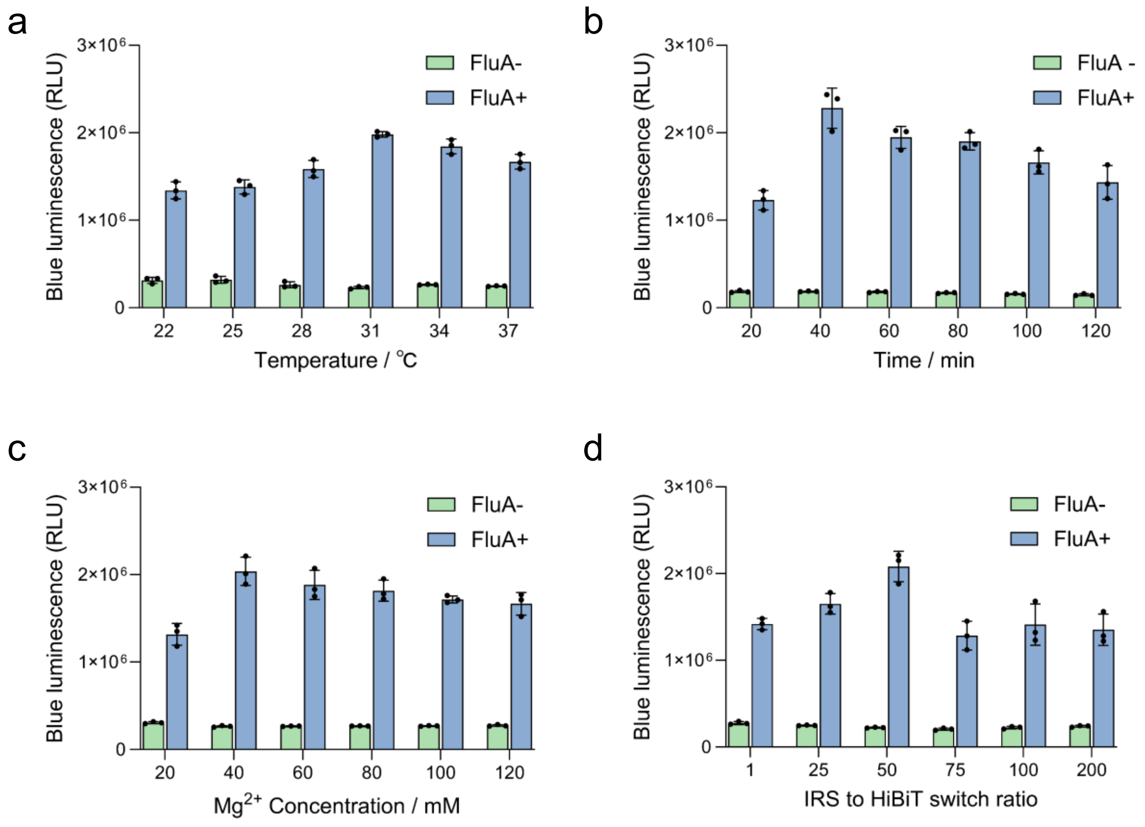

Supplementary Fig. 14 | Optimization of reaction conditions for TRACKer.

a, Optimization of the incubation temperature for the interaction between IRS and the target. **b**, Optimization of the reaction time for TRACKer-based cell-free protein synthesis. **c**, Optimization of magnesium ion concentration in ribozyme-mediated reactions. **d**, Optimization of the concentration ratio between IRS and the HiBiT switch. All experimental measurements are mean ± standard deviation (SD) with n = 3. Source data for this figure is available in the Source Data file.

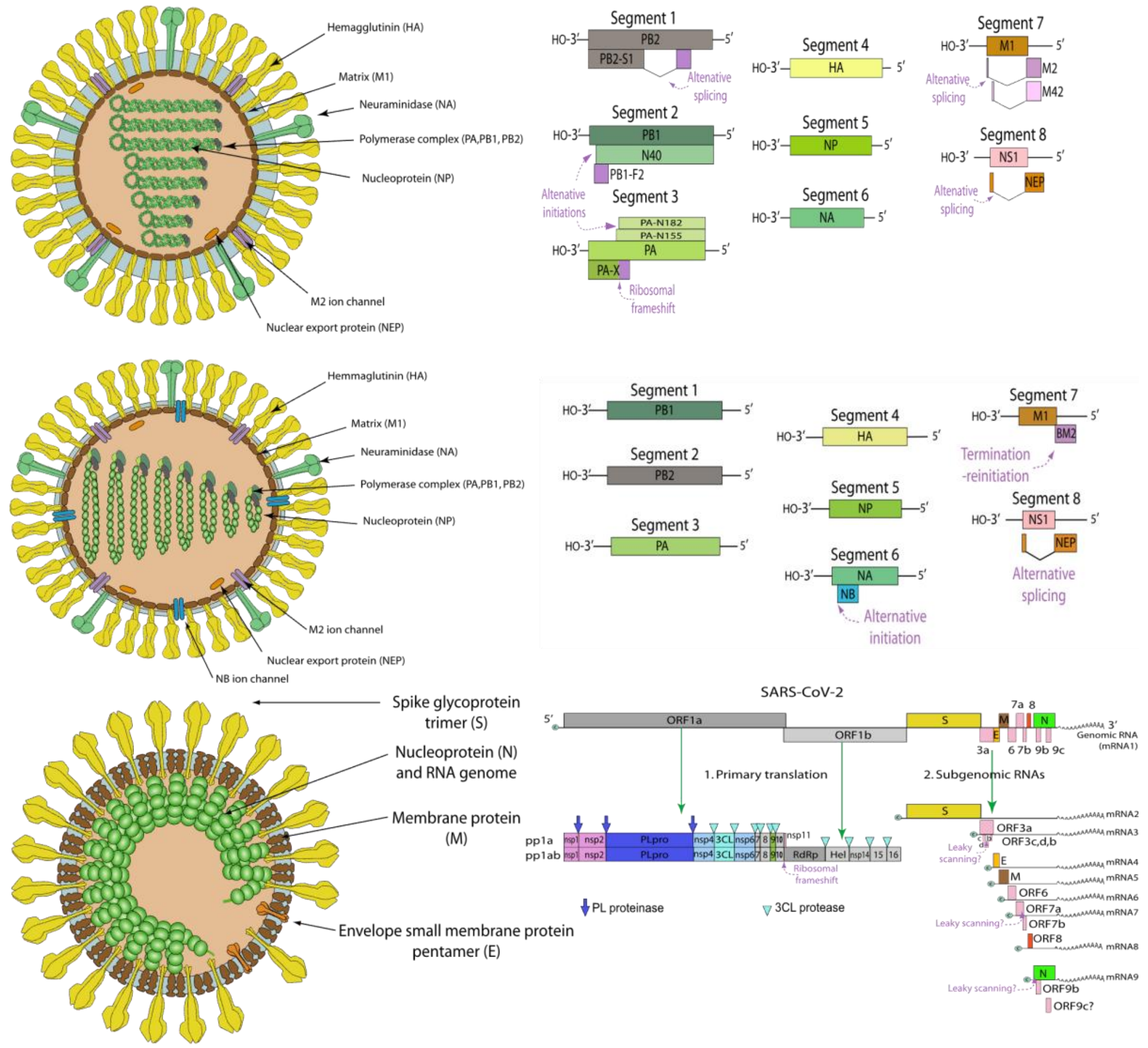
a
Hemagglutinin (HA)
Matrix (M1)
Neuraminidase (NA)
Polymerase complex (PA,PB1,PB2)
Nucleoprotein (NP)
M2 ion channel
Nuclear export protein (NEP)
Segment 1
PB2
PB2-S1
Altenative splicing
Segment 2
PB1
N40
PB1-F2
Altenative initiations
Segment 3
PA-N182
PA-N155
PA
PA-X
Ribosomal frameshift
Segment 4
HA
Segment 5
NP
Segment 6
NA
Segment 7
M1
M2
M42
Altenative splicing
Segment 8
NS1
NEP
Altenative splicing
b
Hemmaglutinin (HA)
Matrix (M1)
Neuraminidase (NA)
Polymerase complex (PA,PB1,PB2)
Nucleoprotein (NP)
M2 ion channel
Nuclear export protein (NEP)
NB ion channel
Segment 1
PB1
Segment 2
PB2
Segment 3
PA
Segment 4
HA
Segment 5
NP
Segment 6
NA
NB
Alternative initiation
Segment 7
M1
BM2
Termination -reinitiation
Segment 8
NS1
NEP
Alternative splicing
c
Spike glycoprotein trimer (S)
Nucleoprotein (N) and RNA genome
Membrane protein (M)
Envelope small membrane protein pentamer (E)
SARS-CoV-2
ORF1a
ORF1b
Genomic RNA (mRNA1)
1. Primary translation
2. Subgenomic RNAs
pp1a
pp1ab
PLpro
RdRp
Hel
Ribosomal frameshift
PL proteinase
3CL protease
ORF3a
ORF3c,d,b
Leaky scanning?
ORF6
ORF7a
ORF7b
ORF8
ORF9b
ORF9c?
mRNA2
mRNA3
mRNA4
mRNA5
mRNA6
mRNA7
mRNA8
mRNA9

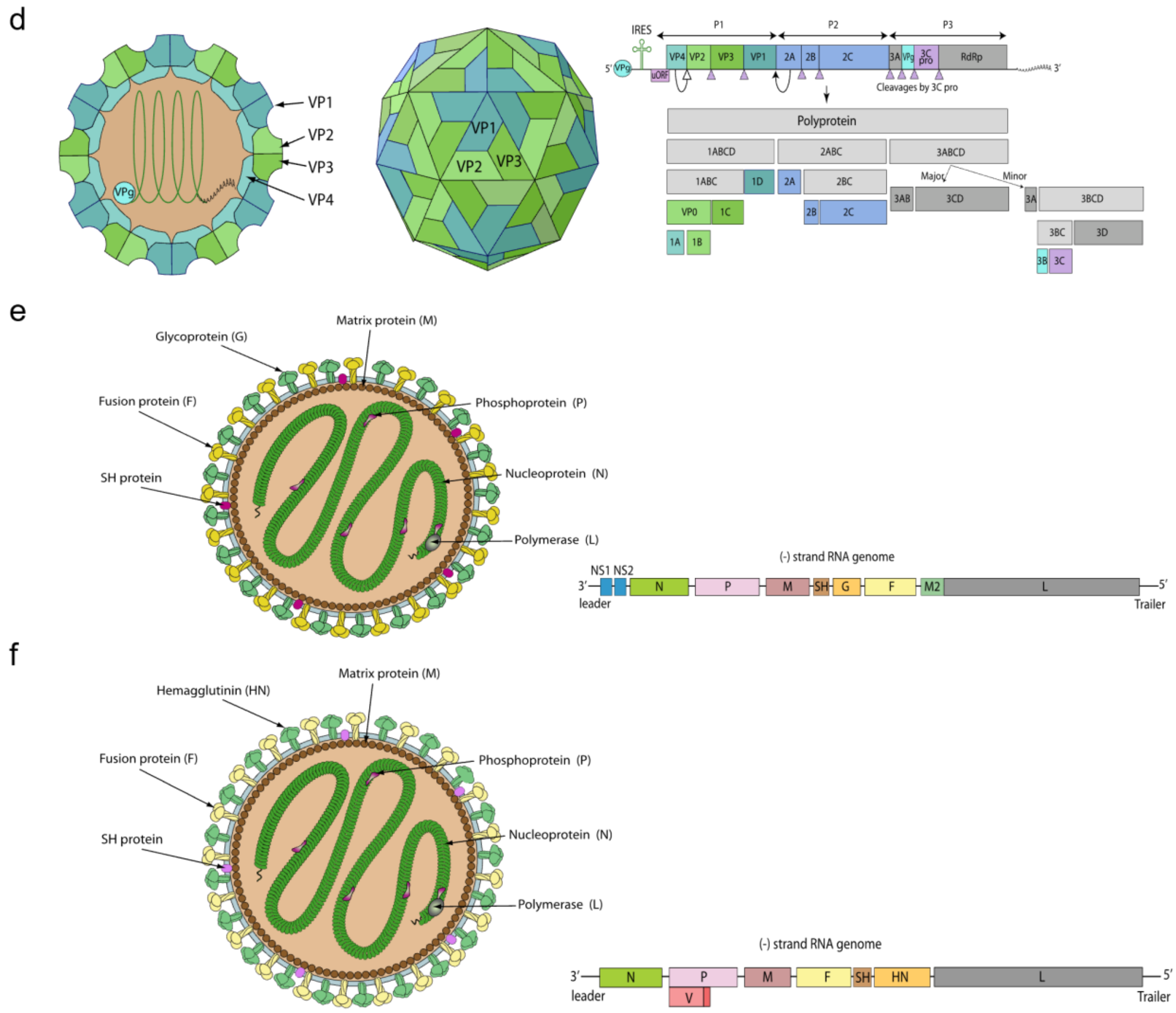

Supplementary Fig. 15 | Virion images of six respiratory virus targets.

a, Influenza A virus (FluA). **b**, Influenza B virus (FluB). **c**, Severe acute respiratory syndrome coronavirus 2 (SARS-CoV-2). **d**, Human rhinovirus (HrV). **e**, Respiratory syncytial virus (RSV). **f**, Human parainfluenza virus (HPIV). All images were adapted from ViralZone[7].

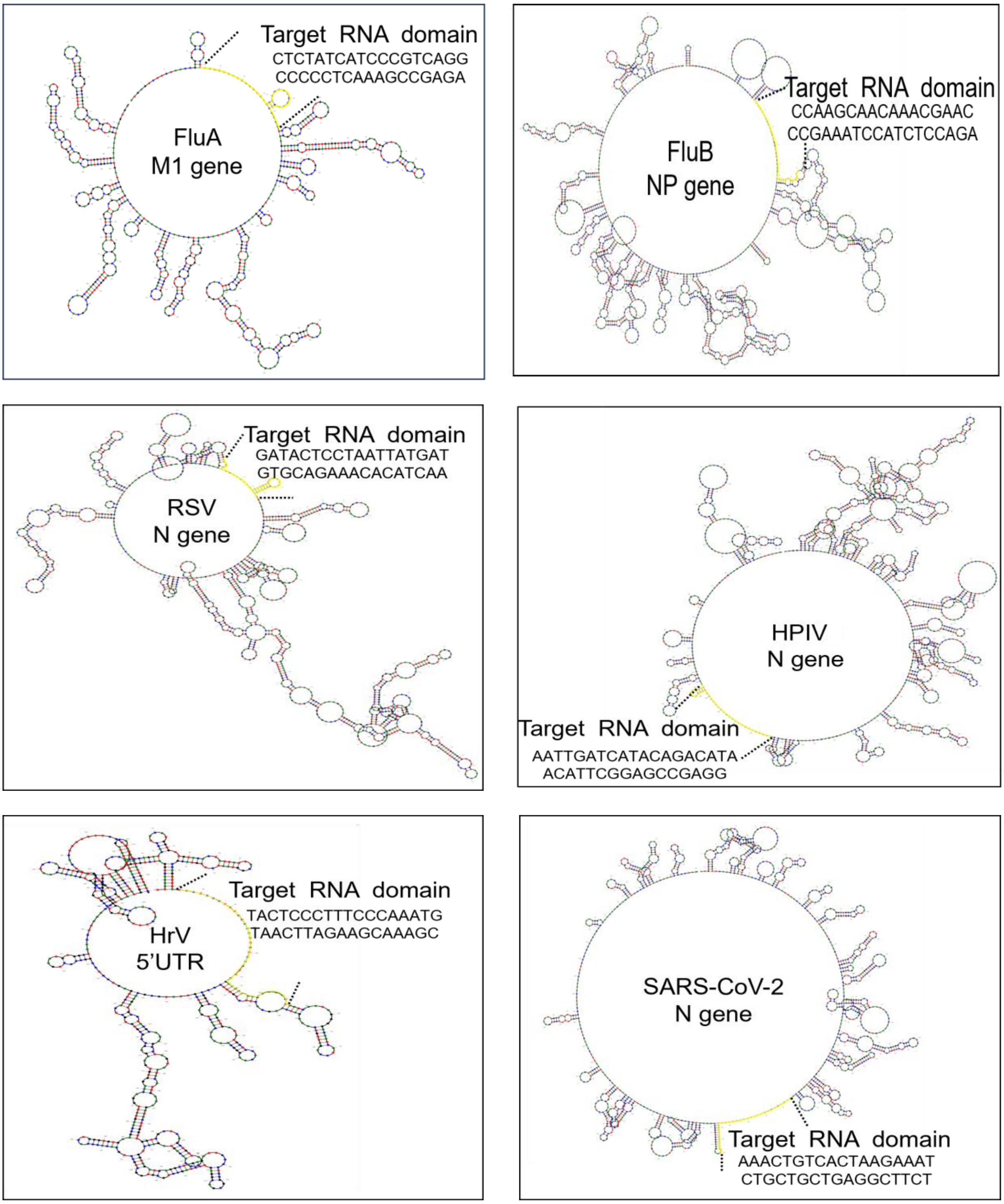

Supplementary Fig. 16 | Secondary structures of viral RNA predicted by NUPACK.

Yellow dots indicate the selected target RNA domains, which are localized to regions lacking complex secondary structures. Structural predictions via NUPACK were performed under the following conditions: nucleic acid type set to "RNA"; "dangles" parameter set to "none"; and temperature set to 31°C (the optimal temperature for co-incubation of IRS and the target).

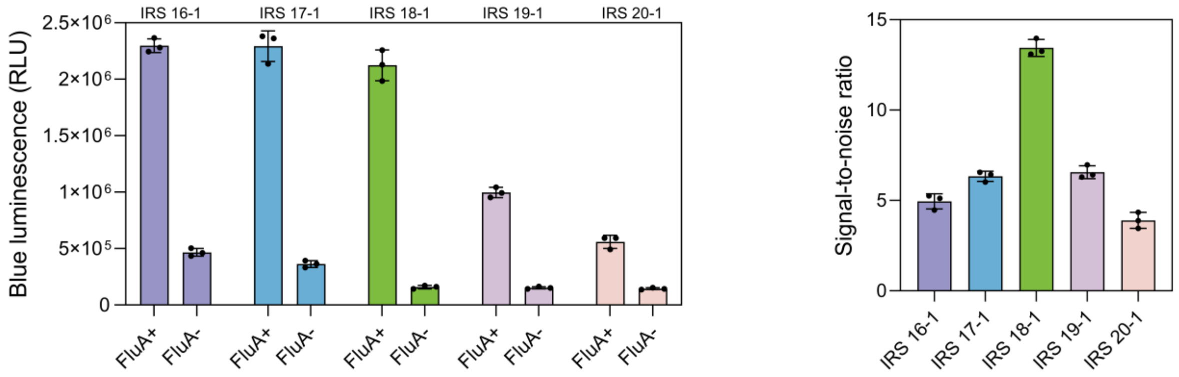

Supplementary Fig. 17 | a, Endpoint luminescence of TRACKer for FluA detection with IRS16-1, 17-1, 18-1, 19-1, and 20-1. b, Signal-to-noise ratio of TRACKer for FluA detection with IRS16-1, 17-1, 18-1, 19-1, and 20-1.

Blue luminescence was measured after cell-free protein synthesis reaction completion and furimazine addition. All experimental measurements are mean ± standard deviation (SD), n = 3. Source data for this figure is available in the Source Data file.

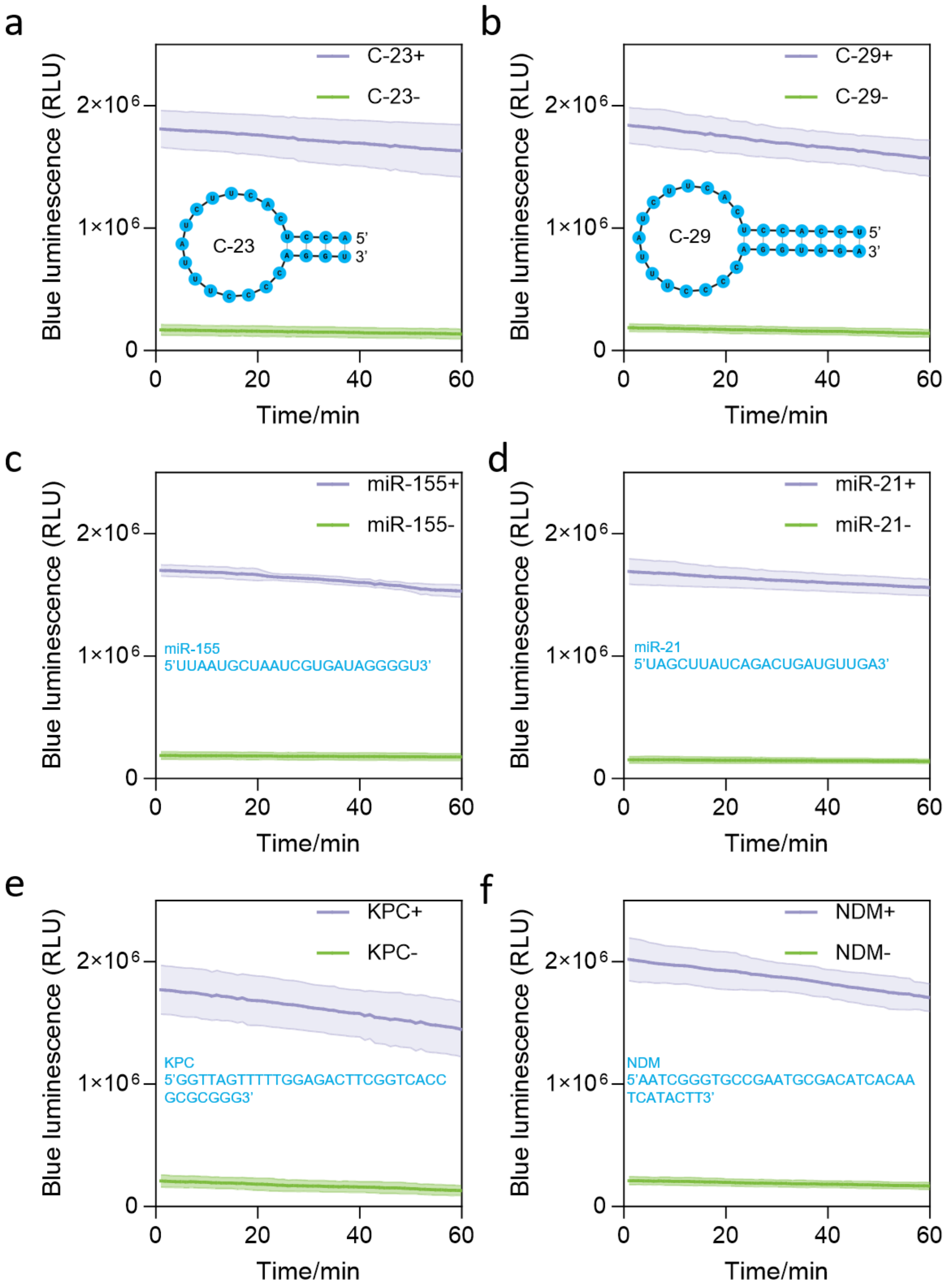

Supplementary Fig. 18 | Time-course luminescence measurements of TRACKer for detection of six different target: C-23(a), C-29(b), miR-155(C), miR-21(d), KPC(e), and NDM(f).

Blue luminescence was measured continuously for 60 minutes following the completion of the cell-free protein synthesis reaction and the addition of furimazine. Shaded regions represent mean ± standard deviation (SD), with n = 3 technical replicates. Source data for this figure is available in the Source Data file.

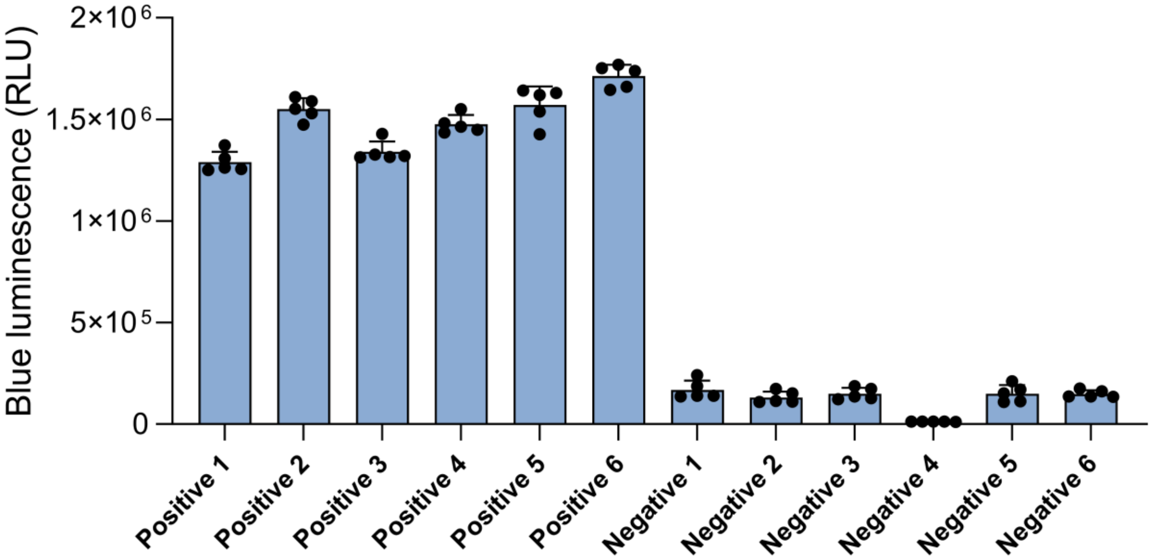

Supplementary Fig.19 | Detection capability of TRACKer for pseudovirus harboring the FluA M1 gene in a laboratory setting.

The pseudovirus used in this study was acquired from Shanghai Shenggong Biological Engineering Technology & Services Co., Ltd. Data are shown as mean ± standard deviation (SD), with n = 5 technical replicates. Source data for this figure is available in the Source Data file.

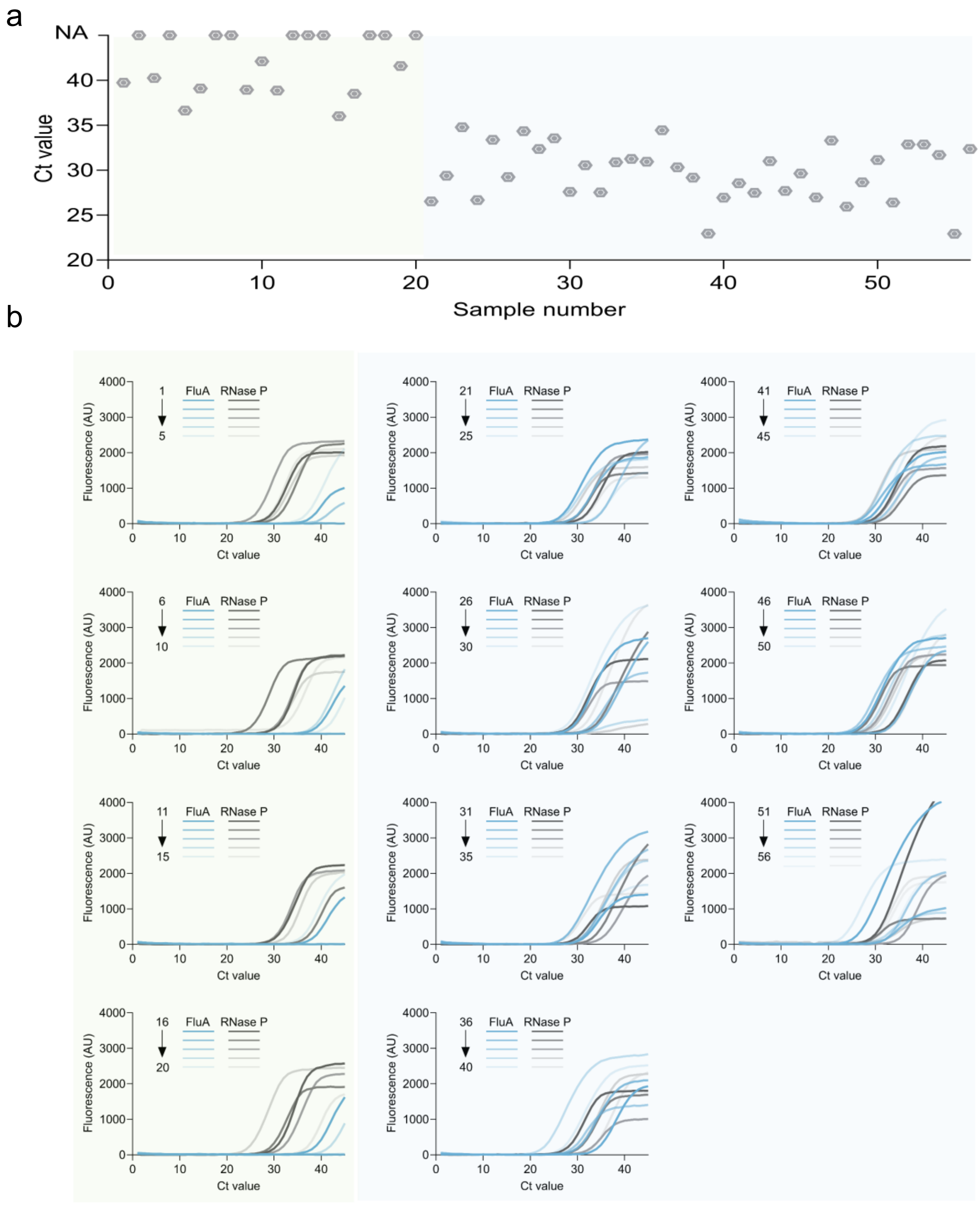

Supplementary Fig.20 | RT-qPCR detection of influenza A (FluA) virus in 56 clinical pharynx swab samples.

a, Cycle threshold (Ct) values of 56 clinical pharyngeal swab samples for FluA targeting. **b**, FluA amplification curves derived from 56 clinical pharyngeal swab samples. “NA” in panel a indicates the absence of a detectable Ct value. Ribonuclease P (RNase P) was used as an internal reference gene to ensure the reliability of the experimental results. Source data for this figure is available in the Source Data file.

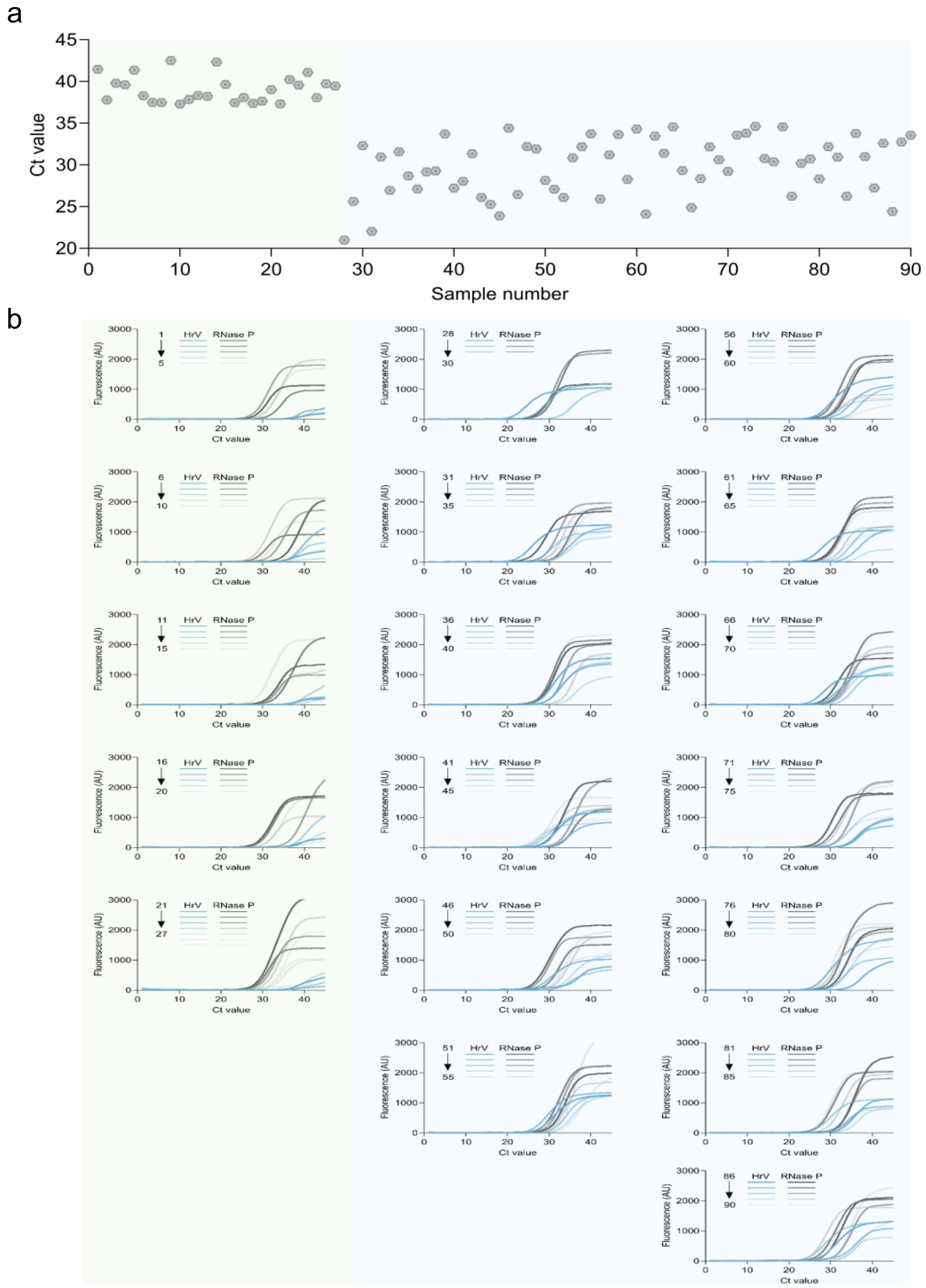

Supplementary Fig.21 | RT-qPCR detection of Human rhinovirus (HrV) in 90 clinical pharynx swab samples.

a, Cycle threshold (Ct) values of 90 clinical pharyngeal swab samples for HrV targeting. **b**, HrV amplification curves derived from 90 clinical pharyngeal swab samples. "NA" in panel a indicates the absence of a detectable Ct value. Ribonuclease P (RNase P) was used as an internal reference gene to ensure the reliability of the experimental results. Source data for this figure is available in the Source Data file.

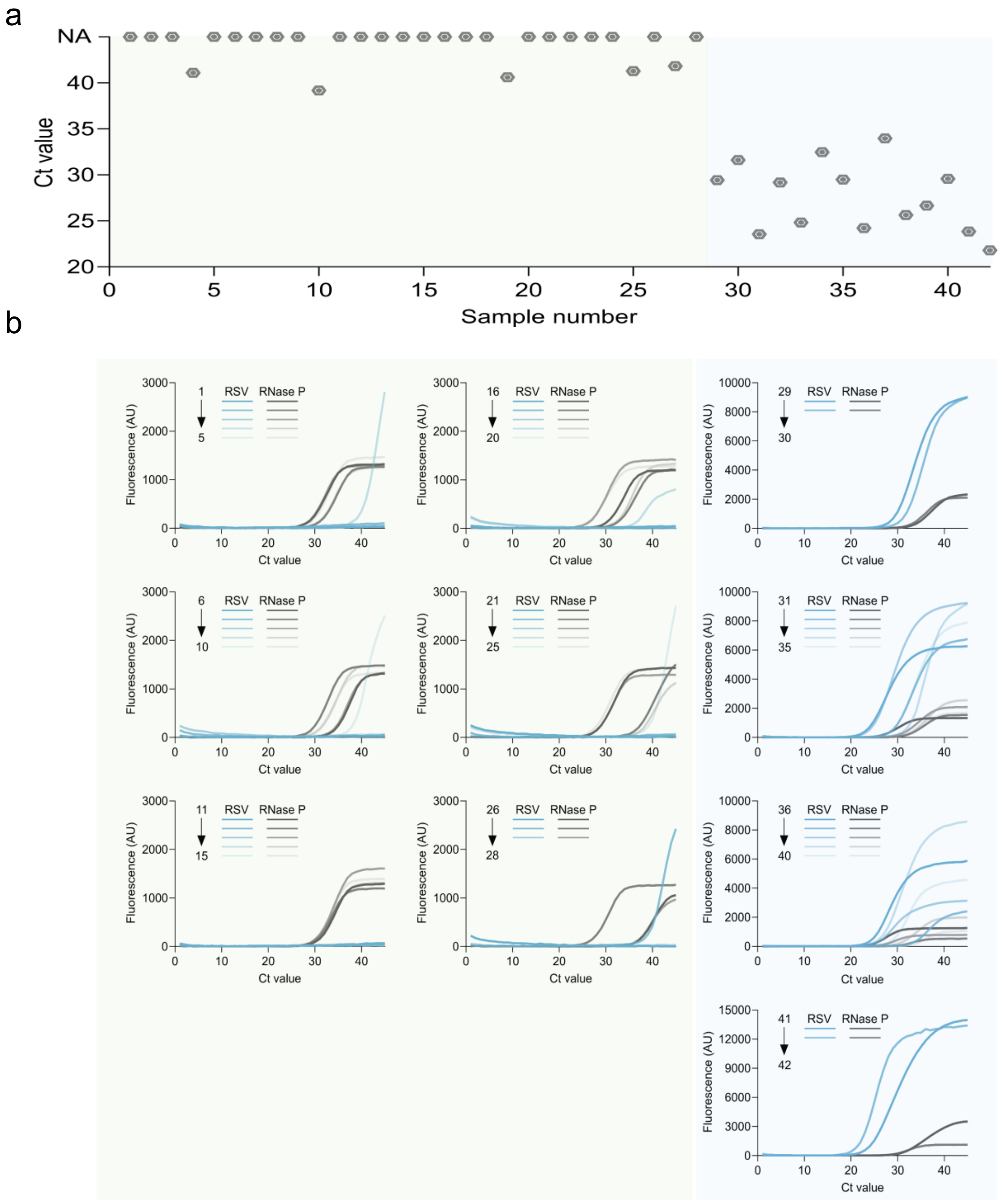

Supplementary Fig. 22 | RT-qPCR detection of respiratory syncytial virus (RSV) in 42 clinical pharynx swab samples.

a, Cycle threshold (Ct) values for respiratory syncytial virus (RSV) in 42 clinical pharyngeal swab samples. **b**, RSV amplification curves derived from 42 clinical pharyngeal swab samples. "NA" in panel a indicates the absence of a detectable Ct value. Ribonuclease P (RNase P) was used as an internal reference gene to ensure the reliability of the experimental results. Source data for this figure is available in the Source Data file.

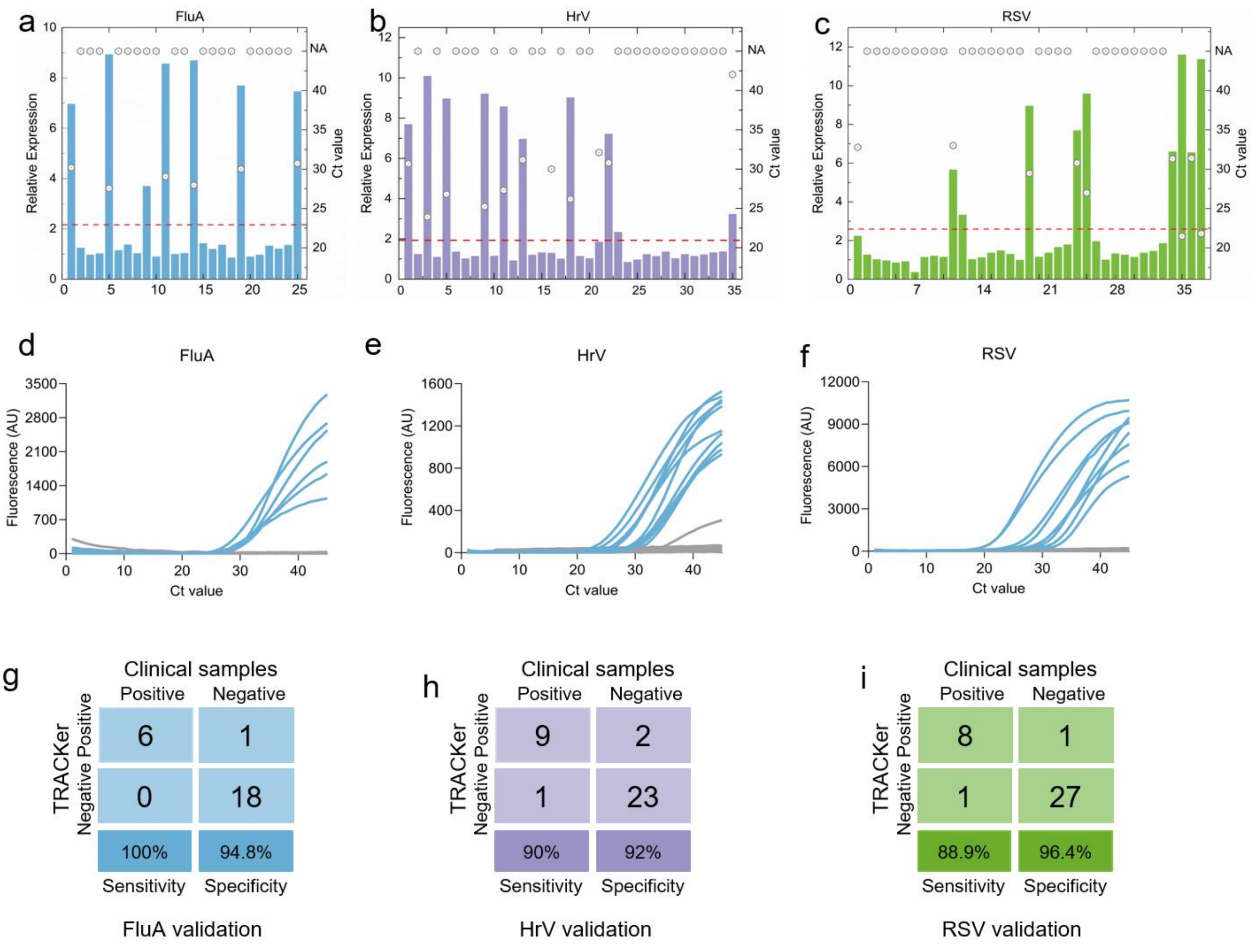

Supplementary Fig. 23 | Clinical validation of TRACKer's diagnostic performance.

a-c, Comparative analysis of FluA (**a**), HrV (**b**), and RSV (**c**) between RT-qPCR and TRACKer assays. Histograms depict relative expression levels, defined as the ratio of RLUgene to RLUnegative (RLUgene and RLUnegative denote RLU values for target viral genes and negative controls, respectively). Gray hexagons represent RT-qPCR cycle threshold (Ct) values. Red dashed lines indicate relative expression cut-offs determined by ROC curve analysis in Fig. 5f. **d-f**, Fluorescence curves of FluA (**d**), HrV (**e**), and RSV (**f**). **g-i**, Clinical sensitivity and specificity of TRACKer for detecting FluA (**g**), HrV (**h**), and RSV (**i**), assessed against clinical screening tests via a confusion matrix. NA in a-c indicates no ct value. Source data for this figure is available in the Source Data file.

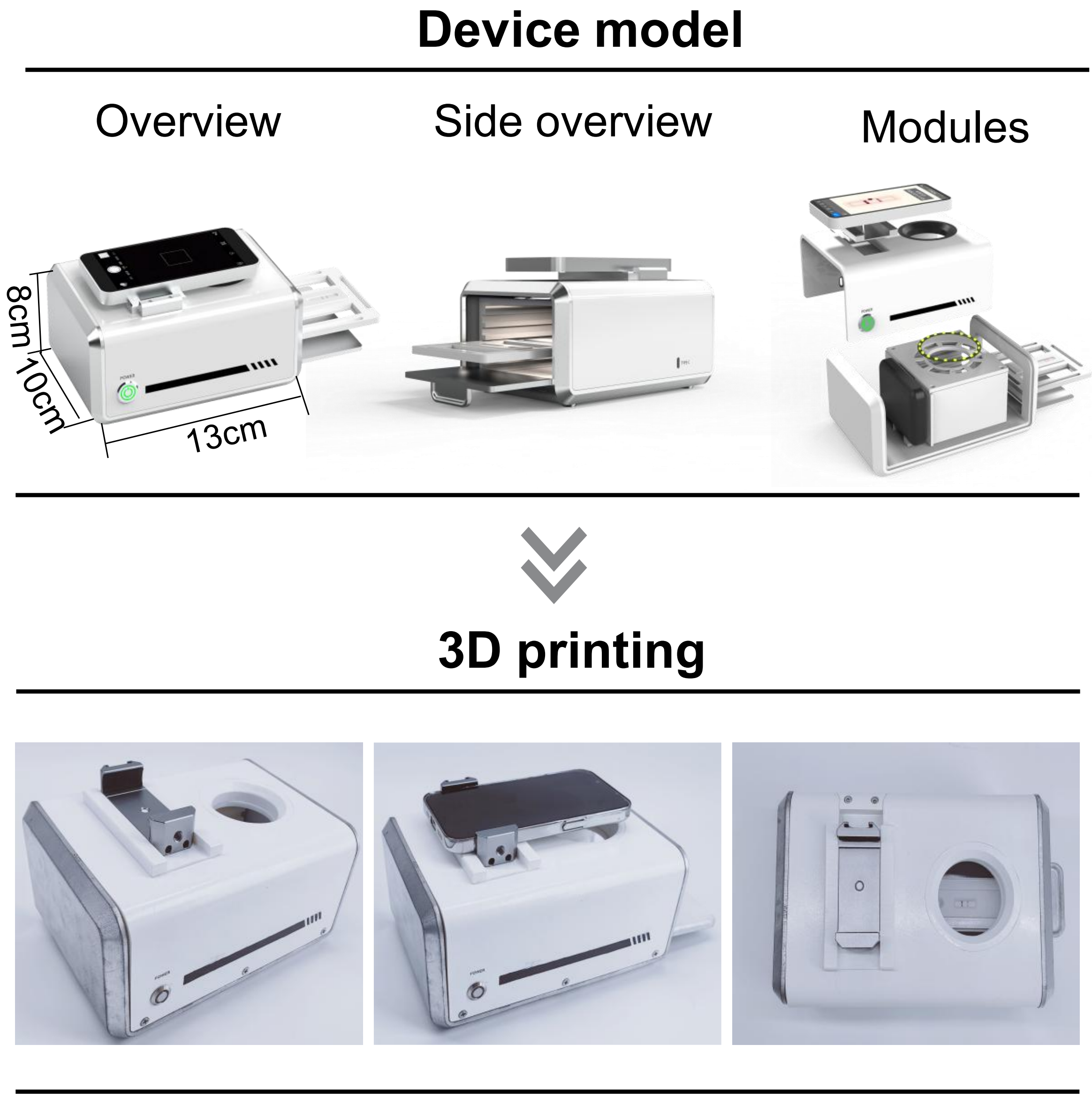

Supplementary Fig. 24 | Attachment device.

The attachment device consists of three components: a darkroom enclosed by a shell, an adjustable phone holder, and a light-emitting diode (LED) controlled by a touch switch. This LED functions as a uniform, stable light source for lateral flow assay (LFA) imaging using a smartphone. The device has dimensions of 13 cm (length) × 10 cm (width) × 6 cm (height) and a weight of approximately 0.1 kg. The device was modeled with Rhino 6 software and manufactured by Lite 800 (Union Tech., Shanghai, China).

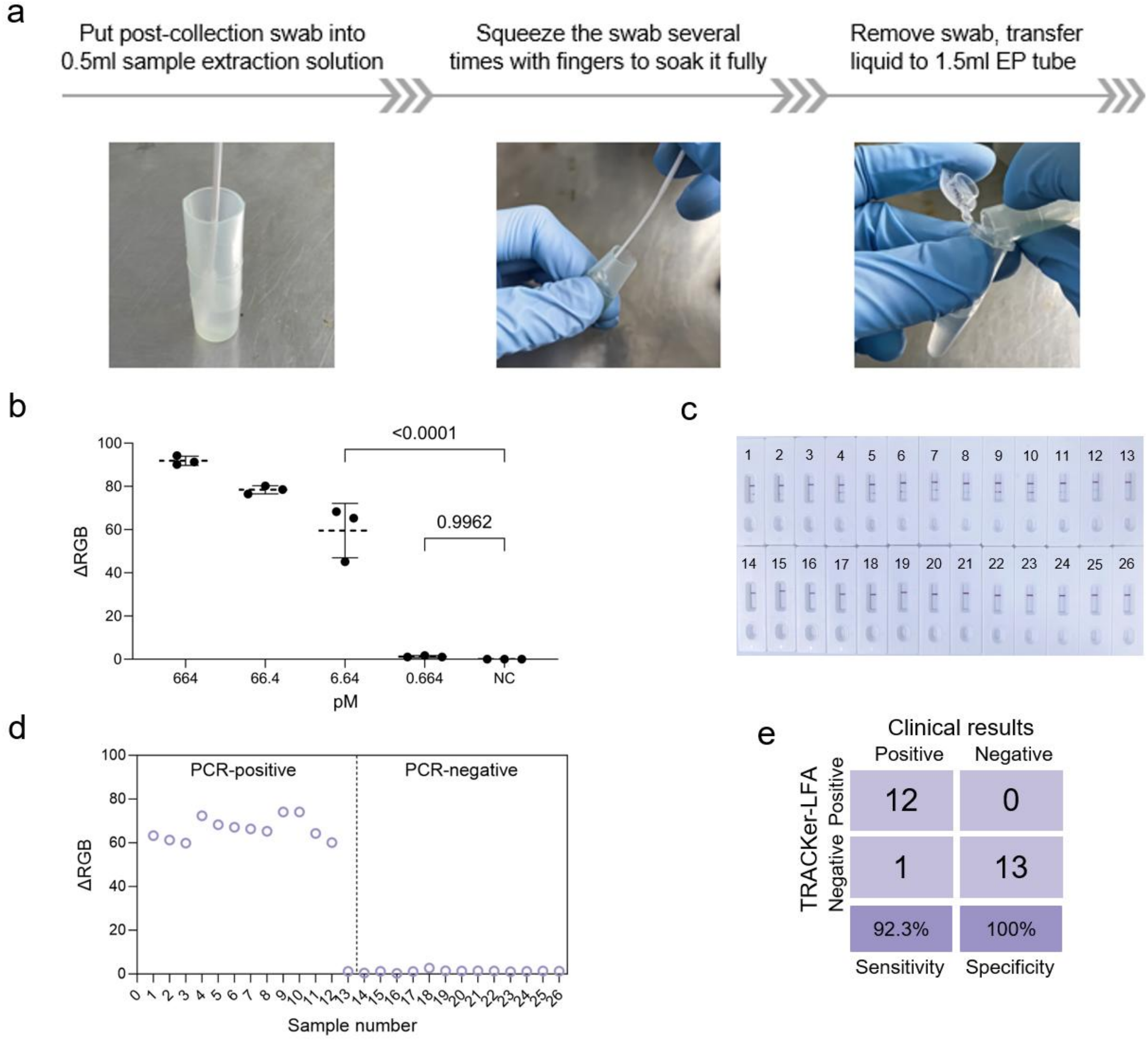

Supplementary Fig.25 | Diagnostic performance of TRACKer-LFA with simplified sample preparation.

a, Schematic of nucleic acid extraction from swabs of suspected cases using sample extraction solution (main components: 10 mM Tris + 0.1% Tween-20). **b**, Limit of detection (LOD) of TRACKer-LFA for FluA pseudoviruses following simplified extraction. Data are shown as mean ± standard deviation (SD) with three technical replicates. **c**, Visual detection of 13 FluA-positive and 13 FluA-negative clinical samples via TRACKer-LFA with simplified extraction. **d**, ΔRGB values from testing 26 clinical FluA swab samples. **e**, Clinical sensitivity and specificity of TRACKer-LFA, assessed against clinical screening tests using a confusion matrix (n = 13 for negative samples and n = 13 for positive samples). Source data for this figure is available in the Source Data file.

Supplementary Table. 1 | Free Energy Analysis for systems 1, 2, and 3.

	ΔVDWAALS	ΔEEL	ΔEGB	ΔESURF	ΔTOTAL
system1	-87.56	490.44	-488.32	-8.96	-94.4
system2	-67.03	465.39	-465.61	-7.54	-74.78
system3	-78.33	477.18	-477.42	-8.02	-86.6

Supplementary Table. 2 | Clinical information of cohorts used for detecting influenza A (FluA), respiratory syncytial virus (RSV), and human rhinovirus (HrV).

	**Non-infected individuals**	**FluA infected individuals**	**Total**
Number	20	36	56
Age			
Median	30	58	49
Range	1-83	1-92	1-92
Sex			
Male	11	20	31
Female	9	16	25
	**Non-infected individuals**	**RSV infected individuals**	**Total**
Number	28	14	42
Age			
Median	7	21	49
Range	1-77	3-74	1-77
Sex			
Male	12	5	17
Female	16	9	25
	**Non-infected individuals**	**HrV infected individuals**	**Total**
Number	27	63	90
Age			
Median	26	21	23
Range	1-73	1-91	1-91
Sex			
Male	14	20	34
Female	13	43	56

Supplementary Table. 3 | Predictive agreement between TRACKer and RT-qPCR for clinical samples.

Clinical samples				
**FluA**				
	Number		Number	Predictive agreement with RT-qPCR, %
RT-qPCR Positive	36	TRACKer Positive	34	94.44
		TRACKer Negative	2	
RT-qPCR Negative	20	TRACKer Positive	0	100
		TRACKer Negative	20	
**RSV**				
	Number		Number	Predictive agreement with RT-qPCR, %
RT-qPCR Positive	14	TRACKer Positive	14	100
		TRACKer Negative	0	
RT-qPCR Negative	28	TRACKer Positive	0	100
		TRACKer Negative	28	
**HrV**				
	Number		Number	Predictive agreement with RT-qPCR, %
RT-qPCR Positive	63	TRACKer Positive	59	93.65
		TRACKer Negative	4	
RT-qPCR Negative	27	TRACKer Positive	0	100
		TRACKer Negative	27	

Supplementary Table. 4 | Comparison of TRACKer with other cell-free detection methods.

Method	Target	Report protein	Sensitivity	Time-to-result	Pre-amplification	Supplementary reference
Toehold switch (NASBA-CRISPR enhanced)	Zika virus	LacZ	2.8fM	3h	Need (NASBA)	8
Toehold switch (NASBA enhanced)	RSV	LacZ	91aM	2.5h	Need (NASBA)	9
Toehold switch (NASBA enhanced)	SARS-CoV-2	GFP	1800 copies	5h	Need (NASBA)	10
Toehold switch (NASBA enhanced)	Gut bacteria	GFP	30aM	3-5h	Need (NASBA)	11
Toehold switch	SARS-CoV-2	Nanoluc	10 nM	1h30min	not need	12
Synthetic metabolic cascades cell-free biosensors	Benzoic acid, Hippuric acid, Cocaine	sfGFP	1μM	4h	not need	13
INSPECTR	SARS-CoV-2	Dual-epitope peptide	3.5fM	5h	Need (RCA)	14
TRACKer	FluA,FluB, RSV, HPIV, HrV, SARS-CoV-2	HiBiT/Flag-His peptide	1-10aM	70min	not need	This work

Supplementary Table. 5 | Estimated cost per TRACKer reaction.

Component	Supplier	Catalog Number	Price (USD)	Stock	Stock Volume (mL)	Amount/Reaction (mL)	Reactions	Cost / Reaction(USD)
**Laboratory mode**								
RNA template[a]	n/a	n/a	28.08	50μM	0.12	0.0008	140	0.2
$MgCl_2$	Byotime	ST269	5	1M	100	0.004	25000	<0.01
Cell-Free Expression Premix (CFPS kit)	Suzhou Perotin	HTE01	980	1344 reaction	50	0.0372	1344	0.72
LgBiT + Furimazine	Promega	N3030	135	200 reaction	10	0.05	200	0.67
Labor cost	n/a	n/a	n/a	n/a	n/a	n/a	n/a	0.23
Storage cost	n/a	n/a	n/a	n/a	n/a	n/a	n/a	0.1
Transportation cost	n/a	n/a	n/a	n/a	n/a	n/a	n/a	0.05
Total								1.98
**Onsite mode**								
RNA template[a]	n/a	n/a	28.08	50μM	0.12	0.0008	140	0.2
$MgCl_2$	Byotime	ST269	5	1M	100	0.004	25000	<0.01
Cell-Free Expression Premix (CFPS kit)	Suzhou Perotin	HTE01	980	1344 reaction	50	0.0372	1344	0.72
LFA test strip	Biodragon	BF06219	150	200strips	n/a	1 strip / reaction	200	0.75
LED device	n/a	n/a	50	n/a	n/a	n/a	10000	<0.01
Labor cost	n/a	n/a	n/a	n/a	n/a	n/a	n/a	0.23
Storage cost	n/a	n/a	n/a	n/a	n/a	n/a	n/a	0.1
Transportation cost	n/a	n/a	n/a	n/a	n/a	n/a	n/a	0.05
Total								2.07

[a]Calculated the costs of 0.1 mL in vitro transcription products and purification, using BBI #B639253-0100 ($2.7 per preparation), NEB #T1130S ($24.38 per preparation), and DNA template (< $1 per preparation).

Supplementary Table. 6 | Comparison of TRACKer with other field-deployable methods.

Techniques	Bulky instrumentation required	Sensitivity	Sample to result	Cost per sample (USD)	Supplementary reference
CFPS	Yes	5fM	2.5h	5.48	15
STOPCovid.v2	No	100 copies	113-143min	4	16
Pan/Pf	Yes	16 parasites/μL	60min	4.02	17
EXTRA-CRISPR	Yes	fM	1.5h	0.6	18
ROSALIND	Yes	nM-μM	1-4h	0.67	19
TRACKer-LFA	No	4 pM	70min	2.07	This work

Supplementary References

1. Green, Alexander A., Silver, Pamela A., Collins, James J. & Yin, P. Toehold Switches: De-Novo-Designed Regulators of Gene Expression. *Cell* **159**, 925-939 (2014).
2. Wang, T. & Simmel, F.C. Riboswitch-inspired toehold riboregulators for gene regulation in Escherichia coli. *Nucleic Acids Research* **50**, 4784-4798 (2022).
3. Kim, J. et al. De novo-designed translation-repressing riboregulators for multi-input cellular logic. *Nat Chem Biol* **15**, 1173-1182 (2019).
4. Kim, J., Seo, M., Lim, Y. & Kim, J. START: A Versatile Platform for Bacterial Ligand Sensing with Programmable Performances. *Advanced Science* (2024).
5. Hong, F. et al. Precise and Programmable Detection of Mutations Using Ultraspecific Riboregulators. *Cell* **180**, 1018-1032.e16 (2020).
6. Zhang, D.Y. & Winfree, E. Control of DNA strand displacement kinetics using toehold exchange. *J Am Chem Soc* **131**, 17303-14 (2009).
7. De Castro, E. et al. ViralZone 2024 provides higher-resolution images and advanced virus-specific resources. *Nucleic Acids Res* **52**, D817-d821 (2024).
8. Pardee, K. et al. Rapid, Low-Cost Detection of Zika Virus Using Programmable Biomolecular Components. *Cell* **165**, 1255-1266 (2016).
9. Cao, M., Sun, Q., Zhang, X., Ma, Y. & Wang, J. Detection and differentiation of respiratory syncytial virus subgroups A and B with colorimetric toehold switch sensors in a paper-based cell-free system. *Biosensors and Bioelectronics* **182**(2021).
10. Köksaldı, İ.Ç. et al. SARS-CoV-2 Detection with De Novo-Designed Synthetic Riboregulators. *Analytical Chemistry* **93**, 9719-9727 (2021).
11. Takahashi, M.K. et al. A low-cost paper-based synthetic biology platform for analyzing gut microbiota and host biomarkers. *Nature Communications* **9**(2018).
12. Hunt, J.P. et al. Towards detection of SARS-CoV-2 RNA in human saliva: A paper-based cell-free toehold switch biosensor with a visual bioluminescent output. *New Biotechnology* **66**, 53-60 (2022).
13. Voyvodic, P.L. et al. Plug-and-play metabolic transducers expand the chemical detection space of cell-free biosensors. *Nature Communications* **10**(2019).
14. Phillips, E.A. et al. Detection of viral RNAs at ambient temperature via reporter proteins produced through the target-splinted ligation of DNA probes. *Nature Biomedical Engineering* **7**, 1571-1582 (2023).
15. Karlikow, M. et al. Field validation of the performance of paper-based tests for the detection of the Zika and chikungunya viruses in serum samples. *Nature Biomedical Engineering* **6**, 246-256 (2022).
16. Joung, J. et al. Detection of SARS-CoV-2 with SHERLOCK One-Pot Testing. *New England Journal of Medicine* **383**, 1492-1494 (2020).
17. Rakotomalala Robinson, D. et al. Sensitive near point-of-care detection of asymptomatic and submicroscopic Plasmodium falciparum infections in African endemic countries. *Nature Communications* **16**, 8925 (2025).
18. Yan, H. et al. A one-pot isothermal Cas12-based assay for the sensitive detection of microRNAs. *Nature Biomedical Engineering* **7**, 1583-1601 (2023).
19. Jung, J.K. et al. Cell-free biosensors for rapid detection of water contaminants. *Nature Biotechnology* **38**, 1451-1459 (2020).
