# Supplementary material for: De novo-designed ribozyme-controlled riboregulator for cell-free diagnostics
Source: Nat Commun. 2026 Apr 8;17:6189. doi: 10.1038/s41467-026-71684-6 (PMC13369928; doi:10.1038/s41467-026-71684-6)
Supplement: Supplementary file 3 — Description of Additional Supplementary Files [file 41467_2026_71684_MOESM3_ESM.pdf]

Description of Additional Supplementary Files

File Name: Supplementary Data 1

Description: Nucleic acid sequences.

File Name: Supplementary Data 2

Description: Gene information of respiratory virus target.
