# Supplementary material for: De novo-designed ribozyme-controlled riboregulator for cell-free diagnostics
Source: Nat Commun. 2026 Apr 8;17:6189. doi: 10.1038/s41467-026-71684-6 (PMC13369928; doi:10.1038/s41467-026-71684-6)
Supplement: Supplementary file 6 — Reporting Summary [file 41467_2026_71684_MOESM6_ESM.pdf]

nature portfolio

Corresponding author(s): Ming Chen, Kai Chang,

Last updated by author(s): Mar 15, 2026

Reporting Summary

Nature Portfolio wishes to improve the reproducibility of the work that we publish. This form provides structure for consistency and transparency in reporting. For further information on Nature Portfolio policies, see our Editorial Policies and the Editorial Policy Checklist.

Statistics

For all statistical analyses, confirm that the following items are present in the figure legend, table legend, main text, or Methods section.

n/a	Confirmed	
☐	☒	The exact sample size (*n*) for each experimental group/condition, given as a discrete number and unit of measurement
☐	☒	A statement on whether measurements were taken from distinct samples or whether the same sample was measured repeatedly
☐	☒	The statistical test(s) used AND whether they are one- or two-sided<br>*Only common tests should be described solely by name; describe more complex techniques in the Methods section.*
☒	☐	A description of all covariates tested
☐	☒	A description of any assumptions or corrections, such as tests of normality and adjustment for multiple comparisons
☐	☒	A full description of the statistical parameters including central tendency (e.g. means) or other basic estimates (e.g. regression coefficient) AND variation (e.g. standard deviation) or associated estimates of uncertainty (e.g. confidence intervals)
☐	☒	For null hypothesis testing, the test statistic (e.g. *F*, *t*, *r*) with confidence intervals, effect sizes, degrees of freedom and *P* value noted<br>*Give P values as exact values whenever suitable.*
☒	☐	For Bayesian analysis, information on the choice of priors and Markov chain Monte Carlo settings
☒	☐	For hierarchical and complex designs, identification of the appropriate level for tests and full reporting of outcomes
☒	☐	Estimates of effect sizes (e.g. Cohen's *d*, Pearson's *r*), indicating how they were calculated

Our web collection on statistics for biologists contains articles on many of the points above.

Software and code

Policy information about availability of computer code

Data collection	luminescence values were collected via SkanIt Software RE for Microplate Readers RE, (ver. 6.1.0.51). Lateral-flow test strips were imaged on Color Grab (Loomatix Technology, version 3.9.2).
Data analysis	SnapGene (Insightful Science, version 6.1.1), GraphPad Prism (GraphPad Software, version 10.1.2), Origin (OriginLab Corporation, version 10.1)

For manuscripts utilizing custom algorithms or software that are central to the research but not yet described in published literature, software must be made available to editors and reviewers. We strongly encourage code deposition in a community repository (e.g. GitHub). See the Nature Portfolio guidelines for submitting code & software for further information.

Data

Policy information about availability of data

All manuscripts must include a data availability statement. This statement should provide the following information, where applicable:

- Accession codes, unique identifiers, or web links for publicly available datasets
- A description of any restrictions on data availability
- For clinical datasets or third party data, please ensure that the statement adheres to our policy

All data needed to evaluate the conclusions can be found in the paper and its supplementary information. The proposed algorithm and scripts to screen for IRS is

available on GitHub (https://github.com/tanglab-coder/TRACKer-IRS-code) and Zenodo (https://doi.org/10.5281/zenodo.19019005).Source data are provided with this paper.

Research involving human participants, their data, or biological material

Policy information about studies with human participants or human data. See also policy information about sex, gender (identity/presentation), and sexual orientation and race, ethnicity and racism.

Reporting on sex and gender	Sex was reported where available. No gender-based analyses were performed.
Reporting on race, ethnicity, or other socially relevant groupings	Race, ethnicity, or other socially relevant groupings were not collected or analyzed in this study.
Population characteristics	Subjects considered for enrollment in this study were evaluated solely on the basis of protocol eligibility criteria. The summary of population characteristics is given in Supplementary Table 3.
Recruitment	Samples were discarded clinical specimens after routine testing. No active participant recruitment was performed.
Ethics oversight	The study was approved by the Ethics Committee of the First Affiliated Hospital of Army Medical University (approval number: (A)KY2025066).Informed consent was waived because only anonymized residual specimens were used.

Note that full information on the approval of the study protocol must also be provided in the manuscript.

Field-specific reporting

Please select the one below that is the best fit for your research. If you are not sure, read the appropriate sections before making your selection.

☒ Life sciences ☐ Behavioural & social sciences ☐ Ecological, evolutionary & environmental sciences

For a reference copy of the document with all sections, see nature.com/documents/nr-reporting-summary-flat.pdf

Life sciences study design

All studies must disclose on these points even when the disclosure is negative.

Sample size	No formal statistical power analysis was used to determine sample size. Sample sizes were determined based on the availability of discarded clinical specimens.
Data exclusions	No data were excluded.
Replication	All experiments were successfully replicated independently.
Randomization	Samples were not randomized. Group allocation was based on RT-qPCR results (negative or positive).
Blinding	The results of the analyses were objective and quantifiable, and not subject to investigator bias.

Reporting for specific materials, systems and methods

We require information from authors about some types of materials, experimental systems and methods used in many studies. Here, indicate whether each material, system or method listed is relevant to your study. If you are not sure if a list item applies to your research, read the appropriate section before selecting a response.

Materials & experimental systems

n/a	Involved in the study	
☐	☒	Antibodies
☒	☐	Eukaryotic cell lines
☒	☐	Palaeontology and archaeology
☒	☐	Animals and other organisms
☒	☐	Clinical data
☒	☐	Dual use research of concern
☒	☐	Plants

Methods

n/a	Involved in the study	
☒	☐	ChIP-seq
☒	☐	Flow cytometry
☒	☐	MRI-based neuroimaging

Antibodies

Antibodies used

anti-Flag-His tag protein detection test strips (Biodragon, cat. BF06219)

Validation

Antibodies were validated by the manufacturers by ELISA. We also validated each antibody by lateral-flow immunoassay format in which antibodies were striped onto a test membrane or conjugated to 40-nm gold nanoparticles. An antibody was considered valid if a test line appeared when subject to a synthesized peptide; no test line appeared if no peptide or off-target peptides were present.

Plants

Seed stocks

Report on the source of all seed stocks or other plant material used. If applicable, state the seed stock centre and catalogue number. If plant specimens were collected from the field, describe the collection location, date and sampling procedures.

Novel plant genotypes

Describe the methods by which all novel plant genotypes were produced. This includes those generated by transgenic approaches, gene editing, chemical/radiation-based mutagenesis and hybridization. For transgenic lines, describe the transformation method, the number of independent lines analyzed and the generation upon which experiments were performed. For gene-edited lines, describe the editor used, the endogenous sequence targeted for editing, the targeting guide RNA sequence (if applicable) and how the editor was applied.

Authentication

Describe any authentication procedures for each seed stock used or novel genotype generated. Describe any experiments used to assess the effect of a mutation and, where applicable, how potential secondary effects (e.g. second site T-DNA insertions, mosiacism, off-target gene editing) were examined.
