# Supplementary material for: De novo-designed ribozyme-controlled riboregulator for cell-free diagnostics
Source: Nat Commun. 2026 Apr 8;17:6189. doi: 10.1038/s41467-026-71684-6 (PMC13369928; doi:10.1038/s41467-026-71684-6)
Supplement: Supplementary file 2 — Peer Review File [file 41467_2026_71684_MOESM2_ESM.pdf]

nature portfolio

Peer Review File

De novo-designed ribozyme-controlled riboregulator for cell-free diagnostics

Corresponding Author: Dr Kai Chang

This file contains all reviewer reports in order by version, followed by all author rebuttals in order by version.

Version 0:

Reviewer comments:

Reviewer #1

(Remarks to the Author)
Tang et al present U-TRACKer, a novel detection system for viral RNAs.
While the idea of developing a detection system for viral RNAs is good and commendable, the manuscript as it stands is inaccurate, unclear and inconclusive in various instances, which casts doubt on the robustness of the method and on its potential to be applicable to diagnostic settings.

First, the molecular mechanism of U-TRACKer is unclear. The authors claim on aspect of novelty is the fact that their system is based on an OFF ribozyme which gets activated by the target, eliminating background leakage (line 75). But how is this system different from other riboregulators that are also inhibited in their basal form and get activated by the target (i.e. the toehold switch cited in ref 2 and others)? Indeed, fig 2c/d show substantial leakage (10-20%), which is expectable but contradicts the statement in line 75.
Moreover, from Fig 1 and the text, I do not understand the principle of action of U-TRACKer. The target binds the IRS sequence (violet), but displaces the switch from the yellow sequence. What triggers the displacement if it is not competition? Displacement then triggers cleavage and release of the coding RNA, but the figure shows that the RBS interacts with a complementary RBS* sequence external to the riboswitch. What induces the RBS from being released by the RBS* if these sequences are external and independent from the riboswitch?
Overall the mechanism of activation of U-TRACKer is thus poorly characterized and explained.

This weakness has implications in understanding the optimization of the reaction conditions, which is very confusing. The description of the design of the RNA sequences is very superficial (lines 203-223) with no experimental evidence of which sequences were actually screened and with what effects until converging on the final U-TRACKer product. Most importantly, though, all the experimental validation of U-TRACKer is weak. Figure S3 shows that the system is active under essentially all conditions displayed without significant differences. No experimental condition where the system is inactive is displayed suggesting that the device is very promiscuous and non specific. Indeed, Fig 2d shows that the system does not significantly distinguish between RNA and DNA inhibitory strands, differently from what is claimed in lines 148-150. Moreover, Fig 3c-g surprisingly show full activation even at time 0, contradicting the claim that 40 min is the best time for visualizing the results. Finally Fig 4a-f show that over a target RNA range of 5 orders of magnitude the U-TRACKer signal increases by less than 2 fold, which is clearly not a dose-dependent signal, as incorrectly claimed in lines 253-254.

Finally on page 13 the authors claim that there is no cross talk between cognate and non-cognate RNA targets but figure 4m/n show a different scenario. Fig 4m shows that there is up to 20-25% crosstalk. This is a bit less evident from Fig 4m, probably because of the color scale used by the authors. I am not sure this level of crosstalk would be compatible with diagnostic settings. Equally worrying is the level of false negative results (~10% in Fig 5bcd), which is anyway comparable and not superior (as claimed in line 367) to the only other diagnostic method compared here, that is qRT-PCR.

There are several other issues with the manuscript:

a) the authors claim that U-TRACKer is universal, but for each new target one would need to redesign the IRS sequence and optimize the system, as the authors have done for the 6 viruses that they studied. So the claims of "universality" are unclearly substantiated and contextualized.

b) the description of ribozymes in lines 59-62 is very poor. Ribozyme catalyze their reactions thanks to highly intricate tertiary structures, not just base-paired stems and structural simplicity. Moreover it is unclear what the authors mean with ribozyme's "modular versatility".

c) what secondary structure models do the free energy calculations presented in line131-137 and fig 2b refer to?

d) where exactly where placed FAM and BHQ1 in Fig 2a/lines 138-141?

e) MD simulations are said to be run in PyMol (line 159), but although I am not an expert in MD, I do not consider Pymol as the reference software for solid MD simulations. It would also be necessary to know which PDB files were used as starting models for such simulations and how the corresponding experimental structures were obtained.

f) in line 233 40 mmol should probably read 40 mM?

g) Materials (page 19): all materials used are of commercial origin. In this context, what is the actual chance that the technology developed by the authors (even if it was robust) would have any potential for scalability and use in point-of-care diagnostic centers?

Reviewer #2

(Remarks to the Author)
U-TRACKer is introduced as a universal diagnostic platform capable of sensitively detecting diverse viral RNAs without preamplification, combining a ribozyme-based riboregulator with a cell-free protein synthesis (CFPS) system. The authors claim it is applicable in field settings. However, some limitation requires clarification or validation throughout the manuscript:

1. (Page 7-12) Lack of Justification for Strand Displacement
In the proposed system, there is an absence of sequence overlap between the inhibition strand (IRS) and the target recognition segment. This approach deviates from the conventional toehold-mediated strand displacement models.
The only evidence presented in this paper is the result of free energy calculation on page 7, but it seems that a little more detailed explanation is needed to convince the reader. in Fig. 2b, the free energy calculation results of the IRS length between 10 nt and 18 nt were almost similar, while the difference in signal strength is significantly different in Fig. 2c. This raises questions about the validity of the calculation results. Even if IRS is designed with 18 nt of sequence, the simulation on NUPACK is expected to form the complex stably, and it seems thermodynamically reasonable.
U-TRACKer system achieving the 10 attomolar level of sensitivities. However, it has been well documented that the implementation of partial sequence overlap between the IRS and the target recognition region constitutes a more effective strategy for inducing strand displacement. NISDA, a non-enzymatic, isothermal strand displacement diagnosis assay, nevertheless contains toehold system, shows 133 aM of LoD, which is inferior than U-TRACKer. (This paper was even retracted recently due to reproducibility issues.) Translation-based signal amplification might be helpful; however, translation-based signal amplification alone is generally insufficient to achieve the non-amplified diagnosis, as shown at doi.org/10.1038/s41551-023-01028-y.
The claimed high sensitivity, which was also validated with clinical samples, is laudable but seems equivocal and under-supported. Further experimentation or theoretical support is necessary to elucidate the sensitivity. It would be also worthwhile to determine whether signals vanish when the IRS sequence is 19 or 20 nt, as shown in the simulation results.

2. (Fig. S3) Insufficient Information on Temperature Optimization
Temperature critically affects the performance of strand displacement reactions. Although Fig. S3 presents temperature-dependent luminescence data, it lacks comparison between target-positive and -negative samples. In order to provide all the necessary information to those who wish to use this technology, the experimental results for each temperature must be clearly indicated, showing the fluorescence values with and without the target, similar to the time-course graph shown on the side.

3. (Fig. S2) Ambiguous Interpretation of In Vitro Cleavage Experiments
Fig. S2 shows cleavage of the ~150 bp HiBiT switch. However, the bands formed as a result of cleavage appear to be similar to or lower than 50 bp. An explanation is requested for this observed discrepancy. If the problem is attributable to the formation of DNA structure, it is imperative that the results undergo re-examination using a denaturing gel.
In addition, it seems there should be a note to indicate how each band was generated.

4. (Fig. 4,5) Concerns About ROC Curve Validity
I would like to ask whether ROC curve analysis is an appropriate method for determining the threshold for diagnostic methods. The ROC analysis is only applicable when samples with known positive/negative results are available. The process entails the adjustment of the threshold to approximate the results with the greatest precision. When applying conventional standards, it is notable that a subset of samples depicted in Figure 5 present a challenge in terms of classification as positive.
Additionally, while Figure 4 shows a strong linear correlation between virus concentration and luminescence, such a correlation does not appear to be reproducible in clinical settings.

5. Limited Advantage of CFPS-Based Workflow
Despite avoiding nucleic acid amplification, the system still requires:
High-temperature (95 °C) RNA denaturation

Exogenous addition of LgBiT protein post-reaction
Multiple handling steps
These aspects diminish its practical advantages over RT-qPCR. To strengthen the case for CFPS, the manuscript should explore and present benefits like room-temperature storage or lyophilization feasibility.

6. (Page 15) Practical Challenges in Point-of-Care (POC) Use
Although the authors describe smartphone-based LFA signal quantification, several barriers remain:
RNA purification using commercial kits is still needed
The assay is sensitive to environmental contamination
Despite a claimed 4 pM LoD, there is no demonstration of robustness under field conditions
While smartphone-based RGB quantification is highlighted, the use of specialized holders and LED boxes undermines the Field-deployable claim.
These issues contradict the “equipment-free nucleic acid detection” (Page 15, line 309) diagnostic claim and suggest further simplification is necessary. If smartphones suffice, such auxiliary devices should be minimized or omitted.

7. (Page 13, Line 266-276 and Fig. 4m) Limitations in SNV Detection Design
Fig. 4m claims SNV discrimination, but wild-type and mutant targets were tested with identical U-TRACKer setups. This does not rule out signal differences due to RNA concentration rather than sequence mismatch. True SNV detection requires simultaneous testing with mutation-specific probes, particularly for relevant clinical applications like SARS-CoV-2 variant identification.

Minor comments
1. Feasibility of miRNA Detection
The authors state that IRS shorter than 18 nt promotes ribozyme activation. Does this imply that 18–22 nt-long miRNAs could be detected? Given their structural constraints and high specificity, miRNA detection remains a challenge, and further testing is warranted.

2. Potential for Catalytic Cascade Amplification
The current design relies on single-event ribozyme activation. Modifying the ribozyme or output sequence to trigger secondary activation cycles (catalytic amplification) could significantly boost sensitivity. This conceptual expansion is worth discussing.

Reviewer #3

(Remarks to the Author)
The authors present a highly sensitive approach to nucleic acid sensing using ribozyme-controlled riboregulator coupled with cell-free protein synthesis. The article is well written and of great interest to the cell-free sensing community. The paper is greatly strengthened by demonstrating use in clinical samples and as a field deployable technique. I have the following recommendations.

Major:
1) The use of “universal” in title seems a hyperbole. The authors are trying to convey that they do not have to re-engineer the riboregulator for each RNA target, which is something that toe-hold switches do require to a greater degree. However, it has not been exhaustively tested and there requires some engineering of the length of the RNA required for the desired sensitivity and limit of detection. I would therefore strongly recommend removing “universal” from the title and also temper the use of the word in the paper. I do not believe it will be universal as a target RNA sequence will need to bind to the strand that inhibits the ribozyme and pull it away. A desired RNA sequence could be tied up in a secondary structure such that it would not be able to bind to the strand that inhibits the ribozyme.
2) Line 129-130: I am not completely convinced from the results that the length of the IRS will not need to be reoptimized for many of the desired targets to obtain the limit of detection and noise to signal ratio desired.
3) Field-deployable U-TRACKer. I am very concerned that this RNA detection technique will have fundamental issues with RNAse especially in resource-limited environments. I do not see this working well in these samples simply due to RNAse degradation, especially at the aM concentrations they are seeking to detect down to. This is a massive problem and one that should be discussed and explained in greater detail. This further takes away from the “universal” claim of the paper. More discussion of how to deal with RNAse would be helpful.
4) An estimation of the cost of the field deployable application would strengthen the paper. It appears to using a phone to image capture but requires a specialized powered device and specialized antibodies coated with gold. This may be to costly for the resource-limited environments described.

Minor:
1) Figure 4a-5. It appears that detection at the 1 aM level is clearly distinguishable from the control. I am curious if the authors could detect at sub 1aM level. Am I seeing this incorrectly. ~10 aM is the limit of detection described in the paper.
2) There is substantially more discussion included in the results section than I would expect for a paper with a discussions section.
3) Figure S1 caption in supplementary lines 30-31: The yellow and blue designation is incorrect and needs to be switched. It is correct in the image just not in the caption.

Reviewer #4

(Remarks to the Author)
The manuscript by Yu Tang et al. presents the design and application of a ribozyme-based, cell-free diagnostic platform (U-TRACKer) capable of detecting multiple respiratory RNA viruses without preamplification. The authors propose a modular system consisting of a ribozyme allostery module, riboregulator activation module, and output module, achieving attomolar-level detection in both laboratory and field scenarios. The integration of IRS (Inhibition-Recognition Strand) to control ribozyme activation is an interesting mechanism to reduce background signal and increase orthogonality.Overall, the manuscript is well-organized and clearly written. The experimental validation is thorough and supported by both simulated and clinical data. However, several issues merit attention before publication.

Major Concerns:
1.Conceptual novelty and design depth:
While the use of a ribozyme-based regulation system is not unprecedented, the novelty of the current system lies primarily in the repackaging of known modules rather than in the fundamental innovation of mechanism. The IRS strategy, while effective, appears conceptually similar to prior strand displacement-triggered systems. The design simplicity, while contributing to ease of use, limits the mechanistic richness or tunability of the system. A more comprehensive comparison to related ribozyme and toehold switch-based approaches (e.g., in terms of complexity, dynamic range, orthogonality) is needed to better position this work in the current landscape.

2.Limited demonstration of universality:
The authors claim “universal detection,” yet all tested targets are respiratory viruses with relatively well-conserved RNA regions. It would be more convincing to test U-TRACKer with targets of greater sequence diversity or structural complexity (e.g., bacterial RNAs, miRNAs, or synthetic sequences with strong secondary structure).

3.IRS design lacks algorithmic generalizability:
The IRS design depends on sequence complementarity and structural predictions (e.g., NUPACK). However, no robust computational pipeline or rule set is offered to guide de novo IRS design. The system’s claimed scalability would benefit from a clearer design algorithm that allows other researchers to generate new IRSs for new targets with predictable performance.

4.Insufficient visual clarity of activation mechanism:
In the manuscript, the authors state that “when viral RNA hybridizes with the target recognition segment of the IRS, a strand displacement reaction is triggered, which in turn releases the ribozyme from inhibition. (Line 113-115)” However, this mechanism is not clearly conveyed in Figures 1 and 3b. Based on the current illustrations, it is difficult to visualize how hybridization of the viral RNA leads to strand displacement and subsequent ribozyme activation. A more detailed and mechanistically accurate diagram would greatly aid reader understanding.

5.Protein output module optimization could be further explained:
The use of HiBiT and Flag-His as reporter outputs is practical, but the trade-offs (e.g., luminescence vs. visual signal, sensitivity vs. simplicity) deserve further quantitative discussion. How does the modular output affect signal fidelity or cross-reactivity?

6.Questionable robustness of structure-based targeting strategy:
The authors state that “regions that did not form complex secondary structures were selected as targets” (Lines 241–242). While this rationale appears reasonable, the reported success rate—seemingly 100%—is somewhat surprising. In practice, even regions predicted to lack strong secondary structure (by NUPACK or similar tools) may still be sterically hindered or partially occluded by nearby structural elements, making them less accessible to complementary IRS sequences. It is unexpected that such a structure-based selection strategy would be uniformly effective across all tested targets. The apparent absence of any failed or suboptimal cases raises questions about the robustness of the targeting strategy, and more discussion or experimental data on potential limitations would enhance the credibility of this design approach.

Minor Comments:
1.Terminology clarity:
Terms such as "ribozyme allostery module" and "IRS" should be consistently defined early and used uniformly. In a few cases, "IRS" and "inhibition-recognition strand" appear with inconsistent capitalization and spacing.

2.Figure readability:
Figures 3 and 4 present strong data, but could benefit from clearer labeling of “+target” vs “–target” conditions and direct annotations of key parameters (e.g., LODs).

3.LFA device integration:
The field-deployable system is a valuable addition, but the application scope is not well discussed. Can the authors comment on storage stability, user handling tolerance, or batch-to-batch variation of test strips?

Recommendation:
The manuscript presents a solid, well-executed diagnostic platform with good performance metrics. However, due to the conceptual simplicity of the design, questions about mechanistic clarity, and limited demonstration of generalizability, I recommend major revision before publication. Expanding on the design framework, addressing limitations in the targeting

approach, and contextualizing the system more rigorously within current diagnostic innovations will significantly strengthen the manuscript.

Version 1:

Reviewer comments:

Reviewer #1

(Remarks to the Author)
The authors have revised the manuscript extensively, clarifying and addressing the reviewers concerns. The manuscript is thus now suitable for publication in Nature Communications.

(Remarks on code availability)

Reviewer #2

(Remarks to the Author)
A thorough review of the central claims in this paper reveals an apparent conflict with the well-established principles of toehold-mediated strand displacement used in nucleic acid diagnostics. This demonstrates the novelty of the research while simultaneously requiring a clear explanation of the mechanism. The proposed mechanism requires a more robust and convincing logic to align with the current understanding in the field. Basing the mechanism solely on simulation results provides insufficient evidence, since structural predictions for nucleic acids are often unreliable.
Additionally, the experimental design is inadequate to support the claim of POCT readiness. The clinical study employed a laboratory-based workflow, including RNA extraction, a process that is not compatible with a POCT setting. Furthermore, inadequacies of the clinical validation and the low LFA sensitivity suggest that the system is not yet field-deployable.
In the absence of a satisfactory resolution to the mechanistic discrepancy and POCT-appropriate validation, the manuscript does not currently meet the publication standards required for Nature Communications.

Comment #1

(The figures this reviewer simulated are presented in the attached PDF): NUPACK-predicted secondary structures and Gibbs free energies among target, HH15 ribozyme and IRS 18-1.
The figures present simulation results for systems with and without the HH15 ribozyme, with IRS 18-1. The left figure includes the IRS, target, and ribozyme sequence, while the right figure includes only the IRS and the target. The component sequences sourced from Supplementary Table 1 and a simulated temperature of 31°C. The omission of the simulation conditions from the manuscript hinders a thorough evaluation of the methodology's validity. Regardless, the free energy value from our simulation corresponds to that in Supplementary Figure 5 (-189.72 kcal/mol, IRS 18-1).
Our simulations using NUPACK indicate that the ternary complex, in which all three components are bound, is the most thermodynamically stable conformation. The reported dissociation of the ribozyme from the IRS and its subsequent activation is therefore not observed, irrespective of the IRS length. This outcome is readily explicable in terms of conventional thermodynamic expectations, since it minimizes single-strand region.
In the current revision, it appears that IRS 10–18 nt were analyzed as an IRS + target (two-component), whereas IRS 19–24 nt were analyzed as an IRS + target + ribozyme (three-component). Comparing absolute ΔG values across different chemical systems on the same axis can inflate or distort the apparent difficulty of strand displacement at longer IRS lengths. Moreover, when the ribozyme–substrate is also included for 10–18 nt (three-component), the ΔG differences may not be materially different from the 19–24 nt cases.
The paper posits that the IRS and ribozyme are generally applicable because their sequences are independent, which is a plausible claim. However, if these components are functionally independent as described, it seems that the binding between the IRS and the ribozyme should not be contingent upon target binding. While the explanation that the IRS induces the conformation change of the ribozyme to active form is feasible, the manuscript still does not adequately clarify how this mechanism relates to the specific length of the IRS.
Here are some requests:
- Explicitly state the parameters for simulation (ion concentration, temperature etc.) in the figure legend or methods section.
- Recompute and present results for all IRS lengths (10–24 nt) under the same system definition. At minimum, report the following separately for each length, under identical conditions:
(A) IRS + target; (B) switch + IRS + target.

Comment #2
Time-course luminescence analysis results are only shown for IRS 19-1 and IRS 20-1 in the supplementary figure 14. To substantiate the claim that ‘long IRS impedes strand displacement,’ readers might need a direct side-by-side comparison with IRS 18-1 (or 10–18 nt design) under the exactly same conditions and same axis scale.
Additionally, it seems inappropriate to explain that the decrease in signal is a evidence of the loss of strand displacement activity. The difference in signal intensity is clear at the 0 min time point in the Supplementary figure 13. Furthermore, Figure 3 also shows a decrease in signal.

Comment #3

The authors' response regarding the validation of the diagnostic results is insufficient. ROC analysis is a method to determine an optimal diagnostic threshold from a dataset where the outcomes are already known. This is to say, it has the problem of overfitting. It does not, inherently validate the assay's performance on an independent dataset.
A significant portion of the clinical results presented in this paper falls into an ambiguous "gray zone", making their classification as positive or negative problematic without relying on the RT-qPCR results used to generate the ROC curve. To properly substantiate the claimed sensitivity and specificity, the authors should conduct a blind test on a new cohort of samples using the threshold obtained from the ROC analysis; or establishing a more objective cutoff criterion (e.g., the mean of the negative control plus three standard deviations), similar to the methodology for determining the Limit of Detection (LoD).

Comment #4
The manuscript asserts POCT suitability, yet the primary comparator throughout is laboratory RT-qPCR, not a point-of-care diagnostic. The clinical “concordance” study relies on the same RNA purification and pretreatment used for RT-qPCR, which is not POCT-appropriate and therefore does not substantiate field deployability. Moreover, the POCT-compatible LFA exhibits a much weaker LoD in the picomolar (pM) range, which may be insufficient for detecting clinically relevant analyte concentrations.
Although it is mentioned in lines 352-354 that simplified RNA extraction can be applied, there is no experimental evidence which can support the statement. Please demonstrate performance with simplified or extraction-free sample preparation (crude lysis/swab-in-buffer) and report LoD and sensitivity/specificity in clinically relevant samples. If not, the claims regarding POCT readiness should be moderated.
Here are some recommendations:
- The manuscript should clarify current limitation and conclude that TRACKer shows potential for POCT, but that further engineering and validation is required.
- The authors should explicitly state that the current clinical comparison relied on laboratory-grade extraction and, thus does not by itself validate POCT performance.

(Remarks on code availability)

Reviewer #4

(Remarks to the Author)
The authors have carefully revised the manuscript and addressed most of my previous concerns. However, the revision has raised further doubts regarding the proposed mechanism of TRACKer. Specifically, in Figure 1a,b, the authors describe that upon target strand hybridization with the IRS strand, the IRS is displaced from the ribozyme loop region, thereby activating cleavage and releasing the RBS site. Yet, it is not clear that such displacement necessarily occurs. From the current design, the target strand could hybridize with the IRS without disrupting its interaction with the ribozyme loop, in which case the activation mechanism would not proceed as described. The authors should clarify, with stronger structural or mechanistic evidence, how target binding induces IRS release and ribozyme activation.

(Remarks on code availability)

Version 2:

Reviewer comments:

Reviewer #2

(Remarks to the Author)
The authors have comprehensively addressed the reviewers' comments. However, some uncertainty remains regarding the underlying mechanism, which was also raised by another reviewer. All other comments have been satisfactorily addressed.

Comment #1
The mechanistic inconsistencies have been partially resolved. The authors have incorporated necessary clarifications regarding simulation parameters, the consistency of ΔG comparisons across IRS lengths, and the interpretation of ternary complex formation. Supplementary Figures 5–7, together with the MD simulation analyses, now provide a coherent rationale regarding the relative binding affinities of the IRS–HH15 and IRS–target complexes.
However, an explanation regarding the instability of the ternary IRS-ribozyme-target complex remains elusive. Specifically, the driving force for target-induced strand displacement remains unclear, as there appears to be no obvious energetic advantage favoring competition between the target and the ribozyme for the IRS (as also asked by another reviewer). Notwithstanding this theoretical ambiguity, the experimental findings—such as those illustrated in Supplementary Fig. 8—empirically substantiate the authors' claims. Therefore, this mechanistic uncertainty becomes more important.

Comment #2
Experimental evidence supproting IRS length optimization has been appropriately reinforced. In alignment with the reviewer’s request, the dataset was expanded to include IRS lengths of 16–20 nt, evaluated under identical experimental conditions. The results confirm that the IRS with 18 nt complementarity represents the optimal configuration in terms of fold-

change performance, consistent with predictions.

Comment #3
The authors have validated the robustness of the ROC-derived threshold by applying it to independent sample cohorts. TRACKer demonstrated acceptable performance, and the concerns previously raised regarding the validation strategy have been adequately addressed.

Comment #4
Concerns regarding POCT applicability have been constructively addressed by the data presented in Supplementary Fig. 23. While the current pM-level of LoD remains modest for certain point-of-care contexts, the manuscript now appropriately acknowledges the need for further engineering and validation, having moderated the claims regarding field readiness.

(Remarks on code availability)

Reviewer #4

(Remarks to the Author)
The authors performed MD simulations and PAGE analyses to support the proposed TRACKer mechanism. However, based on the current data, this reviewer still has reservations about the mechanistic interpretation. Specifically, the simulations do not clearly explain why hybridization of the target strand to the IRS leads to displacement of the IRS from the ribozyme loop region. Consistent with the concerns raised by Reviewer 2, this reviewer has not previously encountered a scenario in which target–IRS pairing—which does not directly compete with the IRS–ribozyme loop interactions—would nevertheless be sufficient to dissociate the IRS from the loop. This observation challenges this reviewer's understanding of the classical principles governing nucleic-acid strand association and dissociation.

(Remarks on code availability)

Version 3:

Reviewer comments:

Reviewer #2

(Remarks to the Author)
The concern raised by this reviewer has been thoroughly addressed.

(Remarks on code availability)

Reviewer #4

(Remarks to the Author)
The authors have provided the additional clarification regarding the proposed toehold-mediated strand-displacement mechanism. While I appreciate the effort to address the mechanistic concerns, I remain unconvinced that the current explanation satisfactorily resolves the key issues.

1. Omission of the 12-nt exchange domain in earlier versions
According to the revised explanation, the 12-nt exchange domain—base-paired between the IRS and the riboswitch loop in the OFF state—is central to the proposed strand-displacement mechanism. The target initiates branch migration via the toehold, invades this 12-nt duplex, and ultimately displaces the incumbent strand.

If this 12-nt pairing is indeed mechanistically essential, it represents a critical structural determinant of the system. However, this feature was not clearly described in the original submission: 1) It was not emphasized in previous revisions, 2) It was only introduced after repeated concerns from two reviewers, 3) Moreover, this interaction is still not clearly illustrated in the main mechanistic figures (e.g., Figures 1 and 3), which gives the impression of a non-competitive or unconventional recognition mechanism. In its current presentation, the figures remain potentially misleading, as they do not transparently depict the competitive strand-displacement process now being invoked.

Given the centrality of this 12-nt duplex to the proposed mechanism, its late introduction and incomplete graphical representation raise substantial concerns about the clarity and robustness of the mechanistic model.

2. Stability of the residual 14-bp duplex
Even accepting the authors' proposed toehold-mediated strand-displacement model, a second issue remains unresolved. According to Supplementary Fig. 3, after branch migration across the 12-nt exchange domain, the IRS–target duplex remains associated with the riboswitch via ~14 nt of complementary pairing between IRS and the riboswitch loop domain. A 14-bp RNA duplex is typically thermodynamically stable under physiological conditions. It is therefore unclear why the IRS–

target complex would dissociate from the riboswitch entirely. The manuscript does not provide sufficient thermodynamic analysis, kinetic evidence, or structural validation to explain how this remaining duplex becomes destabilized.

Thus, even with the revised mechanistic explanation, the fundamental question remains:
What drives complete IRS dissociation despite the presence of a substantial remaining duplex?

For these reasons, I continue to have serious reservations regarding the mechanistic interpretation as currently presented.

(Remarks on code availability)

Version 4:

Reviewer comments:

Reviewer #5

(Remarks to the Author)
I believe that the authors have sufficiently addressed Reviewer #4's concerns about the toehold-mediated strand displacement. The gel in Supp. Fig. 10 is convincing experimentally. From thermodynamic theory/modeling, the binding of the Hibit switch region after Target binding involves 3 mismatches, then 5 paired bases (4 stacks), then 6 mismatches, then 13 paired bases (12 stacks). With high likelihood, the first group of 5 base pairs will not be stable given the fact the 3 middle bases are AAA|UUU, which are actually destabilizing base stacks for dsRNA. 12 base stacks is marginally stable at 37C, consistent with Reviewer #4's understanding, but that assumes thermodynamic equilibrium. With the depletion of products due to cleavage of the product, the riboswitch + Target binding becomes an irreversible reaction that seems reasonable to complete on the hour timescale.

(Remarks on code availability)

Open Access This Peer Review File is licensed under a Creative Commons Attribution 4.0 International License, which permits use, sharing, adaptation, distribution and reproduction in any medium or format, as long as you give appropriate credit to the original author(s) and the source, provide a link to the Creative Commons license, and indicate if changes were made.
In cases where reviewers are anonymous, credit should be given to 'Anonymous Referee' and the source.
The images or other third party material in this Peer Review File are included in the article’s Creative Commons license, unless indicated otherwise in a credit line to the material. If material is not included in the article’s Creative Commons license and your intended use is not permitted by statutory regulation or exceeds the permitted use, you will need to obtain permission directly from the copyright holder.
To view a copy of this license, visit https://creativecommons.org/licenses/by/4.0/

Response to reviewers' comments

To Reviewer 1:

Tang et al present U-TRACKer, a novel detection system for viral RNAs. While the idea of developing a detection system for viral RNAs is good and commendable, the manuscript as it stands is inaccurate, unclear and inconclusive in various instances, which casts doubt on the robustness of the method and on its potential to be applicable to diagnostic settings.

Reply: We sincerely thank the reviewer for the positive and constructive comments. We have improved the paper according to your suggestion and believe that the revised manuscript will be more persuasive.

First, the molecular mechanism of U-TRACKer is unclear. The authors claim on aspect of novelty is the fact that their system is based on an OFF ribozyme which gets activated by the target, eliminating background leakage (line 75). But how is this system different from other riboregulators that are also inhibited in their basal form and get activated by the target (i.e. the toehold switch cited in ref 2 and others)? Indeed, fig 2c/d show substantial leakage (10-20%), which is expectable but contradicts the statement in line 75.

Reply: Thanks for your suggestion. **The molecular mechanism of TRACKer** has been described in the revised manuscript (line 132-154, page 7-8). In addition, **the difference between TRACKer and conventional riboregulators** was illustrated in Supplementary Fig. 1. The conventional riboregulators depend on target-induced strand

displacement to disrupt stem-loop structures and expose the RBS. For different targets, conventional riboregulators require de novo design and laborious experimental validation. TRACKer incorporates IRS sequence as an intermediary: The target RNA displaces the IRS, which in turn activates ribozyme. This activated ribozyme cleaves the substrate and creates nicks, enabling the release of RBS and downstream reporter gene sequence. Importantly, TRACKer can accommodate different targets by simply replacing the IRS sequence, obviating the need for in silico modeling. The relevant description has been revised in manuscript (line 125-129, page 6-7).

Regarding **the leakage issue**, we apologize for any confusion caused by our description of leakage levels. We have revised the manuscript to state: "This system effectively reduces background leakage (line 77, page 4)."

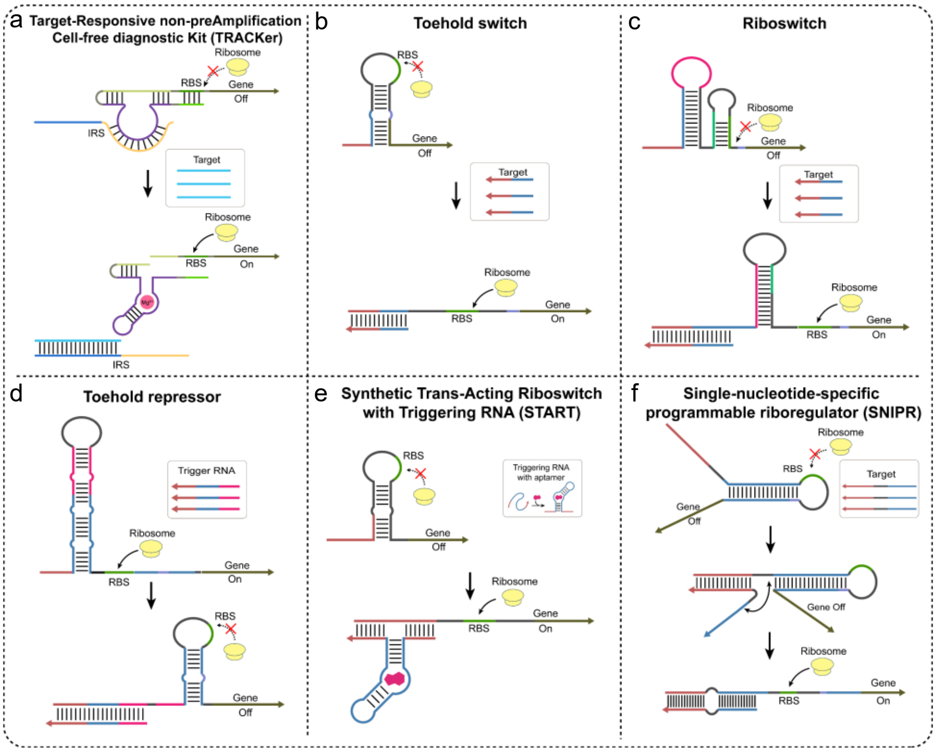

Supplementary Fig. 1 | Comparison of TRACKer with conventional riboregulators. a, TRACKer: the target displaces the IRS, which in turn activates ribozyme. This activated ribozyme

cleaves the substrate and creates nicks, enabling the release of the RBS and the downstream reporter gene sequence. **b**, Toehold switch: the target binds to the toehold region, which opens the stem-loop and exposes the RBS to initiate gene expression. **c**, Riboswitch: the target interacts with the toehold region, leading to the opening of the secondary structure of the RBS and thus triggering gene expression. **d**, Toehold repressor: the target binds to the toehold region, resulting in the formation of a stem-loop at the RBS and the inhibition of gene expression. **e**, Synthetic trans-acting riboswitch with triggering RNA (START): the target binds to the aptamer, causing the stem-loop to open and the RBS to be exposed for the initiation of gene expression. **f**, Single-nucleotide-specific programmable riboregulator (SNIPR): the target RNA binds to the forward toehold region, promoting the branch migration reaction and disrupting the base pairing in the reverse toehold region. This process opens the stem-loop and exposes the RBS to activate gene expression.

Moreover, from Fig 1 and the text, I do not understand the principle of action of U-TRACKer. The target binds the IRS sequence (violet), but displaces the switch from the yellow sequence. What triggers the displacement if it is not competition? Displacement then triggers cleavage and release of the coding RNA, but the figure shows that the RBS interacts with a complementary RBS* sequence external to the riboswitch. What induces the RBS from being released by the RBS* if these sequences are external and independent from the riboswitch? Overall, the mechanism of activation of U-TRACKer is thus poorly characterized and explained.

<u>Reply</u>: Thank you for the constructive comments. **The action principle of TRACKer** has been re-stated in the revised manuscript (line 115-122, page 6). To further illustrate **the competitive binding of TRACKer**, the Supplementary Fig.9 was added in the manuscript. The complementarity between target RNA and recognition strand (blue) of IRS was stronger than that between ribozyme and inhibition strand (yellow). This

thermodynamic preference drived strand displacement. The explanation has also been added in the Results section (line 117-118, page 6).

To demonstrate **the mechanism of RBS release**, we have added Supplementary Fig. 3. The release of RBS was induced by the target displacing IRS and activating ribozyme; this activated ribozyme cleaved the substrate to create nicks, in turn enabling the release of both the RBS and the downstream reporter gene sequence. The mechanism of RBS release was also illustrated in the gel electrophoresis (Supplementary Fig. 6).

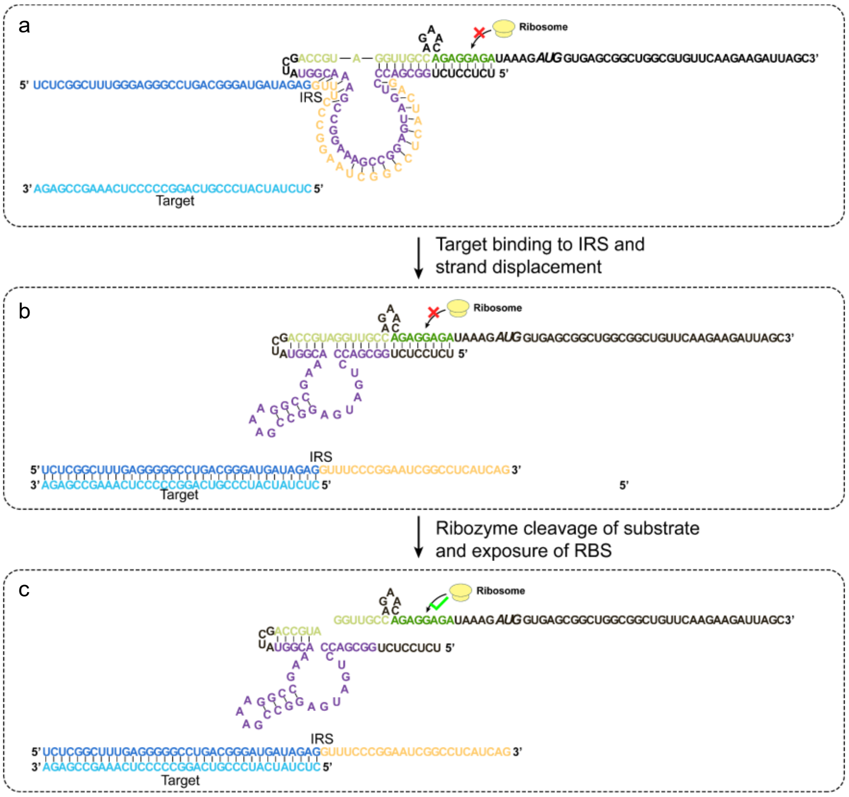

Supplementary Fig.9 | Schematic diagrams of TRACKer activation.

a-c, Diagrams with sequences illustrating the process of target FluA binding to the IRS 18-1, ribozyme cleavage of substrate, and exposure of RBS, respectively.

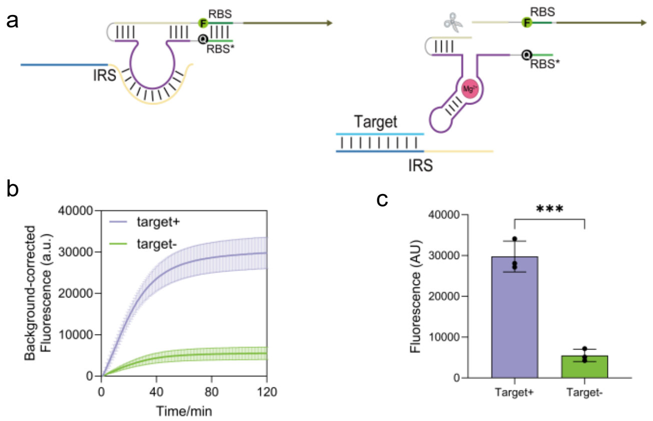

Supplementary Fig. 3 | FRET-based assessment of TRACKer conformational changes upon target binding.

a, Schematic illustration of the TRACKer design used to study molecular conformational changes following target addition, with fluorescein amidite (FAM) and black hole quencher 1 (BHQ1) labeled at the ribosome binding site (RBS) and RBS*, respectively. **b**, Time-dependent fluorescence curves in the presence or absence of the target. **c**, Endpoint fluorescence intensities with or without the target. Shaded regions represent the mean ± standard deviation (SD) from n = 3 technical replicates (***P = 0.0005; unpaired, two-tailed Student's t-test).

This weakness has implications in understanding the optimization of the reaction conditions, which is very confusing. The description of the design of the RNA sequences is very superficial (lines 203-223) with no experimental evidence of which sequences were actually screened and with what effects until converging on the final U-TRACKer product. Most importantly, though, all the experimental validation of U-TRACKer is weak. Figure S3 shows that the system is active under essentially all conditions displayed without significant differences. No experimental condition where the system is inactive is displayed suggesting that the device is very promiscuous and non-specific. Indeed, Fig 2d shows that the system does not significantly distinguish between RNA and DNA inhibitory strands, differently from what is claimed in lines 148-150. Moreover, Fig 3c-g surprisingly show full activation even at time 0,

contradicting the claim that 40 min is the best time for visualizing the results. Finally Fig 4a-f show that over a target RNA range of 5 orders of magnitude the U-TRACKer signal increases by less than 2 fold, which is clearly not a dose-dependent signal, as incorrectly claimed in lines 253-254.

Reply: We appreciate the reviewer's suggestions and address each concern as follows:

The **sequence design and optimization** of TRACKer has been restated in the manuscript (line 132-154, page 7-8). The sequences of IRS variants with different complementarity were shown in Supplementary Table 1. Through free energy calculation, fluorescence experiments, and electrophoresis verification, the IRS18-1/2/3 has been selected as the optimal choice. The relevant results were presented in Fig. 2b, Fig. 2c, and Supplementary Fig. 6.

Concerning **experimental condition optimization**, Supplementary Fig. 10 showed the optimization of conditions with and without the FluA target. The relevant descriptions have been revised in the manuscript (line 197-200, page 10).

Concerning **RNA vs. DNA IRS inhibition efficiency**, we apologize for the confusion caused by the imprecise description. We have revised the relevant content in the manuscript (line 149-150, page 7).

Regarding the **time course data**, in Fig. 3c-g, signal measurements start at time 0 following 40 min of cell-free expression, aiming to demonstrate the stability of TRACKer's expression. The relevant details have been revised in the figure legend (line 657-659, page 32). Additionally, 40 min was identified as the optimal duration for cell-free expression through experimental condition optimization, with the corresponding

explanation updated in the manuscript (line 197-200, page 10).

As for **the dose response**, Figs. 4a-f confirmed that the signal of TRACKer increased with increasing concentration of target RNA. What we intend to illustrate was quantifiable statistical relationships, rather than merely focusing on large increases alone. To avoid confusion, we have revised it to "concentration-dependent" (line 228, page 11).

Finally on page 13 the authors claim that there is no cross talk between cognate and non-cognate RNA targets but figure 4m/n show a different scenario. Fig 4m shows that there is up to 20-25% crosstalk. This is a bit less evident from Fig 4m, probably because of the color scale used by the authors. I am not sure this level of crosstalk would be compatible with diagnostic settings. Equally worrying is the level of false negative results (~10% in Fig 5bcd), which is anyway comparable and not superior (as claimed in line 367) to the only other diagnostic method compared here, that is qRT-PCR.

Reply: Thank you for the reviewer's suggestions. To clarify the issue of **signal leakage**, we have removed the signal values of blank control and re-analyzed the crosstalk. The corrected Figure 4g showed the relative signals of non-cognate targets reduced to 4%-13%. Moreover, no false positives were observed in the clinical validation (Fig. 5b-d), confirming this signal level meet diagnostic criteria. The relevant revisions have been updated in the manuscript (line 232-233, page 11).

Regarding the ~10% **false negatives** in Fig. 5b-d: the undetectable samples had RT-qPCR Ct values around 35, falling within the technique's "gray zone" (near detection

limit; Supplementary Fig.16-18) with extremely low target RNA. We have added relevant contents in the result section (line 260-262, page 13).

The "**superior performance**" claim for TRACKer in line 367 refers to comparisons with other cell-free platforms (Supplementary Table 6). Our data showed its sensitivity and specificity match RT-qPCR (Fig. 5e-f), with advantages of faster detection (<70 min) and no preamplification. These points have been clarified in the revised manuscript to avoid confusion (line 332-335, page 16).

a) the authors claim that U-TRACKer is universal, but for each new target one would need to redesign the IRS sequence and optimize the system, as the authors have done for the 6 viruses that they studied. So the claims of "universality" are unclearly substantiated and contextualized.

Reply: We are thankful for the reviewer's constrictive comments. In accordance with the suggestions of reviewer 3, we have redefined this technology as "Target-Responsive non-preAmplification Cell-free diagnostic Kit". Consequently, "U-TRACKer" has been renamed to "TRACKer". Additionally, the term "universal" indicates that TRACKer can accommodate different targets by simply replacing the IRS sequence, obviating the need for in silico modeling and laborious experimental optimization. Furthermore, we have removed the term "universal" from the title, and used the term "universal" sparingly throughout the manuscript to avoid confusion.

b) the description of ribozymes in lines 59-62 is very poor. Ribozyme catalyze their

reactions thanks to highly intricate tertiary structures, not just base-paired stems and structural simplicity. Moreover it is unclear what the authors mean with ribozyme's "modular versatility".

Reply: We sincerely appreciate the reviewer's insights. **The description of ribozymes** has been revised in the manuscript (line 60-62, page 3). In addition, the molecular simulations of TRACKer were shown in Supplementary Fig. 7-8, providing evidence that ribozyme catalysis relies on conformational changes and highly complex tertiary structural alterations.

The "Modular versatility" refers to the structural independence of ribozymes' components. These components can be split, recombined or modified via sequence design to fit different functional needs. The relevant references have been updated in the revised manuscript (Reference 13,15,16).

c) what secondary structure models do the free energy calculations presented in line131-137 and fig 2b refer to?

Reply: Thank you for the reviewer's suggestion. The secondary structure models derived from the NUPACK simulations have been included in Supplementary Fig. 5.

d) where exactly where placed FAM and BHQ1 in Fig 2a/lines 138-141?

Reply: Thank you for your comments. The specific positions of the FAM and BHQ1 labels have been supplemented in Supplementary Table 1.

e) MD simulations are said to be run in PyMol (line 159), but although I am not an expert in MD, I do not consider Pymol as the reference software for solid MD simulations. It would also be necessary to know which PDB files were used as starting models for such simulations and how the corresponding experimental structures were obtained.

Reply: We sincerely apologize for the confusion caused by the inaccurate description. The secondary structures of each sequence were constructed using oxDNA and saved as PDB files. The molecular simulations of the five systems were conducted using Gromacs 2021. The relevant corrections have been made in revised manuscript (line 402-412, page 19-20).

f) in line 233 40 mmol should probably read 40 mM?

Reply: Thank you for your suggestions. The relevant descriptions have been revised in manuscript (line 198, page 10).

g) Materials (page 19): all materials used are of commercial origin. In this context, what is the actual chance that the technology developed by the authors (even if it was robust) would have any potential for scalability and use in point-of-care diagnostic center?

Reply: Thank you for your suggestions. TRACKer can be flexibly adapted to both laboratory and field settings by modifying the reporter protein in its output module. In point-of-care testing, TRACKer enables naked-eye detection of multiple targets on a single commercial strip, avoiding complex antigen screening and streamlining the

development of target-specific assays. Traditional POCT methods often suffer from bulky instrumentation, strict temperature requirements, cold-chain storage, long assay times, and limited quantification. TRACKer addresses these challenges by relying on commercially available materials, ensuring consistent quality and robust supply chains. Its TRACKer-LFA system delivers results within 70 minutes at room temperature without specialized equipment. For pM-level quantification, only a smartphone and LED light are needed. Moreover, lyophilized cell-free reagents and LFA strips remain stable for up to one year at room temperature, further reducing cold-chain dependence. Thus, this technology is highly suited for POCT and has strong potential for large-scale deployment. The relevant discussion has been added in manuscript (line 348-367, page 17).

To Reviewer 2:

U-TRACKer is introduced as a universal diagnostic platform capable of sensitively detecting diverse viral RNAs without preamplification, combining a ribozyme-based riboregulator with a cell-free protein synthesis (CFPS) system. The authors claim it is applicable in field settings. However, some limitation requires clarification or validation throughout the manuscript:

Reply: We sincerely thank the reviewer for the positive feedback and valuable suggestion regarding to the manuscript. In the revised manuscript, more data and discussions are provided to fill the logical gaps and support our conclusions.

1. (Page 7-12) Lack of Justification for Strand Displacement

In the proposed system, there is an absence of sequence overlap between the inhibition strand (IRS) and the target recognition segment. This approach deviates from the conventional toehold-mediated strand displacement models.

The only evidence presented in this paper is the result of free energy calculation on page 7, but it seems that a little more detailed explanation is needed to convince the reader. in Fig. 2b, the free energy calculation results of the IRS length between 10 nt and 18 nt were almost similar, while the difference in signal strength is significantly different in Fig. 2c. This raises questions about the validity of the calculation results. Even if IRS is designed with 18 nt of sequence, the simulation on NUPACK is expected to form the complex stably, and it seems thermodynamically reasonable.

U-TRACKer system achieving the 10 attomolar level of sensitivities. However, it has been well documented that the implementation of partial sequence overlap between the IRS and the target recognition region constitutes a more effective strategy for inducing strand displacement. NISDA, a non-enzymatic, isothermal strand displacement diagnosis assay, nevertheless contains toehold system, shows 133 aM of LoD, which is inferior than U-TRACKer. (This paper was even retracted recently due to reproducibility issues.) Translation-based signal amplification might be helpful; however, translation-based signal amplification alone is generally insufficient to achieve the non-amplified diagnosis, as shown at doi.org/10.1038/s41551-023-01028-y. The claimed high sensitivity, which was also validated with clinical samples, is laudable but seems equivocal and under-supported. Further experimentation or

theoretical support is necessary to elucidate the sensitivity. It would be also worthwhile to determine whether signals vanish when the IRS sequence is 19 or 20 nt, as shown in the simulation results.

Reply: We are grateful for the reviewer's suggestions and respond to your concerns in detail as follows:

The overlap between the target sequence and IRS was supplied in Supplementary Fig. 9 and Supplementary Table 1, which formed the foundation of the competitive binding strand displacement reaction in TRACKer.

The secondary structure and binding free energies of the target, IRS, and HH15 was illustrated in Supplementary Fig.5. Notably, IRS10-18 show minimal differences in binding free energy, confirming strand displacement is energetically feasible. However, inhibition efficiency (Fig. 2c) not only depends on binding stability but also IRS's ability to induce the ribozyme into an inactive conformation. So, the varying complementary base lengths may cause significant efficiency differences. The relevant descriptions have been revised in manuscript (line 132-140;143-145, page 7).

The mechanism of high sensitivity was elucidated that the riboregulator activation module achieved cascade expression of reporter proteins through cell-free protein synthesis (line 91-92, page 5). Furthermore, the HiBiT reporter gene, which formed active nLuc with the LgBiT subunit and generated a signal 10^6-fold stronger than traditional luciferases (consistent with reported aM-level sensitivity of nLuc; doi.org/10.1038/s41551-023-01028-y). The corresponding discussion has been incorporated into the revised manuscript (line 190-194, page 9; line 223-224, page 11).

The capability of TRACKer constructed with IRS19-1/20-1 to detect FluA was shown in Supplementary Fig.13. The signal decreases significantly, consistent with our simulations indicating that strand displacement reactions rarely occur when complementary length exceed 18 nucleotides. The relevant descriptions have been added in manuscript (line 210-214, page 10).

2. (Fig. S3) Insufficient Information on Temperature Optimization

Temperature critically affects the performance of strand displacement reactions. Although Fig. S3 presents temperature-dependent luminescence data, it lacks comparison between target-positive and -negative samples. In order to provide all the necessary information to those who wish to use this technology, the experimental results for each temperature must be clearly indicated, showing the fluorescence values with and without the target, similar to the time-course graph shown on the side.

Reply: We are grateful for your suggestions. The temperature optimization between target-positive and target-negative samples have been illustrated in Supplementary Fig.10a.

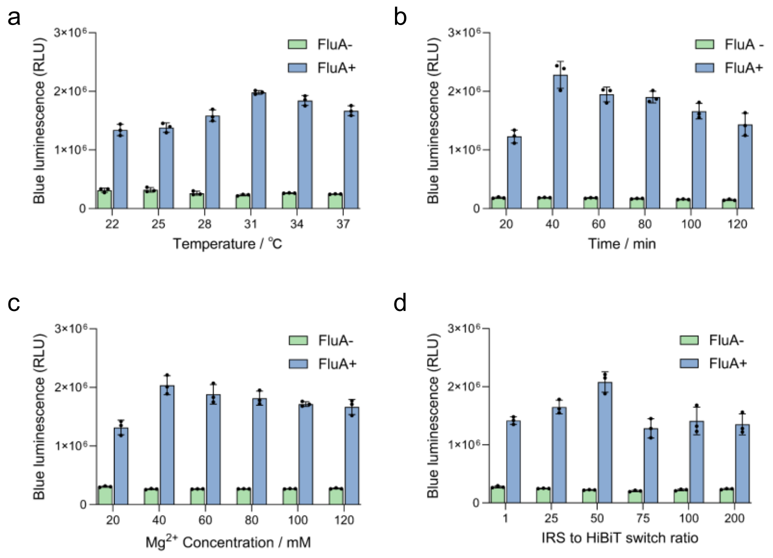

Supplementary Fig.10 | Optimization of reaction conditions for TRACKer.

a, Optimization of the incubation temperature for the interaction between IRS and the target. **b**, Optimization of the reaction time for TRACKer-based cell-free protein synthesis. **c**, Optimization of magnesium ion concentration in ribozyme-mediated reactions. **d**, Optimization of the concentration ratio between IRS and the HiBiT switch. All experimental measurements are mean ± standard deviation (SD) with n = 3.

3. (Fig. S2) Ambiguous Interpretation of In Vitro Cleavage Experiments

Fig. S2 shows cleavage of the ~150 bp HiBiT switch. However, the bands formed as a result of cleavage appear to be similar to or lower than 50 bp. An explanation is requested for this observed discrepancy. If the problem is attributable to the formation of DNA structure, it is imperative that the results undergo re-examination using a denaturing gel. In addition, it seems there should be a note to indicate how each band was generated.

Reply: Thank you for your suggestions. The denaturing gel electrophoresis of TRACKer have been reanalyzed in Supplementary Fig.6. And each band in the image has been labeled with corresponding explanations.

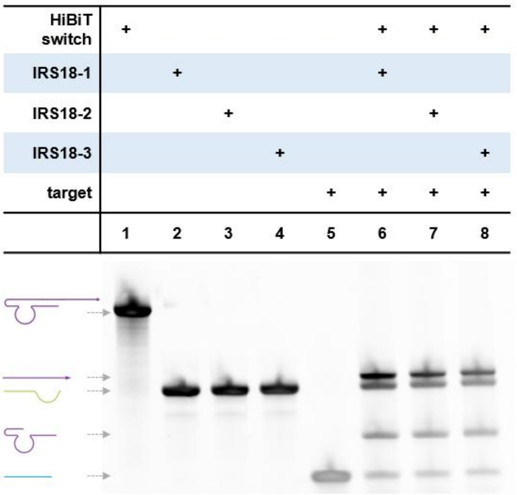

Supplementary Fig.6 | Urea-TBE polyacrylamide gel electrophoresis analysis for TRACKer.

(Lane 1) A long strand containing complementary sequences of ribosome-binding sites (RBS*), ribozymes and their substrates, ribosome binding site (RBS), and HiBiT templates, abbreviated as HiBiT switch

(Lane 2) IRS18-1 that can recognize FluA

(Lane 3) IRS18-2 that can recognize FluA

(Lane 4) IRS18-3 that can recognize FluA

(Lane 5) FluA

(Lane 6) When FluA was successfully recognized by IRS18-1, the ribozyme cleaved the substrate, splitting the HiBiT switch into two parts.

(Lane 7) When FluA was successfully recognized by IRS18-2, the ribozyme cleaved the substrate, splitting the HiBiT switch into two parts.

(Lane 8) When FluA was successfully recognized by IRS18-3, the ribozyme cleaved the substrate, splitting the HiBiT switch into two parts.

All the sequences used can be found in Supplementary Table 1.

4. (Fig. 4,5) Concerns About ROC Curve Validity

I would like to ask whether ROC curve analysis is an appropriate method for determining the threshold for diagnostic methods. The ROC analysis is only applicable when samples with known positive/negative results are available. The process entails

the adjustment of the threshold to approximate the results with the greatest precision. When applying conventional standards, it is notable that a subset of samples depicted in Figure 5 present a challenge in terms of classification as positive.

Additionally, while Figure 4 shows a strong linear correlation between virus concentration and luminescence, such a correlation does not appear to be reproducible in clinical settings.

Reply: We are grateful for the comments. The clinical validation of TRACKer used RT-qPCR as the reference method to classify 188 clinical pharyngeal swab samples as positive/negative, with all samples meeting the requirements for ROC curve analysis. In Figure 5, six samples misclassified by the TRACKer method had RT-qPCR Ct values around 35, falling within the technique's "gray zone" (near detection limit; see Supplementary Fig.16-18) with extremely low target RNA. The relevant contents have been added in manuscript (line 260-262, page 13). Overall, the consistency between TRACKer and RT-qPCR results ranges from 93.65% to 100%, confirming the validity of the ROC curve.

The linear correlation in clinical settings has been analyzed in Supplementary Fig.20, which demonstrated that the luminescence obtained with TRACKer were inversely correlated with the Ct values of RT-qPCR. The relevant descriptions have been added in manuscript (line 262-263, page 13).

5. Limited Advantage of CFPS-Based Workflow

Despite avoiding nucleic acid amplification, the system still requires:

λ High-temperature (95 °C) RNA denaturation

λ Exogenous addition of LgBiT protein post-reaction

λ Multiple handling steps

These aspects diminish its practical advantages over RT-qPCR. To strengthen the case for CFPS, the manuscript should explore and present benefits like room-temperature storage or lyophilization feasibility.

Reply: Thank you for your suggestions. The feasibility of room-temperature storage and lyophilization has been discussed in the revised manuscript (line 354-355, 358-360, page 17). The CFPS reagents were compatible with lyophilization and retain transcriptional and translational activity for up to one year under room-temperature storage (Cell. 2014 Nov 6;159(4):940-54).

6. (Page 15) Practical Challenges in Point-of-Care (POC) Use

Although the authors describe smartphone-based LFA signal quantification, several barriers remain:

λ RNA purification using commercial kits is still needed

λ The assay is sensitive to environmental contamination

λ Despite a claimed 4 pM LoD, there is no demonstration of robustness under field conditions

λ While smartphone-based RGB quantification is highlighted, the use of specialized holders and LED boxes undermines the Field-deployable claim.

These issues contradict the “equipment-free nucleic acid detection” (Page 15, line 309)

diagnostic claim and suggest further simplification is necessary. If smartphones suffice, such auxiliary devices should be minimized or omitted.

Reply: We are grateful for your suggestions. We have added the discussions on the practical challenges in POCT applications (line 348-367, page 17) and would like to clarify the relevant concerns as follows:

RNA purification: To verify the consistency between TRACKer and RT-qPCR in clinical samples, we used the same pretreatment method as commercial RT-qPCR for RNA extraction from pharyngeal swab samples. However, in actual POCT detection, TRACKer can adopt other simplified methods (e.g., lysis method) for RNA extraction. Relevant discussions have been added to the manuscript (line 349-354, page 17).

RNase contamination: In the practical application of POCT, we employed a one-tube reaction protocol: after the reaction was completed, the mixture was dropped onto the lateral flow assay (LFA) strip for naked-eye observation, which effectively mitigated the risk of RNase contamination. Relevant discussions have been added to the manuscript (line 355-357, page 17).

Robustness Under Field Conditions: The TRACKer functions normally at 22-37 °C, covering the common temperature range under field conditions (Supplementary Figure 10a). Furthermore, TRACKer was independent of bulky equipment: It enabled qualitative detection via naked-eye observation of results, while RGB-based quantitative detection through a palm-sized LED box. Additionally, the repeatability data presented in Figures 5d-f further confirmed its stability under field conditions. The relevant discussions have been added to the manuscript (line 358-361, page 17).

Portable devices: TRACKer-LFA enables qualitative detection via naked-eye observation. A palm-sized LED box (dimensions: 13 cm × 10 cm × 8 cm, Supplementary Fig 21) is optional for RGB quantification. Furthermore, this box costs $50 and supports thousands of repeated uses, thus per-use cost is less than $0.01. To avoid ambiguity, “equipment-free” has been revised to “instrument-free” (line 269, page 13).

7. (Page 13, Line 266-276 and Fig. 4m) Limitations in SNV Detection Design

Fig. 4m claims SNV discrimination, but wild-type and mutant targets were tested with identical U-TRACKer setups. This does not rule out signal differences due to RNA concentration rather than sequence mismatch. True SNV detection requires simultaneous testing with mutation-specific probes, particularly for relevant clinical applications like SARS-CoV-2 variant identification.

Reply: We sincerely apologize for any of our inaccurate description. The detection of mismatched FluA using TRACKer was solely intended to demonstrate the platform’s specificity from an alternative perspective. We have revised the relevant content to avoid confusion. (line232-235, page 11).

Minor comments

1. Feasibility of miRNA Detection

The authors state that IRS shorter than 18 nt promotes ribozyme activation. Does this imply that 18–22 nt-long miRNAs could be detected? Given their structural constraints

and high specificity, miRNA detection remains a challenge, and further testing is warranted.

<u>Reply</u>: We accept the reviewer's suggestion. The feasibility of TRACKer has been verified in Supplementary Fig 14. We detected microRNAs (miR-155/21), stem-loop-structure (C23/29), and DNA targets (KPC/NDM resistance genes), thus demonstrating the broad applicability of the TRACKer design. The relevant results have been added to the revised manuscript (line 215-221, page 11).

2. Potential for Catalytic Cascade Amplification

The current design relies on single-event ribozyme activation. Modifying the ribozyme or output sequence to trigger secondary activation cycles (catalytic amplification) could significantly boost sensitivity. This conceptual expansion is worth discussing.

<u>Reply</u>: Thanks for the reviewer's suggestion. The potential for catalytic cascade amplification can be achieved through two strategies: (1) engineering ribozyme substrates so their cleavage products act as secondary triggers to activate additional ribozyme switches, forming cyclic amplification loops; (2) designing reporter genes with small RNA motifs that displace IRS from ribozymes, creating self-amplifying cycles. These contents have been added to the Discussion section (lines 316-324, page 15-16).

To Reviewer 3:

The authors present a highly sensitive approach to nucleic acid sensing using ribozyme-

controlled riboregulator coupled with cell-free protein synthesis. The article is well written and of great interest to the cell-free sensing community. The paper is greatly strengthened by demonstrating use in clinical samples and as a field deployable technique. I have the following recommendations.

Reply: We sincerely appreciate your thorough review and the feedback provided. We responded in detail to the questions that were raised and provided the experimental support. The detailed corrections can be found below.

Major:

1) The use of "universal" in title seems a hyperbole. The authors are trying to convey that they do not have to re-engineer the riboregulator for each RNA target, which is something that toe-hold switches do require to a greater degree. However, it has not been exhaustively tested and there requires some engineering of the length of the RNA required for the desired sensitivity and limit of detection. I would therefore strongly recommend removing "universal" from the title and also temper the use of the word in the paper. I do not believe it will be universal as a target RNA sequence will need to bind to the strand that inhibits the ribozyme and pull it away. A desired RNA sequence could be tied up in a secondary structure such that it would not be able to bind to the strand that inhibits the ribozyme.

Reply: We are thankful for the reviewer's constrictive comments. In accordance with your suggestions, we have redefined this technology as "Target-Responsive non-preAmplification Cell-free diagnostic Kit". Consequently, "U-TRACKer" has been

renamed to “TRACKer”. Additionally, we have removed the term “universal” from the title, and used the term “universal” sparingly throughout the manuscript to avoid confusion.

2) Line 129-130: I am not completely convinced from the results that the length of the IRS will not need to be reoptimized for many of the desired targets to obtain the limit of detection and noise to signal ratio desired.

Reply: We sincerely apologize for any of our inaccurate description. The sequences of IRS variants with different complementarity were shown in Supplementary Table 1. Through free energy calculation, fluorescence experiments, and electrophoresis verification, we selected IRS18-1/2/3 as the optimal inhibition strand, and the relevant results were presented in Fig. 2b, Fig. 2c, and Supplementary Fig. 6. For different targets, the total length of the IRS varies due to differences in the length of the target recognition region. Additionally, we have designed code for screening IRS sequences for different targets (https://github.com/tanglab-coder/TRACKer-IRS-code). The relevant discussions have been added in the manuscript (line 342-347, page 16-17).

3) Field-deployable U-TRACKer. I am very concerned that this RNA detection technique will have fundamental issues with RNAse especially in resource-limited environments. I do not see this working well in these samples simply due to RNAse degradation, especially at the aM concentrations they are seeking to detect down to. This is a massive problem and one that should be discussed and explained in greater

detail. This further takes away from the “universal” claim of the paper. More discussion of how to deal with RNAse would be helpful.

Reply: We greatly appreciate to your suggestions. In the practical application of POCT, we employ a one-tube reaction protocol: after the reaction is completed, the mixture is dropped onto the lateral flow assay (LFA) strip for naked-eye observation, which effectively mitigates the risk of RNase contamination. In addition, we use RNase-free consumables (e.g., pipette tips, microcentrifuge tubes) to minimize contamination. The relevant contents have been added in the Discussion section (line 355-357, page 17).

4) An estimation of the cost of the field deployable application would strengthen the paper. It appears to using a phone to image capture but requires a specialized powered device and specialized antibodies coated with gold. This may be to costly for the resource-limited environments described.

Reply: Sincere thanks for your constructive suggestions. The cost estimation has been added in Supplementary Table 7. The TRACKer-LFA platform’s per-test material cost is approximately $1.69, including RNA templates, cell-free protein synthesis reagents, and lateral flow strips with gold-labeled antibodies. For smartphone readout, the LED illumination box costs $50 but supports tens of thousands of reuses, bringing per-use cost below $0.01. We have added the relevant contents to the Discussion section (line 363, page 17).

Minor:

1) Figure 4a-5. It appears that detection at the 1 aM level is clearly distinguishable from the control. I am curious if the authors could detect at sub 1aM level. Am I seeing this incorrectly. ~10 aM is the limit of detection described in the paper.

Reply: We are grateful for your insights. Following the editor's suggestions, we have replaced the bar charts and redrawn Fig. 4a-f to more clearly present the signal values generated by TRACKer when detecting targets at different concentrations. Although the signal trends were observed at concentrations below 1 aM in some cases, fluctuations in background measurements at such low levels made it difficult to statistically distinguish replicate samples. Therefore, we defined the LOD of six viruses as approximately 1-10 aM.

2) There is substantially more discussion included in the results section than I would expect for a paper with a discussions section.

Reply: Thanks for your suggestions. The excessive discussion previously included in the Results section has been moved to the Discussion section, ensuring proper section division as expected.

3) Figure S1 caption in supplementary lines 30-31: The yellow and blue designation is incorrect and needs to be switched. It is correct in the image just not in the caption.

Reply: Thank you for pointing out the mistake. The labels for yellow and blue in the caption have been revised in the supplementary Fig 4 (Page 7 of supplementary information).

To Reviewer 4:

The manuscript by Yu Tang et al. presents the design and application of a ribozyme-based, cell-free diagnostic platform (U-TRACKer) capable of detecting multiple respiratory RNA viruses without preamplification. The authors propose a modular system consisting of a ribozyme allostery module, riboregulator activation module, and output module, achieving attomolar-level detection in both laboratory and field scenarios. The integration of IRS (Inhibition-Recognition Strand) to control ribozyme activation is an interesting mechanism to reduce background signal and increase orthogonality.Overall, the manuscript is well-organized and clearly written. The experimental validation is thorough and supported by both simulated and clinical data. However, several issues merit attention before publication.

Reply: We appreciate the reviewer's positive evaluation on our TRACKer platform and the manuscript's quality. The noted issues have been addressed through revisions, and we hope the revised manuscript satisfies your expectations.

Major Concerns:

1.Conceptual novelty and design depth:

While the use of a ribozyme-based regulation system is not unprecedented, the novelty of the current system lies primarily in the repackaging of known modules rather than in the fundamental innovation of mechanism. The IRS strategy, while effective, appears conceptually similar to prior strand displacement-triggered systems. The design simplicity, while contributing to ease of use, limits the mechanistic richness or

tunability of the system. A more comprehensive comparison to related ribozyme and toehold switch-based approaches (e.g., in terms of complexity, dynamic range, orthogonality) is needed to better position this work in the current landscape.

Reply: We are pleased to accept your suggestions. The comprehensive comparison between conventional riboregulator systems and TRACKer were illustrated in Supplementary Fig.1: Conventional riboregulators depend on target-induced strand displacement to disrupt stem-loop structures and expose the RBS. For different targets, conventional riboregulators require de novo design and laborious experimental validation. TRACKer incorporates IRS sequence as an intermediary: The target RNA displaces the IRS, which in turn activates ribozyme. This activated ribozyme cleaves the substrate and creates nicks, enabling the release of the RBS and the downstream reporter gene sequence. Importantly, TRACKer can accommodate different targets by simply replacing the IRS sequence, obviating the need for in silico modeling. The relevant contents have been added in the revised manuscript (line 305-310, page 15).

Furthermore, the Supplementary Table.7 exhibits that TRACKer outperforms other cell-free diagnostic platforms in terms of detection time, sensitivity, and its ability to function without pre-amplification. The discussion has been added to the revised manuscript (line 332-335, page 16).

2.Limited demonstration of universality:

The authors claim “universal detection,” yet all tested targets are respiratory viruses with relatively well-conserved RNA regions. It would be more convincing to test U-

TRACKer with targets of greater sequence diversity or structural complexity (e.g., bacterial RNAs, miRNAs, or synthetic sequences with strong secondary structure).

Reply: Thank you for your constructive feedback. The feasibility of TRACKer has been verified in Supplementary Fig 14. We detected microRNAs (miR-155/21), stem-loop-structure (C23/29), and DNA targets (KPC/NDM resistance genes), thus demonstrating the broad applicability of the TRACKer design. The relevant results have been added to the revised manuscript (line 215-221, page 11).

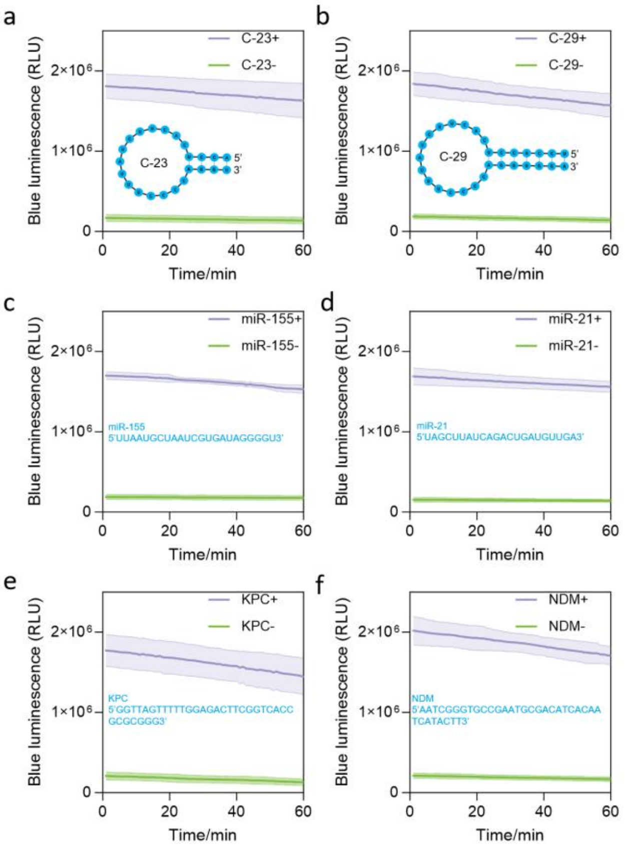

Supplementary Fig 14 | Time-course luminescence measurements of TRACKer for detection of six different target: C-23(a), C-29(b), miR-155(C), miR-21(d), KPC(e), and NDM(f). Blue luminescence was measured continuously for 60 minutes following the completion of the cell-free protein synthesis reaction and the addition of furimazine. Shaded regions represent mean ± standard deviation (SD), with n = 3 technical replicates.

3.IRS design lacks algorithmic generalizability:

The IRS design depends on sequence complementarity and structural predictions (e.g., NUPACK). However, no robust computational pipeline or rule set is offered to guide de novo IRS design. The system's claimed scalability would benefit from a clearer design algorithm that allows other researchers to generate new IRSs for new targets with predictable performance.

Reply: Thank you for your valuable insights. The computational pipeline for IRS design has been added in the website (https://github.com/tanglab-coder/TRACKer-IRS-code), which details how users can leverage NUPACK to design IRS sequences for different target RNA sequences.

4.Insufficient visual clarity of activation mechanism:

In the manuscript, the authors state that "when viral RNA hybridizes with the target recognition segment of the IRS, a strand displacement reaction is triggered, which in turn releases the ribozyme from inhibition. (Line 113-115)" However, this mechanism is not clearly conveyed in Figures 1 and 3b. Based on the current illustrations, it is difficult to visualize how hybridization of the viral RNA leads to strand displacement and subsequent ribozyme activation. A more detailed and mechanistically accurate diagram would greatly aid reader understanding.

Reply: We are grateful for your suggestions. The diagram illustrating the activation mechanism of TRACKer has been depicted in Supplementary Fig 2. In addition, the schematic that includes base sequences has been shown in Supplementary Fig 9.

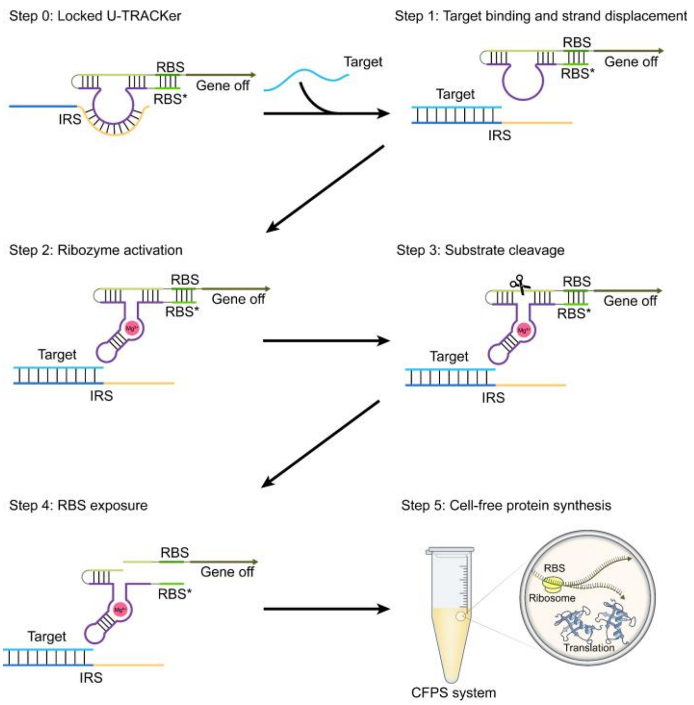

Supplementary Fig 2 | Molecular unlocking mechanism of TRACKer.

Hybridization of the IRS sequence with the target triggers a strand displacement reaction, resulting in ribozyme activation. The activated ribozyme cleaves the substrate strand, thereby unmasking the RBS and initiating reporter protein expression.

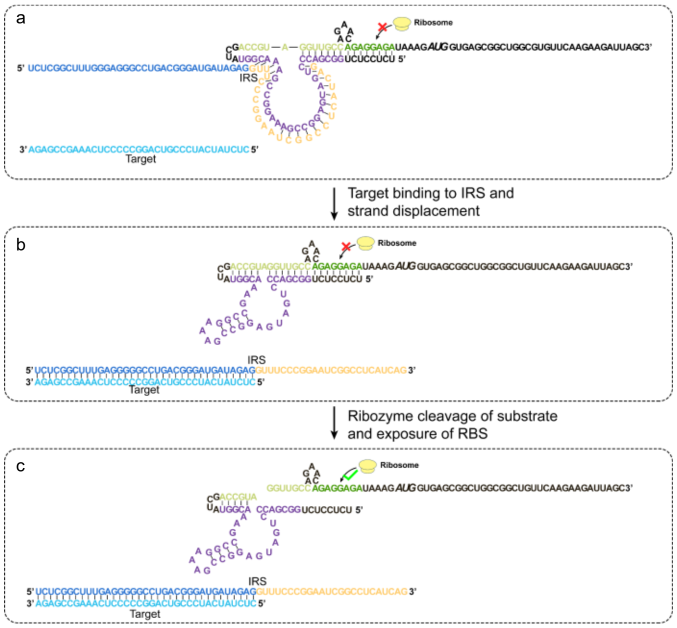

Supplementary Fig 9 | Schematic diagrams of TRACKer activation.

a-c, Diagrams with sequences illustrating the process of target FluA binding to the IRS 18-1, ribozyme cleavage of substrate, and exposure of RBS, respectively.

5.Protein output module optimization could be further explained:

The use of HiBiT and Flag-His as reporter outputs is practical, but the trade-offs (e.g., luminescence vs. visual signal, sensitivity vs. simplicity) deserve further quantitative discussion. How does the modular output affect signal fidelity or cross-reactivity?

Reply: We sincerely appreciate the reviewer's comments. The explanation of protein output module optimization has been updated in the Discussion section (line 325-332, page 16). HiBiT is suitable for laboratory settings requiring high sensitivity, though its application depends on relevant instrumentation. In contrast, Flag-His is specifically designed for on-site detection: while its sensitivity is slightly lower, it enables qualitative observation via naked-eye recognition and quantitative detection through smartphone-based identification. Furthermore, the output mode of TRACKer can also be adapted for a wider range of fields by modifying the reporter protein sequence.

The cross-reactivity of the system is not compromised by the two output modules, given that target recognition and translational regulation are mediated by the upstream ribozyme allostery module and riboregulator activation module.

6.Questionable robustness of structure-based targeting strategy:

The authors state that "regions that did not form complex secondary structures were selected as targets" (Lines 241-242). While this rationale appears reasonable, the reported success rate—seemingly 100%—is somewhat surprising. In practice, even regions predicted to lack strong secondary structure (by NUPACK or similar tools) may still be sterically hindered or partially occluded by nearby structural elements, making

them less accessible to complementary IRS sequences. It is unexpected that such a structure-based selection strategy would be uniformly effective across all tested targets. The apparent absence of any failed or suboptimal cases raises questions about the robustness of the targeting strategy, and more discussion or experimental data on potential limitations would enhance the credibility of this design approach.

Reply: Thank you for your suggestions. The potential limitations about the robustness of the structure-based targeting strategy have been discussed in detail (line 336-347, page 16-17). Although our structure-based targeting strategy is effective for all viral targets tested, the hybridization efficiency may be reduced by steric hindrance or partial occlusion in computationally accessible regions. The potential trouble makes experimental validation a necessary step to verify robust signal generation from in silico selected sites in TRACKer.

Minor Comments:

1.Terminology clarity:

Terms such as "ribozyme allostery module" and "IRS" should be consistently defined early and used uniformly. In a few cases, "IRS" and "inhibition-recognition strand" appear with inconsistent capitalization and spacing.

Reply: We are grateful for your suggestions. “Ribozyme allostery module” and “inhibition-recognition strand (IRS)” have been consistently defined at the beginning of the manuscript, and their usage has been standardized throughout. Capitalization of “IRS” has been unified, and the hyphenation of “inhibition-recognition strand” has been

corrected for consistency. A thorough recheck confirms all terms are now consistently defined and presented throughout the manuscript.

2.Figure readability:

Figures 3 and 4 present strong data, but could benefit from clearer labeling of “+target” vs “–target” conditions and direct annotations of key parameters (e.g., LODs).

Reply: Thank you for your valuable suggestions. The labels of the “+target” and “–target” groups in Figures 3 and 4 have been made more distinguishable through optimization. Additionally, LOD values have been directly annotated in the figures, with the core data information rendered more readable.

3.LFA device integration:

The field-deployable system is a valuable addition, but the application scope is not well discussed. Can the authors comment on storage stability, user handling tolerance, or batch-to-batch variation of test strips?

Reply: We are grateful for your suggestions. The discussion about the application scope have been added in the revised manuscript (line 348-364, page 17). Regarding **storage stability**, the LFA strip can be stored at room temperature for up to one year, and lyophilized CFPS reagents remain active after one year of room-temperature storage. In terms of **user handling tolerance**, the entire assay operates at ambient temperature, and a portable device integrating battery-powered LED illumination with smartphone-based image capture minimizes user subjectivity and enhances reproducibility in

resource-limited settings. With respect to **batch-to-batch variation of test strips**, commercial pre-coated LFA strips were evaluated across three batches, all of which showed low inter-batch variation, consistent control line intensity, and stable visual detectability at concentrations ≥4 pM.

Recommendation:

The manuscript presents a solid, well-executed diagnostic platform with good performance metrics. However, due to the conceptual simplicity of the design, questions about mechanistic clarity, and limited demonstration of generalizability, I recommend major revision before publication. Expanding on the design framework, addressing limitations in the targeting approach, and contextualizing the system more rigorously within current diagnostic innovations will significantly strengthen the manuscript.

Reply: We sincerely appreciate your recognition of our diagnostic platform. We have improved the paper according to your suggestion and believe that the revised manuscript will be more convincing.

Response to reviewers' comments

To Reviewer #1:

The authors have revised the manuscript extensively, clarifying and addressing the reviewers concerns. The manuscript is thus now suitable for publication in Nature Communications.

Reply: We thank the reviewers for their valuable feedback and for considering our manuscript for publication in *Nature Communications*.

To Reviewer #2:

A thorough review of the central claims in this paper reveals an apparent conflict with the well-established principles of toehold-mediated strand displacement used in nucleic acid diagnostics. This demonstrates the novelty of the research while simultaneously requiring a clear explanation of the mechanism. The proposed mechanism requires a more robust and convincing logic to align with the current understanding in the field. Basing the mechanism solely on simulation results provides insufficient evidence, since structural predictions for nucleic acids are often unreliable.

Additionally, the experimental design is inadequate to support the claim of POCT readiness. The clinical study employed a laboratory-based workflow, including RNA extraction, a process that is not compatible with a POCT setting. Furthermore, inadequacies of the clinical validation and the low LFA sensitivity suggest that the system is not yet field-deployable.

In the absence of a satisfactory resolution to the mechanistic discrepancy and POCT-appropriate validation, the manuscript does not currently meet the publication standards required for Nature Communications.

Reply: We are grateful to the reviewers for their valuable suggestions. The raised points have been carefully considered and addressed. Specifically, supplementary experiments

were added to clarify the mechanism, and POCT-oriented validation was performed. We are confident that these revisions have significantly strengthened the manuscript and addressed the reviewer's concerns.

Comment #1

(The figures this reviewer simulated are presented in the attached PDF): NUPACK-predicted secondary structures and Gibbs free energies among target, HH15 ribozyme and IRS 18-1.

The figures present simulation results for systems with and without the HH15 ribozyme, with IRS 18-1. The left figure includes the IRS, target, and ribozyme sequence, while the right figure includes only the IRS and the target. The component sequences sourced from Supplementary Table 1 and a simulated temperature of 31°C. The omission of the simulation conditions from the manuscript hinders a thorough evaluation of the methodology's validity. Regardless, the free energy value from our simulation corresponds to that in Supplementary Figure 5 (-189.72 kcal/mol, IRS 18-1).

Our simulations using NUPACK indicate that the ternary complex, in which all three components are bound, is the most thermodynamically stable conformation. The reported dissociation of the ribozyme from the IRS and its subsequent activation is therefore not observed, irrespective of the IRS length. This outcome is readily explicable in terms of conventional thermodynamic expectations, since it minimizes single-strand region.

In the current revision, it appears that IRS 10–18 nt were analyzed as an IRS + target (two-component), whereas IRS 19–24 nt were analyzed as an IRS + target + ribozyme (three-component). Comparing absolute ΔG values across different chemical systems on the same axis can inflate or distort the apparent difficulty of strand displacement at longer IRS lengths. Moreover, when the ribozyme–substrate is also included for 10–18 nt (three-component), the ΔG differences may not be materially different from the 19–

24 nt cases.

The paper posits that the IRS and ribozyme are generally applicable because their sequences are independent, which is a plausible claim. However, if these components are functionally independent as described, it seems that the binding between the IRS and the ribozyme should not be contingent upon target binding. While the explanation that the IRS induces the conformation change of the ribozyme to active form is feasible, the manuscript still does not adequately clarify how this mechanism relates to the specific length of the IRS.

Here are some requests:

- Explicitly state the parameters for simulation (ion concentration, temperature etc.) in the figure legend or methods section.
- Recompute and present results for all IRS lengths (10–24 nt) under the same system definition. At minimum, report the following separately for each length, under identical conditions:

(A) IRS + target; (B) switch + IRS + target.

Reply: Thank you for the insightful suggestions. All NUPACK simulations have been repeated for all IRS variants (10-24 complementary bases) under consistent system definitions. The simulation parameters are detailed in the corresponding figure legends (Supplementary Figs. 5-7) as follows: the nucleic acid type was chosen to be 'RNA'; the 'dangles' parameter was set to 'all'; the temperature was set to 31 °C; the salt condition was Na^{+}1.0M and Mg^{2+}0M (as defined by NUPACK's RNA folding model).

Under these conditions, the analysis of equilibrium concentrations in the ternary system (HH15 + IRS + Target) in Supplementary Fig. 5 reveals a critical transition governed by IRS-HH15 complementarity:

- With **10-18 complementary bases with HH15**, the dominant state features the Target bound to a dissociated IRS, confirming strand displacement feasibility.
- With **19-24 complementary bases with HH15**, the most stable state is the ternary complex with all three strands bound, preventing strand displacement.

This transition is explained by the binding free energies of IRS-HH15 and IRS-Target, as shown in Supplementary Figs. 6 and 7. While the IRS-Target binding energy remains constant, the IRS-HH15 interaction strengthens progressively with complementarity length. This indicates that when complementarity between IRS and HH15 exceeds 18 bases, the strand displacement reaction between target and IRS becomes significantly more challenging.

Complementing these thermodynamic predictions, molecular dynamics simulations in Supplementary Fig. 9 visualize target-induced conformational changes in the IRS-HH15 complex. These simulations demonstrate an increased molecular distance between IRS and HH15 upon target binding, confirming that target addition directly promotes the dissociation of IRS18-1 from HH15. Relevant descriptions of these findings have been added to the manuscript (lines 134-145, page 7).

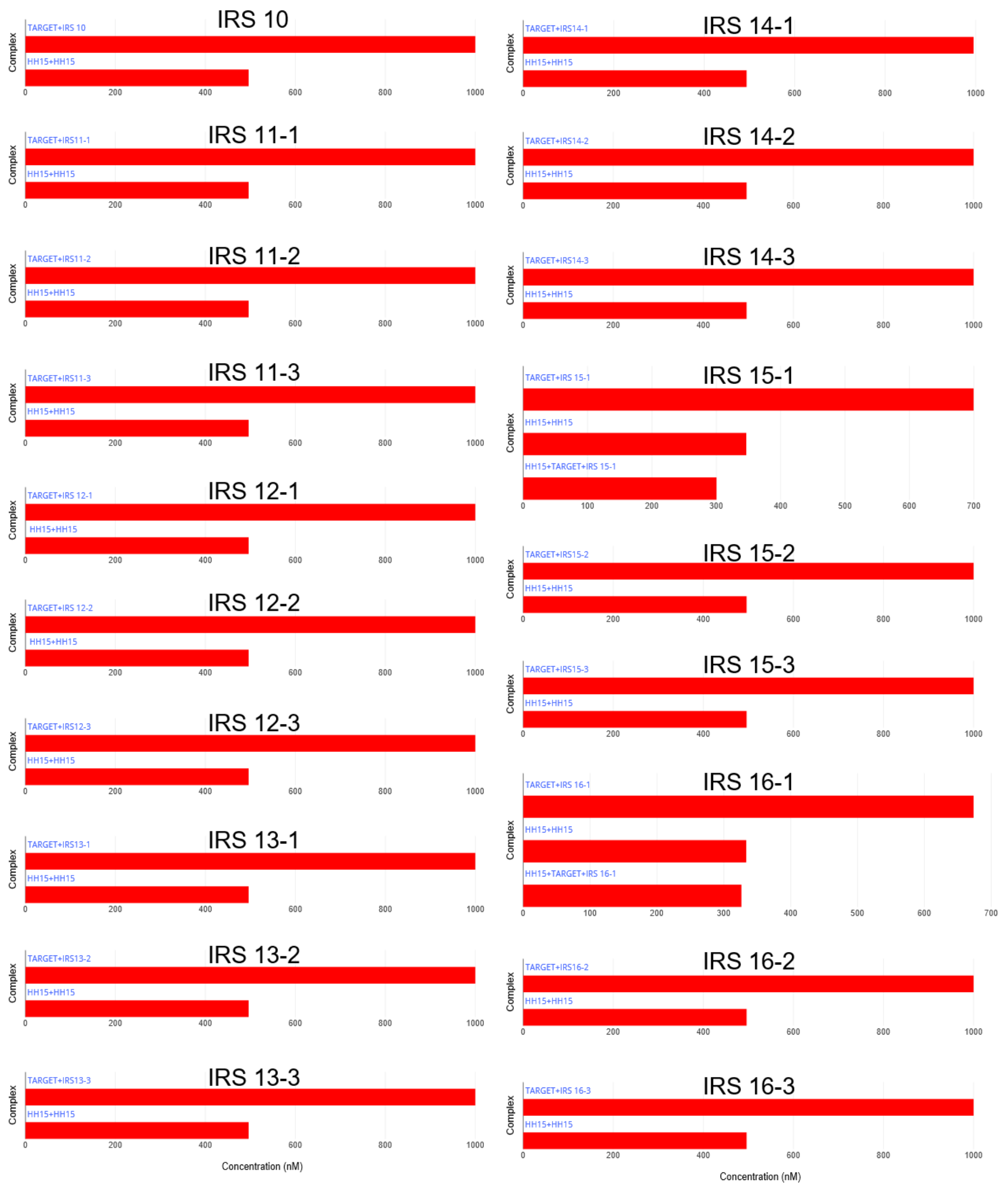
IRS 10
TARGET+IRS 10
HH15+HH15
IRS 11-1
TARGET+IRS11-1
HH15+HH15
IRS 11-2
TARGET+IRS11-2
HH15+HH15
IRS 11-3
TARGET+IRS11-3
HH15+HH15
IRS 12-1
TARGET+IRS 12-1
HH15+HH15
IRS 12-2
TARGET+IRS 12-2
HH15+HH15
IRS 12-3
TARGET+IRS12-3
HH15+HH15
IRS 13-1
TARGET+IRS13-1
HH15+HH15
IRS 13-2
TARGET+IRS13-2
HH15+HH15
IRS 13-3
TARGET+IRS13-3
HH15+HH15
IRS 14-1
TARGET+IRS14-1
HH15+HH15
IRS 14-2
TARGET+IRS14-2
HH15+HH15
IRS 14-3
TARGET+IRS14-3
HH15+HH15
IRS 15-1
TARGET+IRS 15-1
HH15+HH15
HH15+TARGET+IRS 15-1
IRS 15-2
TARGET+IRS15-2
HH15+HH15
IRS 15-3
TARGET+IRS15-3
HH15+HH15
IRS 16-1
TARGET+IRS 16-1
HH15+HH15
HH15+TARGET+IRS 16-1
IRS 16-2
TARGET+IRS16-2
HH15+HH15
IRS 16-3
TARGET+IRS 16-3
HH15+HH15
Complex
Concentration (nM)

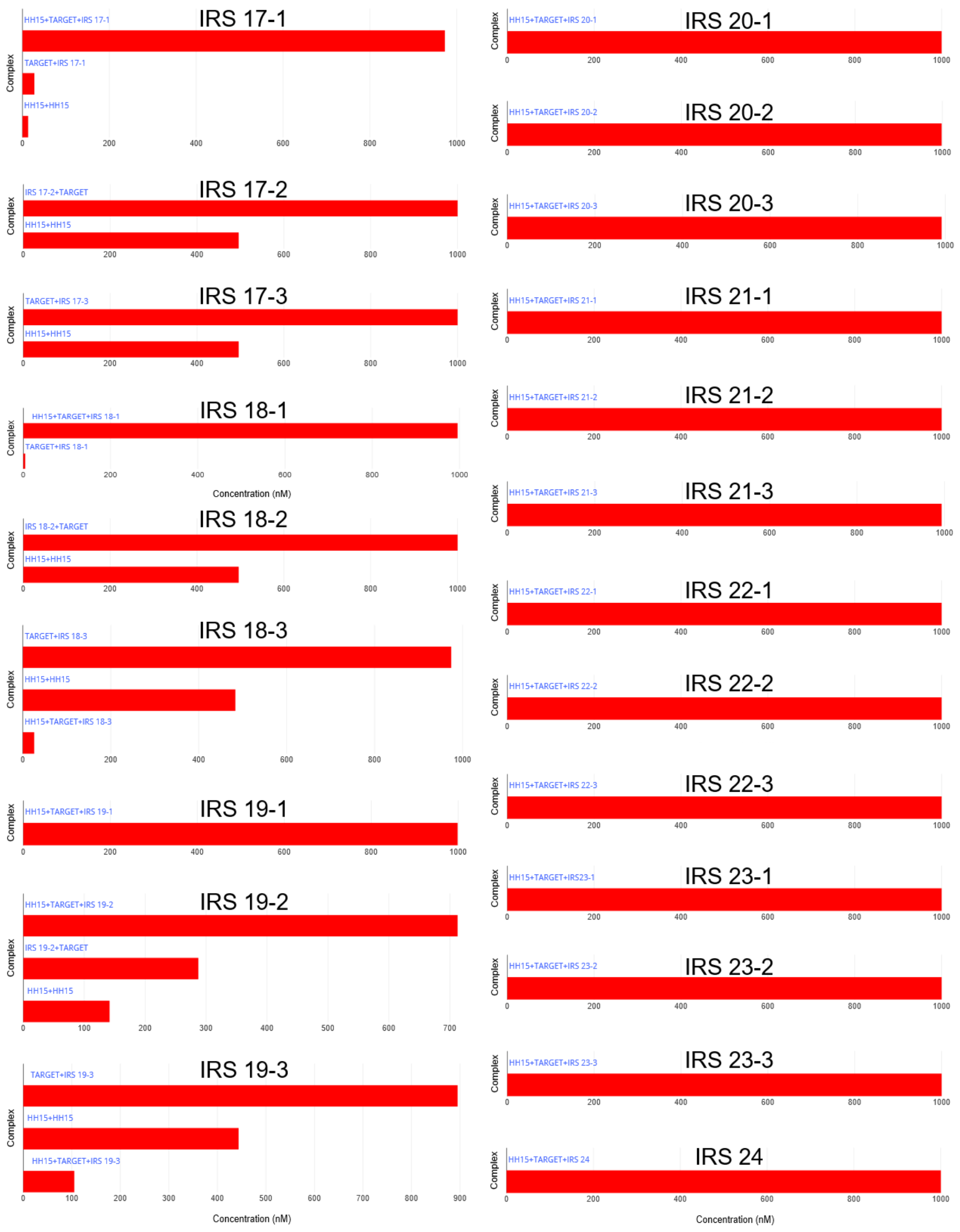

Supplementary Fig.5| NUPACK-predicted equilibrium complex concentrations among target, HH15 ribozyme and IRS of different complementary lengths.

The structure was predicted using NUPACK based on the conditions as follows: The nucleic acid type was chosen to be ‘RNA’; the salt condition was Na+1.0M and Mg2+0M (the only supported conditions for RNA); the ‘dangles’ parameter was set to ‘all’; the temperature was set to 31 °C (the temperature at which TRACKer assay was performed).

Comment #2

Time-course luminescence analysis results are only shown for IRS 19-1 and IRS 20-1 in the supplementary figure 14. To substantiate the claim that 'long IRS impedes strand displacement,' readers might need a direct side-by-side comparison with IRS 18-1 (or 10–18 nt design) under the exactly same conditions and same axis scale.

Additionally, it seems inappropriate to explain that the decrease in signal is a evidence of the loss of strand displacement activity. The difference in signal intensity is clear at the 0 min time point in the Supplementary figure 13. Furthermore, Figure 3 also shows a decrease in signal.

Reply: We are thankful for the reviewer's constructive comments. Based on the simulation results shown in Supplementary Fig. 5, the differences in TRACKer with IRS 16-20 was compared. The capability of TRACKer constructed with IRS16-1/17-1/18-1/19-1/20-1 to detect FluA is presented in Supplementary Fig. 15. The results indicate that longer IRS strands impede strand displacement. Relevant descriptions have also been added to the manuscript (lines 209-211, page 10).

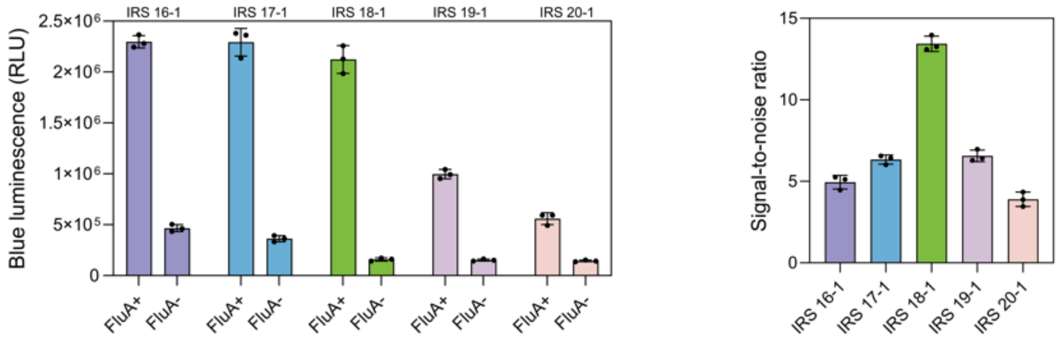

Supplementary Fig. 15 | a, Endpoint luminescence of TRACKer for FluA detection with IRS16-1, 17-1, 18-1, 19-1, and 20-1. b, Signal-to-noise ratio of TRACKer for FluA detection with IRS16-1, 17-1, 18-1, 19-1, and 20-1.

Blue luminescence was measured after cell-free protein synthesis reaction completion and furimazine addition. All experimental measurements are mean ± standard deviation (SD), n = 3.

Comment #3

The authors' response regarding the validation of the diagnostic results is insufficient. ROC analysis is a method to determine an optimal diagnostic threshold from a dataset where the outcomes are already known. This is to say, it has the problem of overfitting. It does not, inherently validate the assay's performance on an independent dataset.

A significant portion of the clinical results presented in this paper falls into an ambiguous "gray zone", making their classification as positive or negative problematic without relying on the RT-qPCR results used to generate the ROC curve. To properly substantiate the claimed sensitivity and specificity, the authors should conduct a blind test on a new cohort of samples using the threshold obtained from the ROC analysis; or establishing a more objective cutoff criterion (e.g., the mean of the negative control plus three standard deviations), similar to the methodology for determining the Limit of Detection (LoD).

Reply: Thanks for the reviewer's suggestion. The blind test on a new sample cohort using the ROC-derived threshold is presented in Supplementary Fig.21. Specifically, TRACKer was applied to 25 samples from suspected FluA-infected individuals, 35 from suspected HrV-infected individuals, and 37 from suspected RSV-infected individuals. Positivity and negativity were determined using the threshold established by the ROC curve of Fig 5f. When assessed against clinical screening tests via a confusion matrix, TRACKer achieved a clinical sensitivity and specificity of 100% and 94.8% for FluA, 90% and 92% for HrV, and 88.9% and 96.4% for RSV, confirming the assay's robust performance. Relevant descriptions have also been added to the manuscript (lines 255-261, page 13).

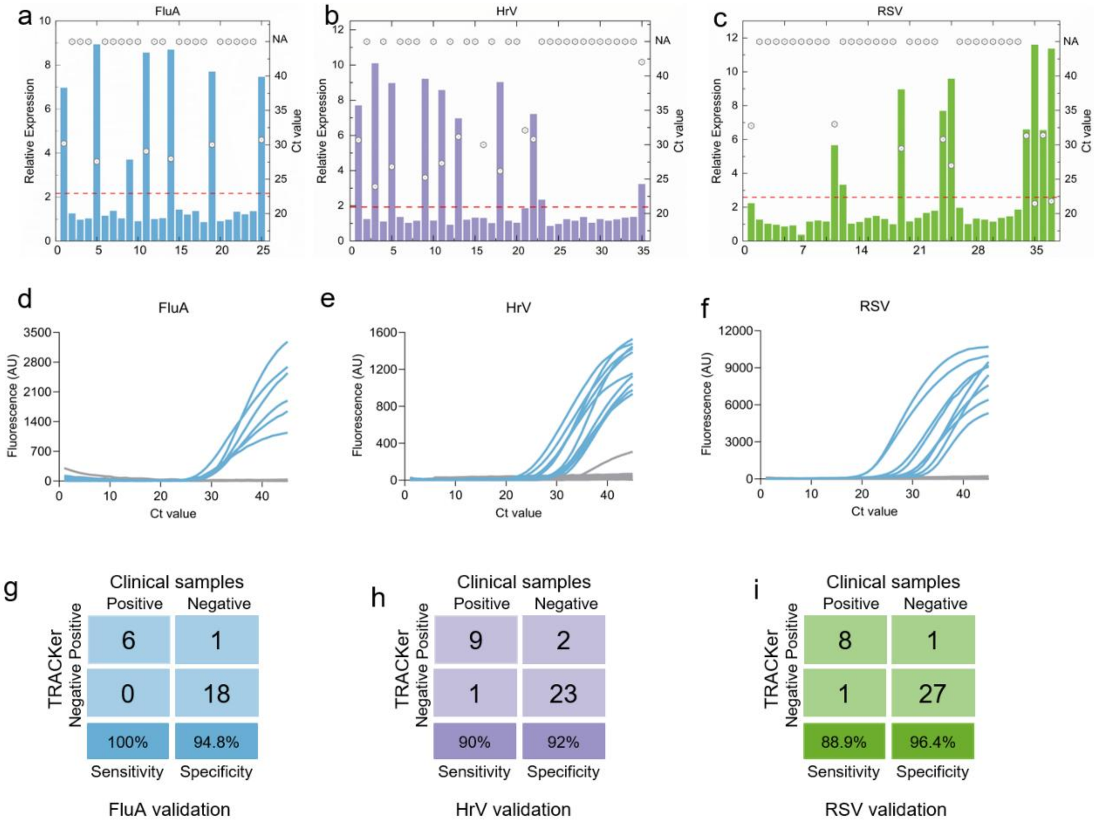

Supplementary Fig.21 | Clinical validation of TRACKer's diagnostic performance. **a-c**, Comparative analysis of FluA (**a**), HrV (**b**), and RSV (**c**) between RT-qPCR and TRACKer assays. Histograms depict relative expression levels, defined as the ratio of RLUgene to RLUnegative (RLUgene and RLUnegative denote RLU values for target viral genes and negative controls, respectively). Gray hexagons represent RT-qPCR cycle threshold (Ct) values. Red dashed lines indicate relative expression cut-offs determined by ROC curve analysis in Fig. 5f. **d-f**, Fluorescence curves of FluA (**d**), HrV (**e**), and RSV (**f**). **g-i**, Clinical sensitivity and specificity of TRACKer for detecting FluA (**g**), HrV (**h**), and RSV (**i**), assessed against clinical screening tests via a confusion matrix. NA in **a-c** indicates no Ct value.

Comment #4

The manuscript asserts POCT suitability, yet the primary comparator throughout is laboratory RT-qPCR, not a point-of-care diagnostic. The clinical "concordance" study relies on the same RNA purification and pretreatment used for RT-qPCR, which is not POCT-appropriate and therefore does not substantiate field deployability. Moreover, the POCT-compatible LFA exhibits a much weaker LoD in the picomolar (pM) range,

which may be insufficient for detecting clinically relevant analyte concentrations. Although it is mentioned in lines 352-354 that simplified RNA extraction can be applied, there is no experimental evidence which can support the statement. Please demonstrate performance with simplified or extraction-free sample preparation (crude lysis/swab-in-buffer) and report LoD and sensitivity/specificity in clinically relevant samples. If not, the claims regarding POCT readiness should be moderated.

Here are some recommendations:

- The manuscript should clarify current limitation and conclude that TRACKer shows potential for POCT, but that further engineering and validation is required.
- The authors should explicitly state that the current clinical comparison relied on laboratory-grade extraction and, thus does not by itself validate POCT performance.

Reply: We thank the reviewer for this suggestion. The performance of TRACKer-LFA with simplified sample preparation is shown in Supplementary Fig.23. The limit of detection (LOD) of TRACKer-LFA for FluA pseudoviruses following simplified extraction was 6.64pM. The clinical sensitivity and specificity of TRACKer-LFA, assessed against clinical screening tests using a confusion matrix, were 92.3% and 100%, respectively (Fig.23e). These results underscore the potential of TRACKer-LFA for point-of-care testing (POCT). In the manuscript, we also discuss current limitations and conclude that while TRACKer shows promise for POCT, further engineering and clinical validation are necessary. We will continue to optimize the system in future work to fully meet POCT requirements. Relevant descriptions have been added to the manuscript (lines 296–300, page 14; lines 346–349, page 17).

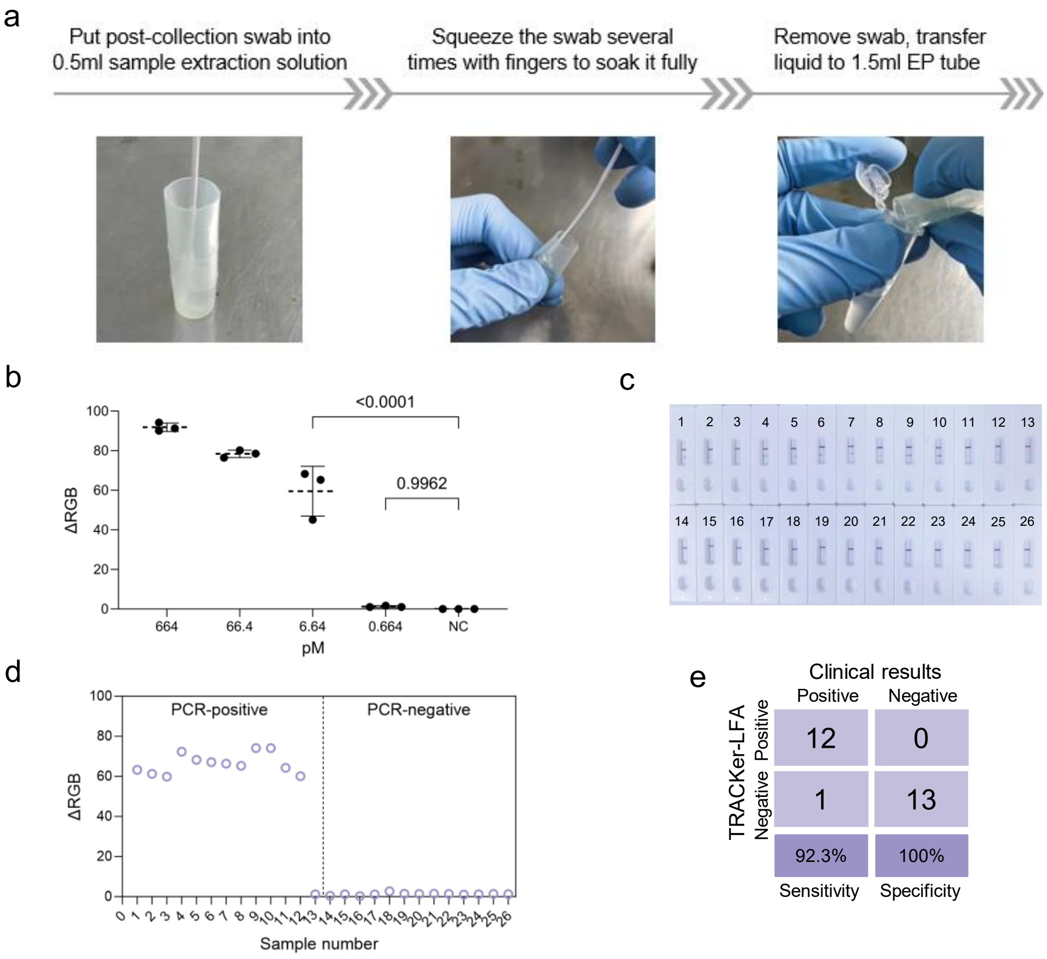

Supplementary Fig.23 | Diagnostic performance of TRACKer-LFA with simplified sample preparation. a, Schematic of nucleic acid extraction from swabs of suspected cases using sample extraction solution (main components: 10 mM Tris + 0.1% Tween-20). **b**, Limit of detection (LOD) of TRACKer-LFA for H1N1 pseudoviruses following simplified extraction. All experimental measurements are mean ± standard deviation (SD) with n = 3. **c**, Visual detection of 13 FluA-positive and 13 FluA-negative clinical samples via TRACKer-LFA with simplified extraction. **d**, ΔRGB values from testing 26 clinical FluA swab samples. **e**, Clinical sensitivity and specificity of TRACKer-LFA, assessed against clinical screening tests using a confusion matrix (n = 13 for negative samples and n = 13 for positive samples).

To Reviewer #4:

The authors have carefully revised the manuscript and addressed most of my previous concerns. However, the revision has raised further doubts regarding the proposed mechanism of TRACKer. Specifically, in Figure 1a, b, the authors describe that upon target strand hybridization with the IRS strand, the IRS is displaced from the ribozyme loop region, thereby activating cleavage and releasing the RBS site. Yet, it is not clear that such displacement necessarily occurs. From the current design, the target strand could hybridize with the IRS without disrupting its interaction with the ribozyme loop, in which case the activation mechanism would not proceed as described. The authors should clarify, with stronger structural or mechanistic evidence, how target binding induces IRS release and ribozyme activation.

Reply: We thank the reviewer for these suggestions. The NUPACK-simulated equilibrium concentrations of complexes formed by HH15, target, and IRS with varying numbers of bases complementary to HH15 are presented in Supplementary Fig. 5. These simulations reveal that when IRS shares 10-18 complementary bases with HH15, target binding effectively induces IRS release. In contrast, with 19-24 complementary bases, target, IRS, and HH15 form a stable ternary complex, preventing target-induced IRS dissociation.

The underlying thermodynamic mechanism is further elucidated by the secondary structures and binding free energies in Supplementary Figs. 6 and 7. As complementarity between IRS and HH15 increases, the free energy of the IRS–HH15 pair strengthens progressively, while that of the IRS–target pair remains largely constant. This explains why strand displacement becomes thermodynamically unfavorable once IRS–HH15 complementarity exceeds 18 bases. These results align well with the inhibition efficiency measured for different IRS variants (Fig. 2c) and support the selection of IRS18 as the optimal sequence.

Additionally, molecular dynamics simulations in Supplementary Fig. 9 visualize target-induced conformational changes in the IRS-HH15 complex. These simulations

demonstrate an increased molecular distance between IRS and HH15 upon target binding, confirming that target addition directly promotes the dissociation of IRS18-1 from HH15.

Finally, the overall activation mechanism is clarified in Supplementary Fig. 3: target-induced IRS release restores ribozyme cleavage activity, resulting in a turn-on fluorescent signal. This process is further validated by gel electrophoresis (Supplementary Fig. 8).

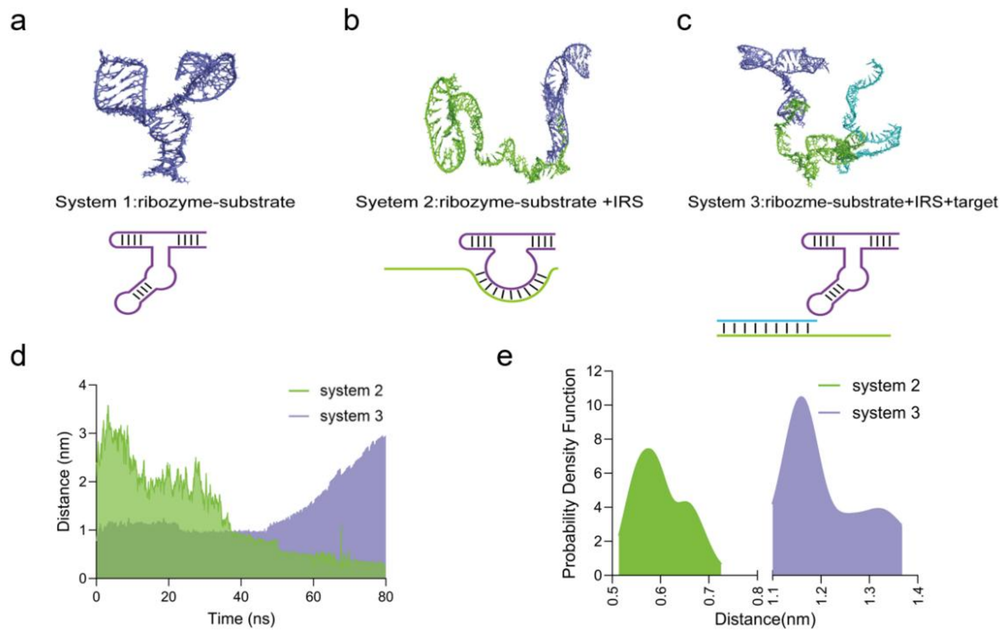

Supplementary Fig. 9| Mechanistic insights into IRS-mediated ribozyme inhibition and target recognition. a, Ribozyme-substrate complex (system 1) exhibiting the stable secondary structure necessary for cis-cleavage. **b,** The ribozyme-substrate complex with IRS (system 2): IRS obstructs the ribozyme-substrate interaction, inhibiting cleavage activity. **c,** Ribozyme-substrate complex with IRS and target RNA (system 3): Target RNA displaces IRS, enabling structural reformation and catalytic reactivation. **d,** Time-dependent base-pair distance between ribozyme-substrate and IRS in systems 2 and 3 during 80 nanosecond(ns) simulations. **e,** Probability density distributions of base-pair distances for Systems 2 and 3.

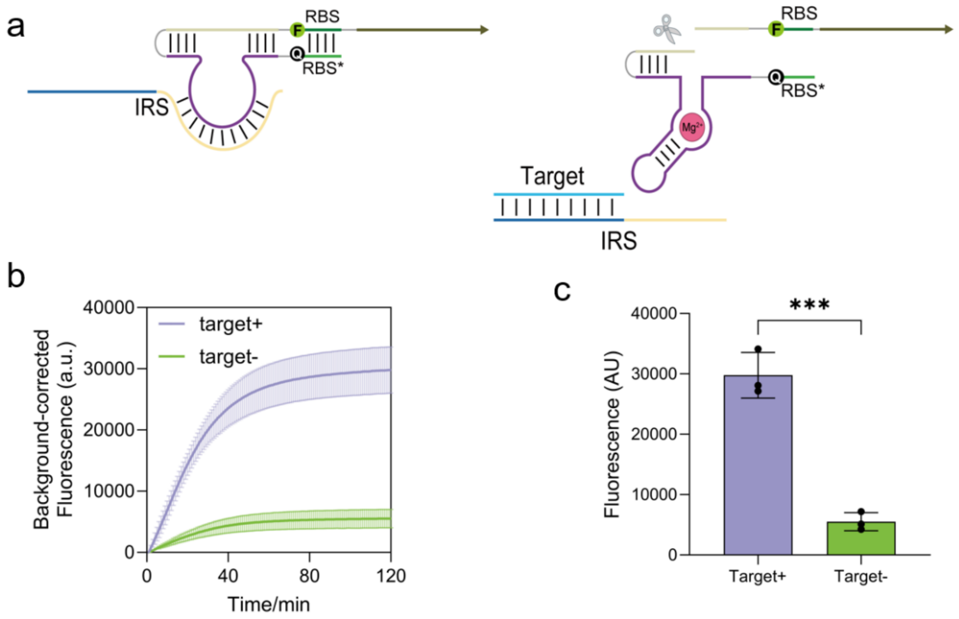

Supplementary Fig. 3 | FRET-based assessment of TRACKer conformational changes upon target binding. a, Schematic illustration of TRACKer for studying molecular conformational changes after target addition, with FAM and BHQ1 labeled at RBS and RBS*, respectively. **b,** Time-dependent fluorescence curves in the presence or absence of the target. **c,** Endpoint fluorescence intensities with or without the target. Shaded regions represent mean ± SD from n = 3 technical replicates (***P = 0.0005, unpaired, 2-tailed Students's t test).

**HiBiT switch**	+					+	+	+
**IRS18-1**		+				+		
**IRS18-2**			+				+	
**IRS18-3**				+				+
**FluA**					+	+	+	+
	**1**	**2**	**3**	**4**	**5**	**6**	**7**	**8**

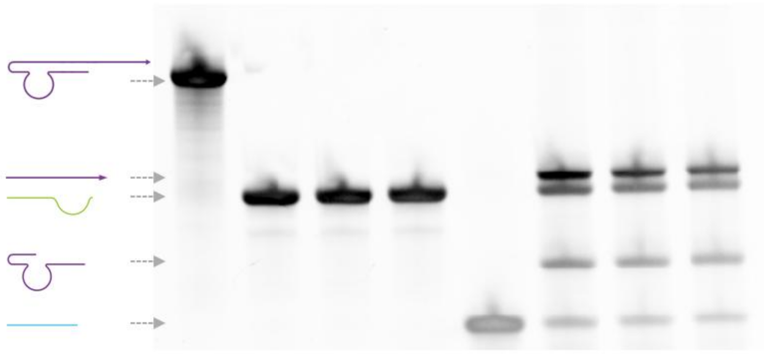

Supplementary Fig. 8 | Urea-TBE Polyacrylamide gel electrophoresis analysis for TRACKer.

(Lane 1) A long strand containing complementary sequences of ribosome- binding sites(RBS*), ribozymes and their substrates, ribosome binding site (RBS), and HiBiT templates, abbreviated as HiBiT switch

(Lane 2) IRS18-1 that can recognize FluA

(Lane 3) IRS18-2 that can recognize FluA

(Lane 4) IRS18-3 that can recognize FluA

(Lane 5) FluA

(Lane 6) When FluA was successfully recognized by IRS18-1, the ribozyme cleaved the substrate, splitting the HiBiT switch into two parts.

(Lane 7) When FluA was successfully recognized by IRS18-2, the ribozyme cleaved the substrate, splitting the HiBiT switch into two parts.

(Lane 8) When FluA was successfully recognized by IRS18-3, the ribozyme cleaved the substrate, splitting the HiBiT switch into two parts.

All the sequences used can be found in Supplementary Table 1.

Response to reviewers

To reviewer 2:

The authors have comprehensively addressed the reviewers' comments. However, some uncertainty remains regarding the underlying mechanism, which was also raised by another reviewer. All other comments have been satisfactorily addressed.

Comment #1

The mechanistic inconsistencies have been partially resolved. The authors have incorporated necessary clarifications regarding simulation parameters, the consistency of ΔG comparisons across IRS lengths, and the interpretation of ternary complex formation. Supplementary Figures 5-7, together with the MD simulation analyses, now provide a coherent rationale regarding the relative binding affinities of the IRS-HH15 and IRS-target complexes.

However, an explanation regarding the instability of the ternary IRS-ribozyme-target complex remains elusive. Specifically, the driving force for target-induced strand displacement remains unclear, as there appears to be no obvious energetic advantage favoring competition between the target and the ribozyme for the IRS (as also asked by another reviewer).

Notwithstanding this theoretical ambiguity, the experimental findings—such as those illustrated in Supplementary Fig. 8—empirically substantiate the authors' claims. Therefore, this mechanistic uncertainty becomes more important.

Reply: We sincerely apologize for the confusion caused by the inaccurate description. The strand displacement mechanism of TRACKer has been shown in supplementary Fig. 3. The HiBiT switch contains a 12-nt “exchange domain” (yellow-highlighted) identical to the target’s first 12 bases, which pre-hybridizes to the IRS in the OFF state. Upon target binding to the IRS’s exposed toehold region, it colocalizes to the exchange domain, invades the IRS-switch duplex via branch migration, displaces the HiBiT switch, and forms a stable target-IRS duplex. This is consistent with the classical strand displacement framework described by Zhang and Winfree (J. Am. Chem. Soc. 2009, 131, 17303-17314). Relevant descriptions of these modifications have been added to the manuscript (lines 111-112,118-121, page 6).

In addition, Native PAGE directly validated the strand displacement mechanism in supplementary Fig. 9: lane 5 shows the preassembled IRS-switch duplex, while lane 6 (FluA + IRS18-1 + HiBiT switch) shows dominant target-IRS duplex and released HiBiT switch, demonstrating no ternary IRS-ribozyme-target complex accumulation. Relevant descriptions of these findings have been added to the manuscript (lines 160-162, page 8).

Further, NUPACK-based thermodynamic screening provides a consistent explanation for the ternary complex’s instability in supplementary Figs. 6-8. The stable ternary complexes become significant only when the IRS-HH15 complementarity becomes too long, while IRS-HH15 complementarity within the 10-18 nt design window avoid strong kinetic trapping and allow productive displacement.

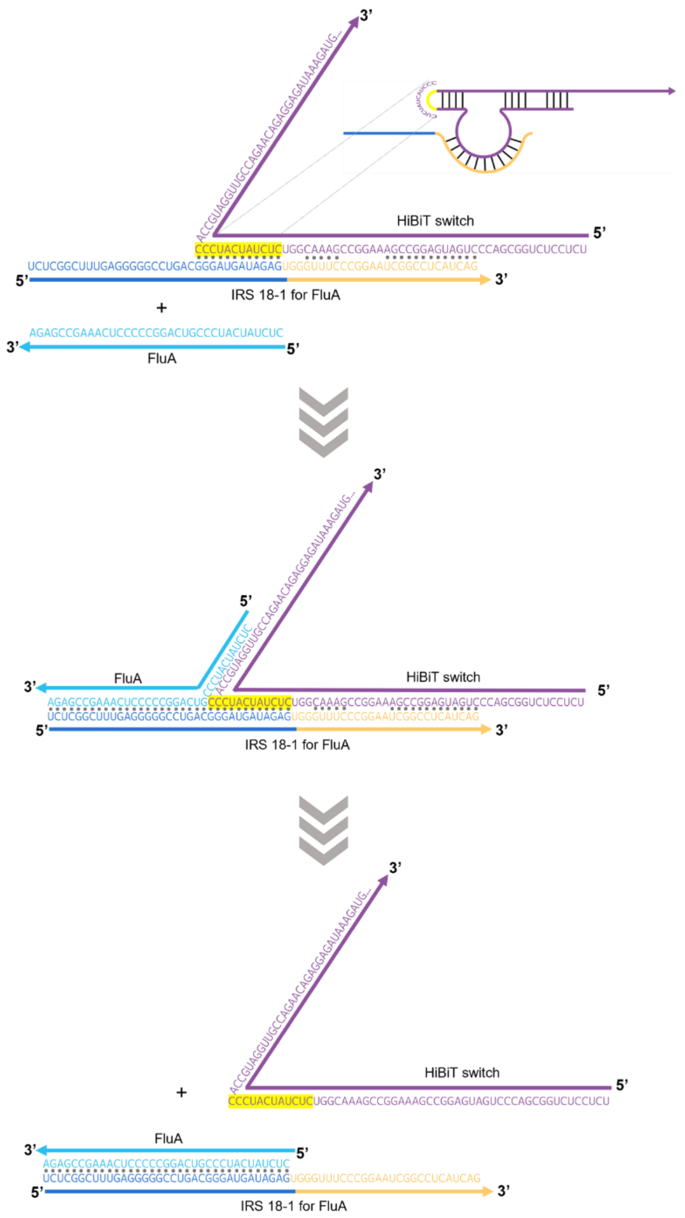

Supplementary Fig. 3| Schematic diagram of the strand displacement mechanism of TRACKer. The yellow region is identical to the first 12 bases of the FluA.

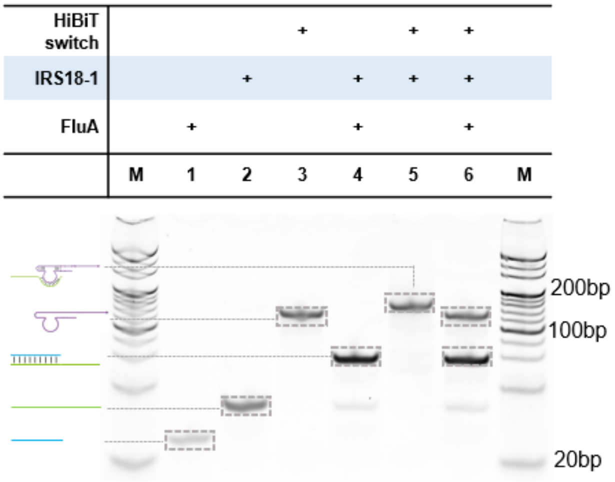

Supplementary Fig. 9 | Native Polyacrylamide gel electrophoresis analysis for the strand displacement manchanism of TRACKer.

(Lane 1) FluA

(Lane 2) IRS18-1 that can recognize FluA

(Lane 3) A long strand containing complementary sequences of ribosome- binding sites(RBS*), ribozymes and their substrates, ribosome binding site(RBS), and HiBiT templates, abbreviated as HiBiT switch

(Lane 4) Hybridization of IRS18-1 with FluA

(Lane 5) Hybridization of IRS18-1 with HiBiT switch

(Lane 6) Hybridization of FluA, IRS18-1 and HiBiT switch

To reviewer 4:

The authors performed MD simulations and PAGE analyses to support the proposed TRACKer mechanism. However, based on the current data, this reviewer still has reservations about the mechanistic interpretation. Specifically, the simulations do not clearly explain why hybridization of the target strand to the IRS leads to displacement

of the IRS from the ribozyme loop region. Consistent with the concerns raised by Reviewer 2, this reviewer has not previously encountered a scenario in which target–IRS pairing—which does not directly compete with the IRS–ribozyme loop interactions—would nevertheless be sufficient to dissociate the IRS from the loop. This observation challenges this reviewer's understanding of the classical principles governing nucleic-acid strand association and dissociation.

Reply: We are grateful for the reviewer's suggestions. In TRACKer, the target directly initiates a classical strand-displacement process as shown in supplementary Fig. 3. The HiBiT switch contains a 12-nt exchange domain (highlighted in yellow) that is identical to the 5′ 12-nt segment of the target and is base-paired with the complementary region of the IRS in the OFF state. Upon target binding to the IRS's exposed toehold region, it colocalizes to the exchange domain, invades the IRS-switch duplex via branch migration, displaces the HiBiT switch, and forms a stable target-IRS duplex. Relevant descriptions of these modifications have been added to the manuscript (lines 111-112,118-121, page 6).

The above process follows the established principles of toehold-mediated strand displacement, as described by Zhang and Winfree (J. Am. Chem. Soc. 2009, 131, 17303 – 17314): (i) toehold binding colocalizes the invading strand, (ii) branch migration proceeds across the exchange domain, and (iii) dissociation of the incumbent strand generates the displaced products.

Additionally, native PAGE provides direct support for this mechanism (Supplementary

Fig. 9). Specifically, the lane 6 (FluA + IRS18-1 + HiBiT switch) shows formation of the target-IRS duplex together with the released HiBiT switch, consistent with target-induced strand displacement rather than accumulation of a stable ternary complex. Consistent with this observation, MD simulations (System 5) show progressive association of the target with the IRS and concomitant dissociation of the IRS from the switch (Supplementary Fig. 11a). Relevant descriptions of these findings have been added to the manuscript (lines 160-162, page 8).

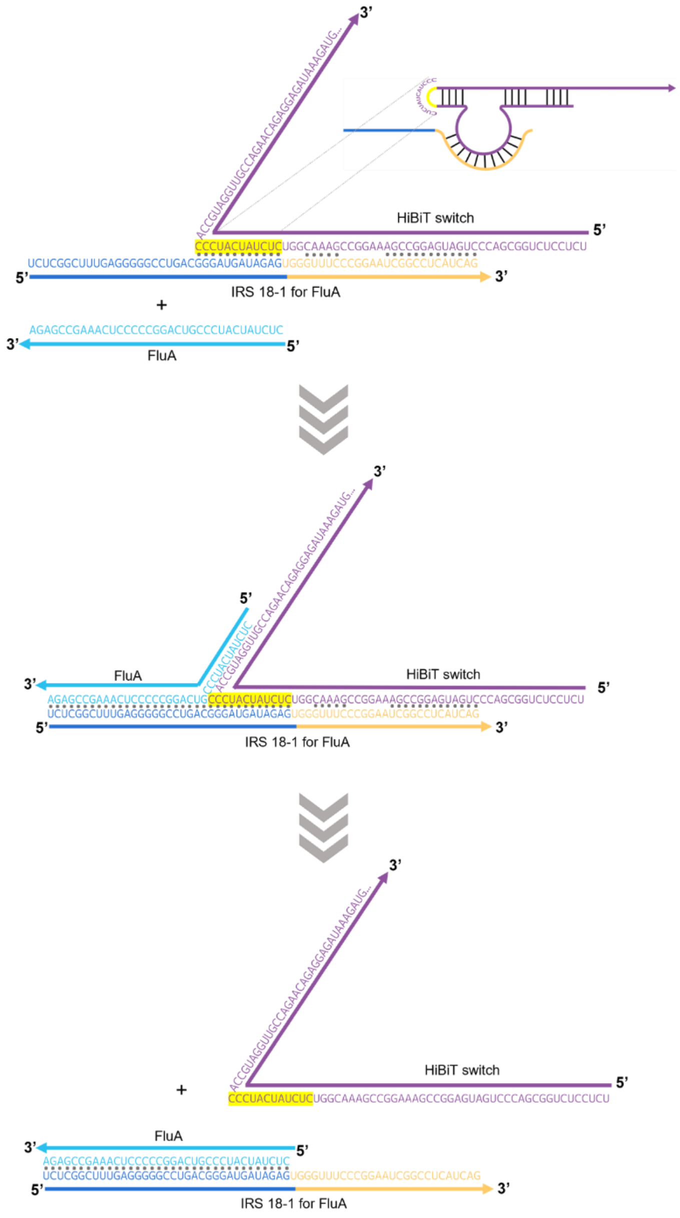

Supplementary Fig. 3| Schematic diagram of the strand displacement mechanism of TRACKer. The yellow region is identical to the first 12 bases of the FluA.

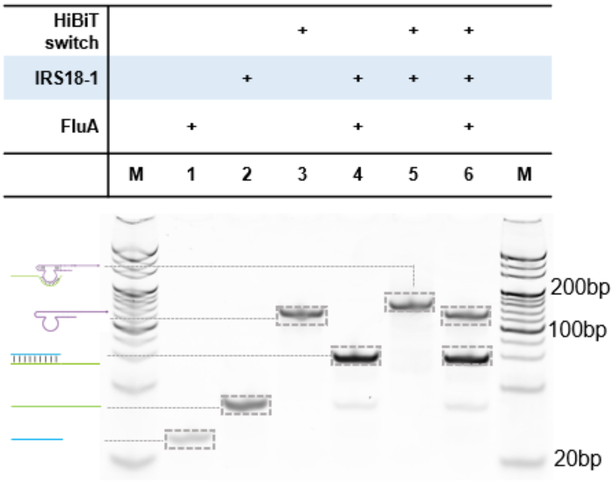

Supplementary Fig. 9 | Native Polyacrylamide gel electrophoresis analysis for the strand displacement manchanism of TRACKer.

(Lane 1) FluA

(Lane 2) IRS18-1 that can recognize FluA

(Lane 3) A long strand containing complementary sequences of ribosome- binding sites(RBS*), ribozymes and their substrates, ribosome binding site(RBS), and HiBiT templates, abbreviated as HiBiT switch

(Lane 4) Hybridization of IRS18-1 with FluA

(Lane 5) Hybridization of IRS18-1 with HiBiT switch

(Lane 6) Hybridization of FluA, IRS18-1 and HiBiT switch

Response to reviewers

To reviewer 4:

The authors have provided the additional clarification regarding the proposed toehold-mediated strand-displacement mechanism. While I appreciate the effort to address the mechanistic concerns, I remain unconvinced that the current explanation satisfactorily resolves the key issues.

1. Omission of the 12-nt exchange domain in earlier versions

According to the revised explanation, the 12-nt exchange domain—base-paired between the IRS and the riboswitch loop in the OFF state—is central to the proposed strand-displacement mechanism. The target initiates branch migration via the toehold, invades this 12-nt duplex, and ultimately displaces the incumbent strand.

If this 12-nt pairing is indeed mechanistically essential, it represents a critical structural determinant of the system. However, this feature was not clearly described in the original submission: 1) It was not emphasized in previous revisions, 2) It was only introduced after repeated concerns from two reviewers, 3) Moreover, this interaction is still not clearly illustrated in the main mechanistic figures (e.g., Figures 1 and 3), which gives the impression of a non-competitive or unconventional recognition mechanism. In its current presentation, the figures remain potentially misleading, as they do not transparently depict the competitive strand-displacement process now being invoked.

Given the centrality of this 12-nt duplex to the proposed mechanism, its late introduction and incomplete graphical representation raise substantial concerns about the clarity and robustness of the mechanistic model.

2. Stability of the residual 14-bp duplex

Even accepting the authors' proposed toehold-mediated strand-displacement model, a second issue remains unresolved. According to Supplementary Fig. 3, after branch migration across the 12-nt exchange domain, the IRS–target duplex remains associated with the riboswitch via ~14 nt of complementary pairing between IRS and the riboswitch loop domain. A 14-bp RNA duplex is typically thermodynamically stable under physiological conditions. It is therefore unclear why the IRS–target complex would dissociate from the riboswitch entirely. The manuscript does not provide sufficient thermodynamic analysis, kinetic evidence, or structural validation to explain how this remaining duplex becomes destabilized. Thus, even with the revised mechanistic explanation, the fundamental question remains: What drives complete IRS dissociation despite the presence of a substantial remaining duplex?

Reply:

1. We sincerely apologize for our earlier misinterpretation of the reviewer comments. Our previous response focused primarily on thermodynamic evidence and molecular dynamics simulations to support the competitive strand-displacement mechanism, but inadvertently underemphasized the role of the 12-nt exchange domain. We have highlighted the 12-nt exchange domain in Figs. 1 and 3 and all other figures describing the competitive strand displacement process. Supplementary Fig. 8 has been newly added to clearly illustrate the strand-displacement principle and the function of the 12-nt exchange domain. Furthermore, we have strengthened the description of the competitive strand-displacement

mechanism in the figure legends of Figs. 1 and 3 and in the revised manuscript (lines 163-171, page 8).

2. Regarding the residual 14-bp duplex stability: The competitive strand-displacement mechanism of TRACKer is explicitly illustrated in the newly added Supplementary Fig. 8. This mechanism is well-established based on the classic work by Zhang and Winfree (J. Am. Chem. Soc. 2009, 131, 17303–17314). As described, Strand X (m, n) binds to complex S via the invading toehold γ^n and displaces strand Y's β_m domain through branch migration. Strand Y's incumbent toehold β^m then spontaneously dissociates, yielding free strand Y and complex L(m, n). Notably, the reaction proceeds to completion when the length of the invading toehold (γ^n, n) exceeds that of the incumbent toehold (β^m, m).

   In our TRACKer system, the invading toehold (23 nt) is longer than the incumbent toehold (14 nt), ensuring that the reaction preferentially generates Y (HiBiT switch) and complex L (FluA + IRS 18-1).This conclusion is further supported by NUPACK-predicted equilibrium concentrations (Supplementary Fig. 9) and native PAGE results (Supplementary Fig. 10), both verifying that target binding triggers competitive strand displacement, forming the target–IRS 18-1 duplex and releasing the HiBiT switch. These results have been incorporated into the revised manuscript (lines 163–171, page 8).

   In conclusion, the well-established mechanism from the JACS publication, together with our NUPACK simulations and PAGE data, consistently demonstrates that the strand-displacement reaction proceeds efficiently and drives complete dissociation

of the IRS.

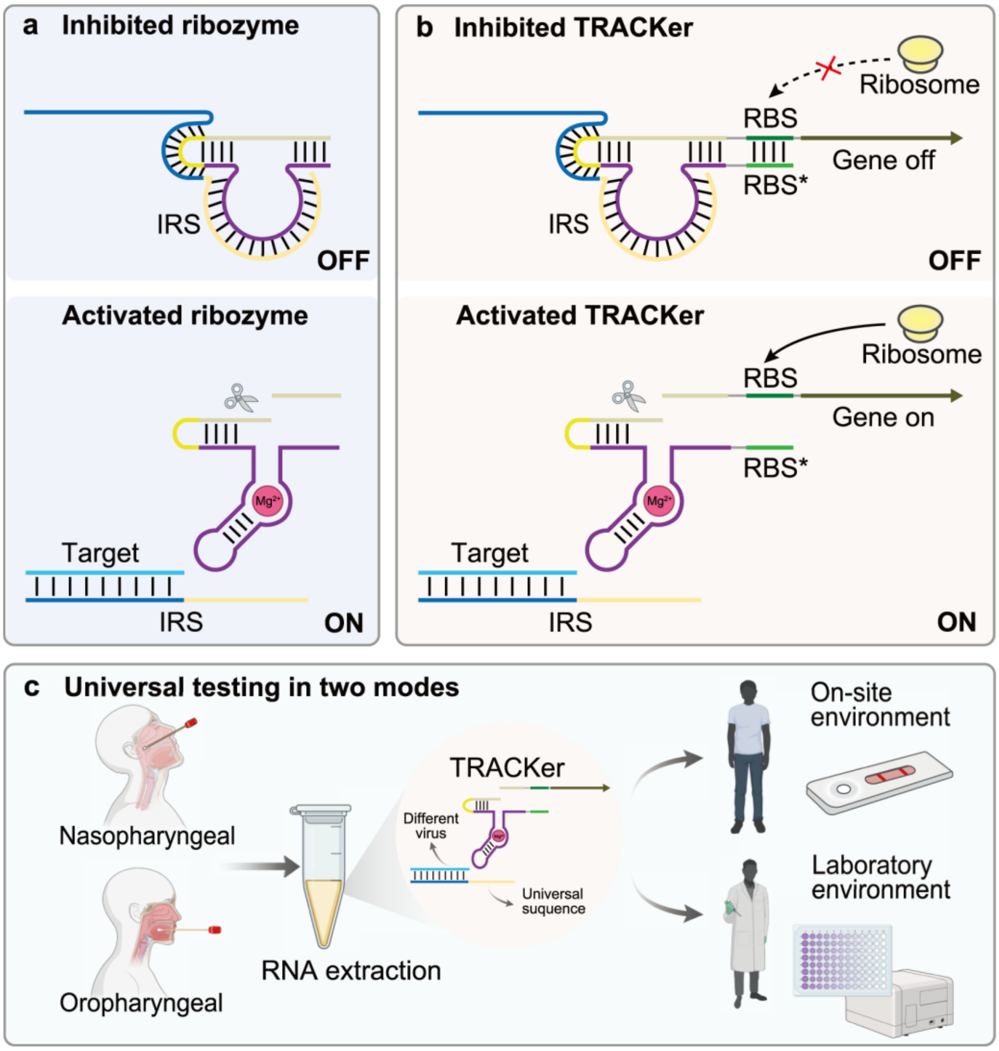

Fig.1 | Overview of TRACKer. **a,** Ribozyme OFF-to-ON transition: the ribozyme is inhibited (OFF) in the absence of target RNA; it activates (ON) and initiates RNA cleavage when target RNA binds. **b,** TRACKer activation via competitive strand displacement: target RNA binds IRS, outcompeting the switch strand and displacing IRS. This releases the ribozyme to cleave its substrate, exposing RBS and initiating reporter translation. **c,** Versatile detection applications: TRACKer accommodates diverse targets through simple replacement of the IRS stand and supports both laboratory and onsite settings. RBS, RBS*, IRS represent Ribosome-binding site, complementary sequences of Ribosome-binding site, and Inhibition-Recognition strand, respectively.

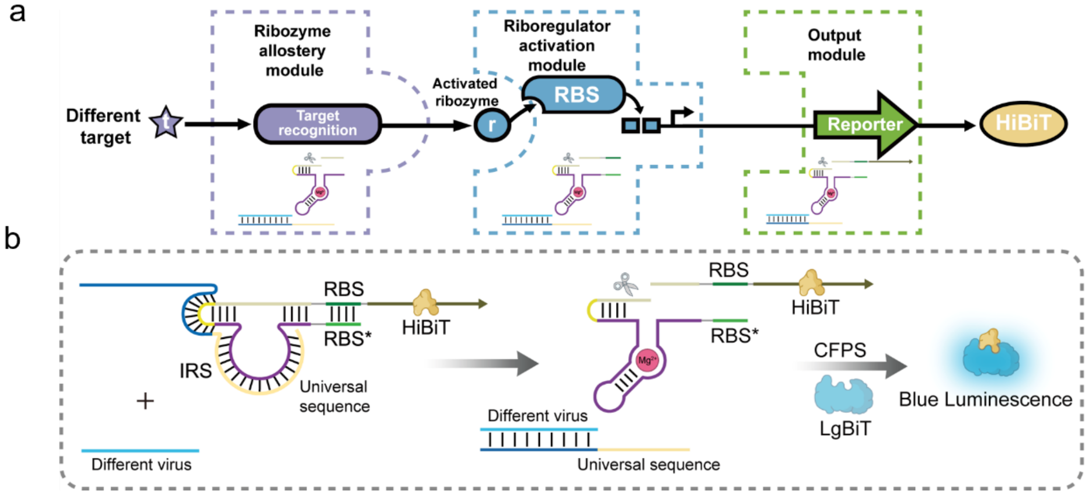

Fig.3 | De novo design and validation of TRACKer. a, TRACKer integrates three modules: ribozyme allostery module, riboregulator activation module, and output module. **b,** Schematic of TRACKer activation via competitive strand displacement: target RNA binds IRS, outcompeting the switch strand and displacing IRS. This frees the ribozyme to cleave its substrate, exposing the RBS and enabling HiBiT translation. HiBiT complements with LgBiT to form active nanoluciferase, which converts furimazine into blue luminescence.

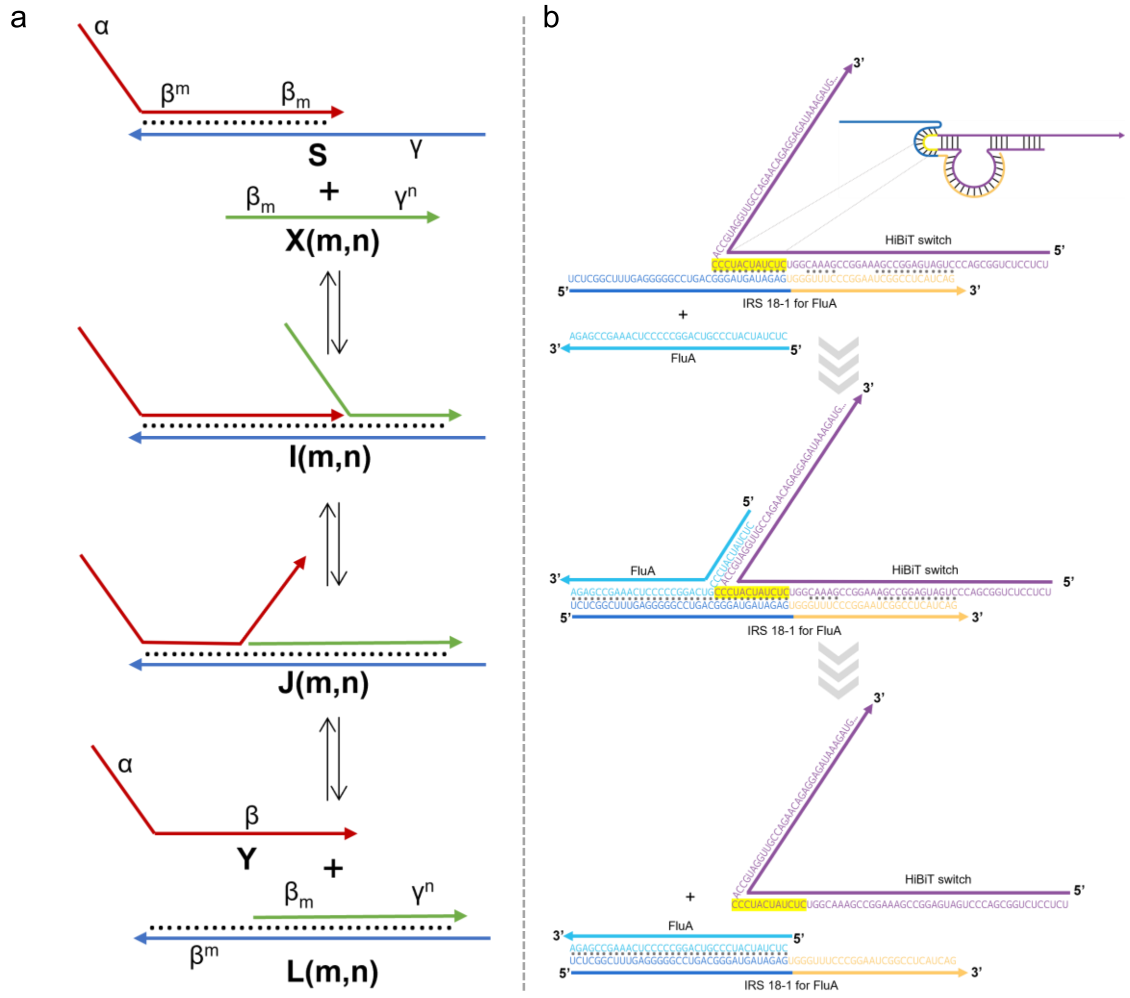

Supplementary Fig. 8| Strand displacement mechanism of TRACKer based on the principle of toehold exchange. a, Toehold exchange reaction established by *Zhang* and *Winfree*. Invading strand X(m, n) binds to substrate complex S by toehold γ^n (known as the invading toehold) to form intermediate I(m, n). Intermediate I(m, n) represents all branch migration states in which Y is bound to more bases of β_m than X(m, n). Intermediate I(m, n) rearranges to form intermediate J(m, n), which analogously represents all states in which X(m, n) binds more bases of β_m than Y. Domain β^m (known as the incumbent toehold) spontaneously dissociates, releasing products Y and L(m, n). The reaction favors product formation when the length of invading toehold n exceeds that of incumbent toehold m. (Adapted with permission from *J. Am. Chem. Soc. 2009, 131, 17303-17314*). **b**, Schematic of the TRACKer strand displacement mechanism. Following the toehold-exchange principle, TRACKer is designed with a 23-nt invading toehold (n = 23) and a 14-nt incumbent toehold (m = 14). The linker between the ribozyme and substrate (yellow region) acts as the branch migration initiation site during competitive strand displacement. With n > m, the reaction favors the forward pathway, yielding the Y strand (HiBiT switch) and L complex (FluA + IRS 18-1).

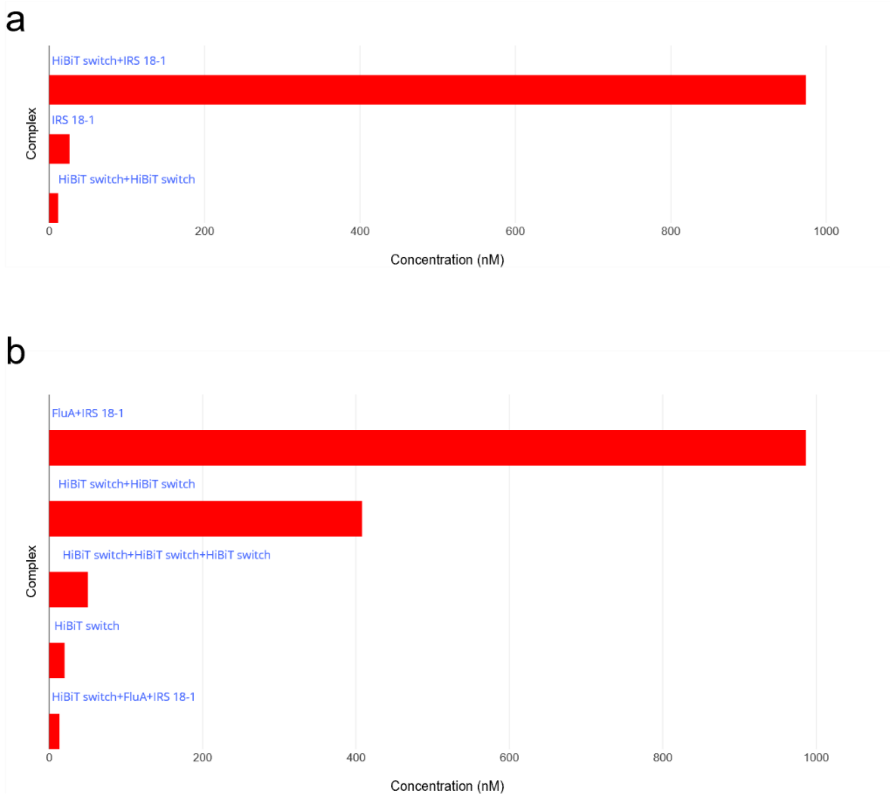

Supplementary Fig.9 NUPACK-predicted equilibrium complex concentrations of (a, HiBiT switch + IRS 18-1) and (b, HiBiT switch + IRS 18-1 + FluA). The structure was predicted using NUPACK based on the conditions as follows: The nucleic acid type was chosen to be 'RNA'; the 'dangles' parameter was set to 'all'; the temperature was set to 31 °C (the temperature at which TRACKer assay was performed).

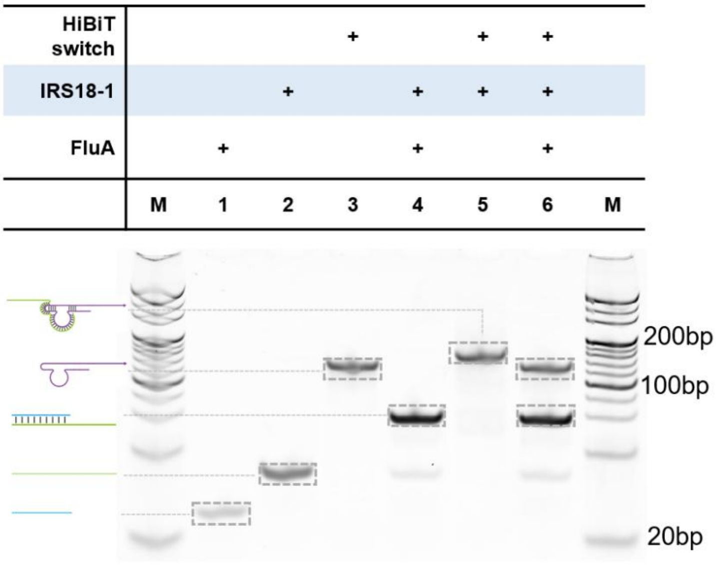

Supplementary Fig. 10 | Native Polyacrylamide gel electrophoresis analysis for the strand displacement manchanism of TRACKer.

(Lane 1) FluA

(Lane 2) IRS18-1 that can recognize FluA

(Lane 3) A long strand containing complementary sequences of ribosome- binding sites(RBS*), ribozymes and their substrates, ribosome binding site(RBS), and HiBiT templates, abbreviated as HiBiT switch

(Lane 4) Hybridization of IRS18-1 with FluA

(Lane 5) Hybridization of IRS18-1 with HiBiT switch

(Lane 6) Hybridization of FluA, IRS18-1 and HiBiT switch

Response to reviewers

To reviewer 5:

> I believe that the authors have sufficiently addressed Reviewer #4's concerns about the toehold-mediated strand displacement. The gel in Supp. Fig. 10 is convincing experimentally. From thermodynamic theory/modeling, the binding of the Hibit switch region after Target binding involves 3 mismatches, then 5 paired bases (4 stacks), then 6 mismatches, then 13 paired bases (12 stacks). With high likelihood, the first group of 5 base pairs will not be stable given the fact the 3 middle bases are AAA|UUU, which are actually destabilizing base stacks for dsRNA. 12 base stacks is marginally stable at 37C, consistent with Reviewer #4's understanding, but that assumes thermodynamic equilibrium. With the depletion of products due to cleavage of the product, the riboswitch + Target binding becomes an irreversible reaction that seems reasonable to complete on the hour timescale.

Reply: Thank you for your positive and insightful comments. We appreciate your thorough analysis of the toehold-mediated strand displacement mechanism in our TRACKer system.
